# Supplementary material for: Estimating maternity ward birth attendant time use in India: a microcosting study
Source: BMJ Open. 2022 Feb 7;12(2):e054164. doi: 10.1136/bmjopen-2021-054164 (PMC8823136; doi:10.1136/bmjopen-2021-054164)

Supplementary Appendix

Estimating maternity ward birth attendant time use in India: a microcosting study

Katherine T Lofgren, Lauren Bobanski, Danielle E Tuller, Vinay P Singh, Megan Marx Delaney, Amanda Jurczak, Meera Ragavan, Tapan Kalita, Ami Karlage, Stephen Charles Resch, Katherine E A Semrau

Appendix Table A1: Activities measured by data collection method

Master (18)	Work Sampling (16)	Time Motion (17)	Time Use (14)
Temperature	Temperature	Temperature	Temperature
Blood pressure	Blood pressure	Blood Pressure	N/A
Partograph	Partograph	Partograph	Partograph
Paper checklist interaction	Checklist/ poster	Paper checklist interaction	Checklist/ poster
Medication	Medication	Admin. Antibiotics/ Admin. Vaccines	Medication
Handwashing	Handwash gloves or alcohol rub	Handwashing	Handwash gloves or alcohol rub
Preparation of essential supplies	Prep of EBS	Prep of essential supplies	Prep of EBS
Neonatal bag mask	Use neonatal bag mask	Neonatal bag	Use neonatal bag mask
Referral	Referring a patient	Referral	Referring a patient
Check mother for bleeding	Check Mother for bleeding	Check mother for bleeding	Check mother for bleeding
Examine newborn	Examination of Newborn (BA)<br>Examination of Newborn (ASHA)	Examine newborn<br>Examine newborn for danger signs	Examination of newborn<br>N/A
Skin-to-skin initiation	Initiation of skin-to-skin	Init. of skin-to-skin	N/A
Discuss family planning	Discussing family planning<br>Group discussion	Discuss family planning<br>Discuss family planning (Group)	Discussing family planning<br>N/A
Explain danger signs	Explaining danger signs	Explain danger signs	Explaining danger signs
Breastfeeding initiation	Initiation of breastfeeding	Init. of breastfeeding	Initiation of breastfeeding
Confirm vaccination	Confirmation of vaccination	N/A	Confirmation of vaccination
Weight	N/A	Weight	N/A
Check baby's breathing	N/A	Assess Baby's Breathing	N/A

Appendix Table A2: Time Motion Observation Tool
(see next page for start of PDF)

Time Motion Observation Tool

A	**Facility Code**	
B	**Date of Observation (DD/MMM/YYYY)**	____/____/____
C	**Health Care Worker Unique ID**	
D	**Health Care Worker Cadre**	☐ Doctor ☐ L.H.V ☐ A.N.M ☐ Staff Nurse ☐ Other
E	**Years of experience as a Health Care Worker**	________Years _______Months
F	**Years of experience as a Health Care Worker at this health facility**	________Years _______Months
G	**Did Health Care Worker consent to Observation**	___Yes ___No
H	**Notes about Time Motion observations:**	
I	**Tool ID Code**	
J	**FADA Employee ID**	
K	**FADA Role**	☐ First Observer ☐ DQA Observer
L	**FADA Start Time**	______:______
M	**FADA End Time**	______:______

Lofgren KT, *et al. BMJ Open* 2022; 12:e054164. doi: 10.1136/bmjopen-2021-054164

	Patient Yearly Number___________<br><br>From which register did you get the yearly number?<br>❑ Delivery<br>❑ Referred Out<br>❑ Admission				Patient Yearly Number _____________<br><br>From which register did you get the yearly number?<br>❑ Delivery<br>❑ Referred Out<br>❑ Admission				Patient Yearly Number __________<br><br>From which register did you get the yearly number?<br>❑ Delivery<br>❑ Referred Out<br>❑ Admission				General Activity (cannot be assigned to specific patient)
	PP 1	PP 2	PP 3	PP 4	PP 1	PP 2	PP 3	PP 4	PP 1	PP 2	PP 3	PP 4	
**Maternal Temperature**													
**Blood Pressure**													
**Partograph interaction**													
**Paper checklist or poster interaction**													
**Administration of antibiotics, magnesium sulfate, oxytocin or antiretroviral**													
**Hand washing, clean gloves or alcohol rub**													
**Preparation of Essential Supplies at bedside table**													
**Use of neonatal bag and mask for baby**													
**Referring a patient**													

Lofgren KT, *et al. BMJ Open* 2022; 12:e054164. doi: 10.1136/bmjopen-2021-054164

	**Patient Yearly Number___________**<br><br>**From which register did you get the yearly number?**<br>❑ **Delivery**<br>❑ **Referred Out**<br>❑ **Admission**				**Patient Yearly Number _____________**<br><br>**From which register did you get the yearly number?**<br>❑ **Delivery**<br>❑ **Referred Out**<br>❑ **Admission**				**Patient Yearly Number __________**<br><br>**From which register did you get the yearly number?**<br>❑ **Delivery**<br>❑ **Referred Out**<br>❑ **Admission**				**General Activity (cannot be assigned to specific patient)**
	PP 1	PP 2	PP 3	PP 4	PP 1	PP 2	PP 3	PP 4	PP 1	PP 2	PP 3	PP 4	
**Check mother for bleeding**													
**Examination of Newborn**													
Examine the Baby for Danger Signs													
Assess Baby's Breathing													
Take Baby's Temperature													
Take Baby's Weight													
Monitor the Baby in order to Take Appropriate Action for Special Care (Requires Resuscitation)													
**Initiation of skin-to-skin**													

	**Patient Yearly Number___________**<br>**From which register did you get the yearly number?**<br>❑ **Delivery**<br>❑ **Referred Out**<br>❑ **Admission**				**Patient Yearly Number _____________**<br>**From which register did you get the yearly number?**<br>❑ **Delivery**<br>❑ **Referred Out**<br>❑ **Admission**				**Patient Yearly Number __________**<br>**From which register did you get the yearly number?**<br>❑ **Delivery**<br>❑ **Referred Out**<br>❑ **Admission**				**General Activity (cannot be assigned to specific patient)**
	PP 1	PP 2	PP 3	PP 4	PP 1	PP 2	PP 3	PP 4	PP 1	PP 2	PP 3	PP 4	
**Discussing Family Planning**													
**Explaining Danger Signs for mother and child**													
Group Discussion (if family planning and danger signs could not be observed)													
**Initiation of breastfeeding**													
**Administration of Vaccination**													

Lofgren KT, *et al. BMJ Open* 2022; 12:e054164. doi: 10.1136/bmjopen-2021-054164

	**Patient Yearly Number___________**<br><br>**From which register did you get the yearly number?**<br>❑ **Delivery**<br>❑ **Referred Out**<br>❑ **Admission**				**Patient Yearly Number _____________**<br><br>**From which register did you get the yearly number?**<br>❑ **Delivery**<br>❑ **Referred Out**<br>❑ **Admission**				**Patient Yearly Number __________**<br><br>**From which register did you get the yearly number?**<br>❑ **Delivery**<br>❑ **Referred Out**<br>❑ **Admission**				**General Activity (cannot be assigned to specific patient)**
	PP 1	PP 2	PP 3	PP 4	PP 1	PP 2	PP 3	PP 4	PP 1	PP 2	PP 3	PP 4	
**Maternal Temperature**													
**Blood Pressure**													
**Partograph interaction**													
**Paper checklist or poster interaction**													
**Administration of antibiotics, magnesium sulfate, oxytocin or antiretroviral**													
**Hand washing, clean gloves or alcohol rub**													
**Preparation of Essential Supplies at bedside table**													
**Use of neonatal bag and mask for baby**													
**Referring a patient**													

5

BB CEA Time Motion 29Apr2016

	**Patient Yearly Number___________ From which register did you get the yearly number? ❑ Delivery ❑ Referred Out ❑ Admission**				**Patient Yearly Number ____________ From which register did you get the yearly number? ❑ Delivery ❑ Referred Out ❑ Admission**				**Patient Yearly Number __________ From which register did you get the yearly number? ❑ Delivery ❑ Referred Out ❑ Admission**				**General Activity (cannot be assigned to specific patient)**
	PP 1	PP 2	PP 3	PP 4	PP 1	PP 2	PP 3	PP 4	PP 1	PP 2	PP 3	PP 4	
**Check mother for bleeding**													
**Examination of Newborn**													
Examine the Baby for Danger Signs													
Assess Baby's Breathing													
Take Baby's Temperature													
Take Baby's Weight													
Monitor the Baby in order to Take Appropriate Action for Special Care (Requires Resuscitation)													
**Initiation of skin-to-skin**													

Lofgren KT, *et al. BMJ Open* 2022; 12:e054164. doi: 10.1136/bmjopen-2021-054164

	**Patient Yearly Number___________**<br><br>**From which register did you get the yearly number?**<br>❑ **Delivery**<br>❑ **Referred Out**<br>❑ **Admission**				**Patient Yearly Number _____________**<br><br>**From which register did you get the yearly number?**<br>❑ **Delivery**<br>❑ **Referred Out**<br>❑ **Admission**				**Patient Yearly Number __________**<br><br>**From which register did you get the yearly number?**<br>❑ **Delivery**<br>❑ **Referred Out**<br>❑ **Admission**				**General Activity (cannot be assigned to specific patient)**
	PP 1	PP 2	PP 3	PP 4	PP 1	PP 2	PP 3	PP 4	PP 1	PP 2	PP 3	PP 4	
**Discussing Family Planning**													
**Explaining Danger Signs for mother and child**													
Group Discussion (if family planning and danger signs could not be observed)													
**Initiation of breastfeeding**													
**Administration of Vaccination**													

Lofgren KT, *et al. BMJ Open* 2022; 12:e054164. doi: 10.1136/bmjopen-2021-054164

Appendix Table A3: Work Sampling Census
(see next page for start of PDF)

BB Work Sampling Census Sheet

Cover Page

A	**Facility Code**	
B	**Date of Observation (DD/MMM/YYYY)**	______/______/______
C	**Notes about Work Sampling Census Observation:**	
D	**FADA Employee ID**	

Work Sampling Census Sheet 29April2016

BB Work Sampling Census Page

Directions: update these numbers when you begin work sampling and in the last 5 minutes of every 2 hours of observation. Record the time and update the numbers based on FADA TL observation only and not official record

Hour	Start	Start + 2	Start + 4	Start + 6	Start + 8	Start + 10				
Clock Time										
Number of Women Admitted During Previous 2 hours										
Number of Women CURRENTLY in Waiting Room										
Number of Women CURRENTLY in L&D										
Number of Women CURRENTLY in Recovery										
Number Women Discharged / Transferred / Died During Previous 2 hours										
Number of Birth Attendants CURRENTLY on Duty in L&D										
Number of Helpers CURRENTLY on Duty in L&D										

Work Sampling Census Sheet 29April2016

Appendix Table A4: Work Sampling Observation Tool
(see next page for start of PDF)

Lofgren KT, *et al. BMJ Open* 2022; 12:e054164. doi: 10.1136/bmjopen-2021-054164

BB Work Sampling Observation Tool

A	**Facility Code**	
B	**Date of Observation (DD/MMM/YYYY)**	____/____/____
C	**Health Care Worker Unique ID**	
D	**Health Care Worker Cadre**	□ Doctor □ L.H.V □ A.N.M □ Staff Nurse □ Other
E	**Years of Experience as a Health Worker**	________ Years ________ Months
F	**Years of Experience as a Health Worker at this health facility**	
G	**Did Health care Worker consent to Observation**	___Yes ___No
H	**Notes about Work Sampling Observation:**	
I	**Tool ID Code**	
J	**FADA Employee ID**	
K	**FADA Role**	□ First Observer □ DQA Observer
L	**FADA Start Time**	______:______
M	**FADA End Time**	______:______

**Patients Consented**	
**Yearly Number**	**From which register did you get the yearly number?**

BB Work Sampling Tool 10Jun2016

WHO Safe Childbirth Checklist Activities																		Non WHO SCC Activities				
Observation Time	1. Mat Temp	2. Blood Pressure	3. Parto.	4. Checklist/ poster	5. Medication	6. Hand-wash gloves or alochol rub	7. Prep of EBS	8. Use neonatal bag mask	9. Referring a patient	10. Check Mother for bleeding	11. Examination of Newborn	12. Initiation of skin-to-skin	13. Discussing Family Planning	14. Danger signs	15. Group Discussion (if family planning and danger signs could not be observed)	16. Initiation of Breast-feeding	17. Confirmation of Vaccination	18. Non-CL Direct Patient Care	19. Admin. Duties	20. Break	21. Downtime	22. Unobserved
HOUR 1																						
:02																						
:04																						
:06																						
:08																						
:10																						
:12																						
:14																						
:16																						
:18																						
:20																						
:22																						
:24																						
:26																						
:28																						
:30																						
:32																						
:34																						
:36																						
:38																						
:40																						
:42																						
:44																						
:46																						
:48																						
:50																						
:52																						
:54																						
:56																						
:58																						

Notes:

1

Observation Time	1. Mat Temp	2. Blood Pressure	3. Parto.	4. Checklist/ poster	5. Medication	6. Handwash gloves or alochol rub	7. Prep of EBS	8. Use neonatal bag mask	9. Referring a patient	10. Check Mother for bleeding	11. Examination of Newborn	12. Initiation of skin-to-skin	13. Discussing Family Planning	14. Danger signs	15. Group Discussion (if family planning and danger signs could not be observed)	16. Initiation of Breast-feeding	17. Confirmation of Vaccination	18. Non-CL Direct Patient Care	19. Admin. Duties	20. Break	21. Downtime	22. Unobserved
HOUR 2																						
:00																						
:02																						
:04																						
:06																						
:08																						
:10																						
:12																						
:14																						
:16																						
:18																						
:20																						
:22																						
:24																						
:26																						
:28																						
:30																						
:32																						
:34																						
:36																						
:38																						
:40																						
:42																						
:44																						
:46																						
:48																						
:50																						
:52																						
:54																						
:56																						
:58																						

Notes:

2

Obser-vation Time	1. Mat Temp	2. Blood Pressure	3. Parto.	4. Checklist/ poster	5. Medication	6. Hand-wash gloves or alochol rub	7. Prep of EBS	8. Use neonatal bag mask	9. Referring a patient	10. Check Mother for bleeding	11. Exami-nation of Newborn	12. Initiation of skin-to-skin	13. Discuss-ing Family Planning	14. Danger signs	15. Group Discussion (if family planning and danger signs could not be observed)	16. Initiation of Breast-feeding	17. Confirm-ation of Vaccination	18. Non-CL Direct Patient Care	19. Admin. Duties	20. Break	21. Down-time	22. Unob served
HOUR 3																						
:00																						
:02																						
:04																						
:06																						
:08																						
:10																						
:12																						
:14																						
:16																						
:18																						
:20																						
:22																						
:24																						
:26																						
:28																						
:30																						
:32																						
:34																						
:36																						
:38																						
:40																						
:42																						
:44																						
:46																						
:48																						
:50																						
:52																						
:54																						
:56																						
:58																						

Notes:

3

Obser-vation Time	1. Mat Temp	2. Blood Pressure	3. Parto.	4. Checklist/ poster	5. Medication	6. Hand-wash gloves or alochol rub	7. Prep of EBS	8. Use neonatal bag mask	9. Referring a patient	10. Check Mother for bleeding	11. Exami-nation of Newborn	12. Initiation of skin-to-skin	13. Discuss-ing Family Planning	14. Danger signs	15. Group Discussion (If family planning and danger signs could not be observed)	16. Initiation of Breast-feeding	17. Confirm-ation of Vaccination	18. Non-CL Direct Patient Care	19. Admin. Duties	20. Break	21. Down-time	22. Unobserved
HOUR 4																						
:00																						
:02																						
:04																						
:06																						
:08																						
:10																						
:12																						
:14																						
:16																						
:18																						
:20																						
:22																						
:24																						
:26																						
:28																						
:30																						
:32																						
:34																						
:36																						
:38																						
:40																						
:42																						
:44																						
:46																						
:48																						
:50																						
:52																						
:54																						
:56																						
:58																						

Notes:

4

Obser-vation Time	1. Mat Temp	2. Blood Pressure	3. Parto.	4. Checklist/ poster	5. Medication	6. Hand-wash gloves or alochol rub	7. Prep of EBS	8. Use neonatal bag mask	9. Referring a patient	10. Check Mother for bleeding	11. Exami-nation of Newborn	12. Initiation of skin-to-skin	13. Discuss-ing Family Planning	14. Danger signs	15. Group Discussion (if family planning and danger signs could not be observed)	16. Initiation of Breast-feeding	17. Confirm-ation of Vaccination	18. Non-CL Direct Patient Care	19. Admin. Duties	20. Break	21. Down-time	22. Unob served
HOUR 5																						
:00																						
:02																						
:04																						
:06																						
:08																						
:10																						
:12																						
:14																						
:16																						
:18																						
:20																						
:22																						
:24																						
:26																						
:28																						
:30																						
:32																						
:34																						
:36																						
:38																						
:40																						
:42																						
:44																						
:46																						
:48																						
:50																						
:52																						
:54																						
:56																						
:58																						

Notes:

5

Obser-vation Time	1. Mat Temp	2. Blood Pressure	3. Parto.	4. Checklist/ poster	5. Medication	6. Hand-wash gloves or alochol rub	7. Prep of EBS	8. Use neonatal bag mask	9. Referring a patient	10. Check Mother for bleeding	11. Exami-nation of Newborn	12. Initiation of skin-to-skin	13. Discuss-ing Family Planning	14. Danger signs	15. Group Discussion (if family planning and danger signs could not be observed)	16. Initiation of Breast-feeding	17. Confirm-ation of Vaccination	18. Non-CL Direct Patient Care	19. Admin. Duties	20. Break	21. Down-time	22. Unob served
HOUR 6																						
:00																						
:02																						
:04																						
:06																						
:08																						
:10																						
:12																						
:14																						
:16																						
:18																						
:20																						
:22																						
:24																						
:26																						
:28																						
:30																						
:32																						
:34																						
:36																						
:38																						
:40																						
:42																						
:44																						
:46																						
:48																						
:50																						
:52																						
:54																						
:56																						
:58																						

Notes:

Obser-vation Time	1. Mat Temp	2. Blood Pressure	3. Parto.	4. Checklist/ poster	5. Medication	6. Hand-wash gloves or alochol rub	7. Prep of EBS	8. Use neonatal bag mask	9. Referring a patient	10. Check Mother for bleeding	11. Exami-nation of Newborn	12. Initiation of skin-to-skin	13. Discuss-ing Family Planning	14. Danger signs	15. Group Discussion (if family planning and danger signs could not be observed)	16. Initiation of Breast-feeding	17. Confirm-ation of Vaccination	18. Non-CL Direct Patient Care	19. Admin. Duties	20. Break	21. Down-time	22. Unob served
HOUR 7																						
:00																						
:02																						
:04																						
:06																						
:08																						
:10																						
:12																						
:14																						
:16																						
:18																						
:20																						
:22																						
:24																						
:26																						
:28																						
:30																						
:32																						
:34																						
:36																						
:38																						
:40																						
:42																						
:44																						
:46																						
:48																						
:50																						
:52																						
:54																						
:56																						
:58																						

Notes:

Obser-vation Time	1. Mat Temp	2. Blood Pressure	3. Parto.	4. Checklist/ poster	5. Medication	6. Hand-wash gloves or alochol rub	7. Prep of EBS	8. Use neonatal bag mask	9. Referring a patient	10. Check Mother for bleeding	11. Exami-nation of Newborn	12. Initiation of skin-to-skin	13. Discuss-ing Family Planning	14. Danger signs	15. Group Discussion (if family planning and danger signs could not be observed)	16. Initiation of Breast-feeding	17. Confirm-ation of Vaccination	18. Non-CL Direct Patient Care	19. Admin. Duties	20. Break	21. Down-time	22. Unobserved
HOUR 8																						
:00																						
:02																						
:04																						
:06																						
:08																						
:10																						
:12																						
:14																						
:16																						
:18																						
:20																						
:22																						
:24																						
:26																						
:28																						
:30																						
:32																						
:34																						
:36																						
:38																						
:40																						
:42																						
:44																						
:46																						
:48																						
:50																						
:52																						
:54																						
:56																						
:58																						

Notes:

8

Appendix Table A5: General task categories in work sampling

General Group	Specific Activity
Checklist (CL)	Temperature<br>Blood pressure<br>Partograph<br>Paper checklist interaction<br>Medication<br>Handwashing<br>Prep of essential supplies<br>Neonatal bag mask<br>Referral<br>Check mother for bleeding<br>Examine newborn<br>Skin-to-skin initiation<br>Discuss family planning<br>Explain danger signs<br>Breastfeeding initiation<br>Confirm vaccination
Non-Checklist Clinical	Non-CL Direct Patient Care
Administrative	Admin. Duties
Downtime	Break<br>Downtime

Lofgren KT, *et al. BMJ Open* 2022; 12:e054164. doi: 10.1136/bmjopen-2021-054164

Appendix Table A6: Task-time estimates for Essential Birth Practices

Essential Birth Practices	Time Source	Mean	SE	Min (seconds)	Max	Sample Size
Referral	Direct measurement	127	16	16	330	21
	Self-report	256	34	90	720	22
Temperature	Direct measurement	94	4	3	275	92
	Self-report	60	0	60	60	2
Check mother for bleeding	Direct measurement	92	12	2	320	37
	Self-report	178	22	60	300	14
Neonatal bag mask use	Direct measurement	76	16	6	300	22
	Self-report	175	23	60	300	11
Blood pressure	Direct measurement	74	2	20	165	126
	Self-report	N/A				N/A
Preparation of essential supplies	Direct measurement	59	18	8	436	31
	Self-report	202	29	120	600	18
Paper checklist interaction	Direct measurement	54	3	4	180	175
	Self-report	210	46	150	300	3
Explain danger signs	Direct measurement	40	4	8	109	41
	Self-report	258	14	60	600	65
Partograph	Direct measurement	38	3	9	88	38
	Self-report	164	16	30	240	15
Discuss family planning	Direct measurement	36	5	4	94	30
	Self-report	316	16	60	600	60
Examine newborn	Direct measurement	34	3	4	177	143
	Self-report	184	30	60	600	17
Assess baby's breathing	Direct measurement	31	6	10	60	9
	Self-report	N/A				N/A
Medication	Direct measurement	29	1	1	150	419
	Self-report	225	75	150	300	2
Handwashing	Direct measurement	29	1	1	81	208
	Self-report	180	11	150	210	6
Breastfeeding initiation	Direct measurement	24	2	6	60	43
	Self-report	223	35	120	420	9
Skin-to-skin initiation	Direct measurement	20	2	3	93	89
	Self-report	N/A				N/A
Weight	Direct measurement	18	2	5	44	35
	Self-report	N/A				N/A

Appendix Table A7: Heat map of self-reported most time-consuming Checklist tasks*

Checklist Activity	Rank 1 (Number of Respondents)	Rank 2	Rank 3	Total
Discussing family planning	41	16	3	60
Explaining danger signs	22	31	12	65
Prep of EBS	5	3	10	18
Check mother for bleeding	3	4	7	14
Initiation of breastfeeding	3	2	4	9
Referring a patient	3	11	8	22
Partograph	2	3	10	15
Use neonatal bag mask	2	1	8	11
Examination of newborn	1	4	12	17
Handwash gloves or alcohol rub	1	3	2	6
Checklist/ poster	0	2	1	3
Confirmation of vaccination	0	2	3	5
Medication	0	1	1	2
Temperature	0	0	2	2

*Number of staff reporting activity in each rank position; total staff interviewed = 83

Lofgren KT, *et al. BMJ Open* 2022; 12:e054164. doi: 10.1136/bmjopen-2021-054164

Appendix Table A8: Figure 2 Sample Sizes (Work-Sampling Observations)

Patient Load	Checklist-based practices		Clinical non-checklist		Downtime		Administrative		Unobserved		Total Observations
	N		N		N		N		N		
No patients	30	1.1%	141	5.0%	2204	78.6%	425	15.2%	3	0.1%	2803
1-2 patients	1037	5.3%	3756	19.1%	9613	48.8%	4934	25.1%	347	1.8%	19687
3+ patients	393	7.4%	963	18.2%	2126	40.3%	1684	31.9%	112	2.1%	5278
											**27768**

Appendix Table A9: Figure 3 Sample Sizes (Facility-Hours)

Patient Load	All Break	Some Break	No Break	Total
No patients	75	44	7	126
1-2 patients	154	614	162	930
3+ patients	22	177	65	264
				**1320**

Appendix Figure A1: Median labor and delivery ward patient load by hour (time of day) (1319 facility-hour observations; one facility-hour dropped in hour 20)

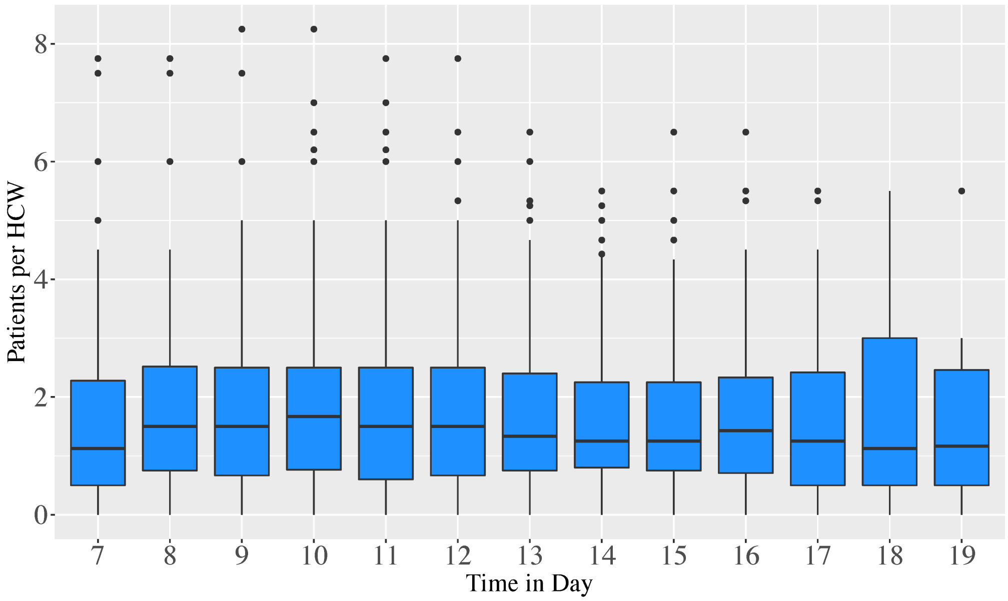

Lofgren KT, *et al. BMJ Open* 2022; 12:e054164. doi: 10.1136/bmjopen-2021-054164

Appendix Figure A2: Median percent of hour on break by patient load per HCW (1320 facility-hour observations)

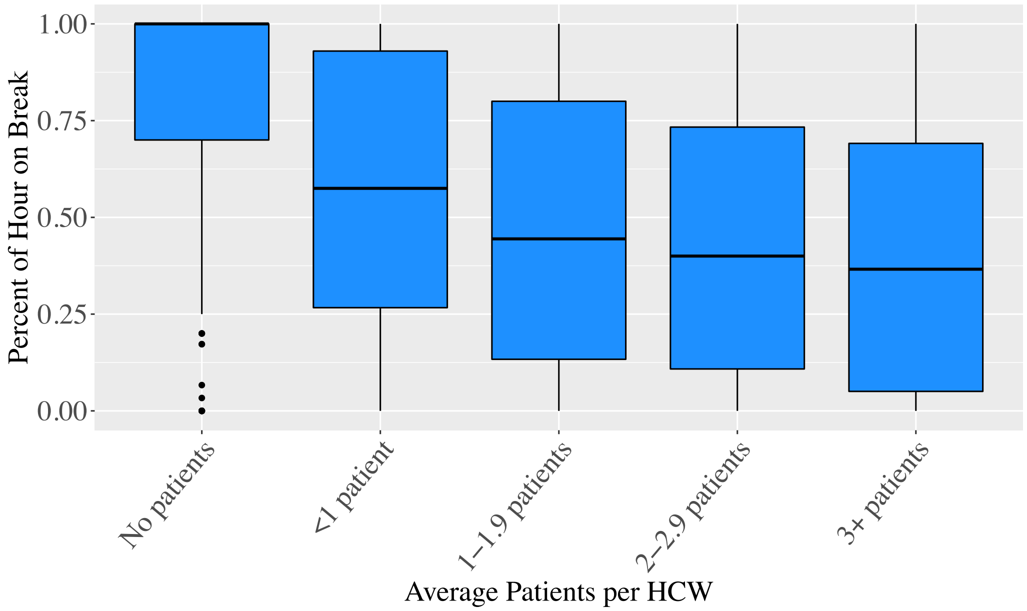

Supplement: Supplementary data [file bmjopen-2021-054164supp001.pdf]
