# Supplementary material for: Estimating maternity ward birth attendant time use in India: a microcosting study
Source: BMJ Open. 2022 Feb 7;12(2):e054164. doi: 10.1136/bmjopen-2021-054164 (PMC8823136; doi:10.1136/bmjopen-2021-054164)
Supplement: Author's manuscript [file bmjopen-2021-054164.draft_revisions.pdf]

BMJ Open is committed to open peer review. As part of this commitment we make the peer review history of every article we publish publicly available.

When an article is published we post the peer reviewers' comments and the authors' responses online. We also post the versions of the paper that were used during peer review. These are the versions that the peer review comments apply to.

The versions of the paper that follow are the versions that were submitted during the peer review process. They are not the versions of record or the final published versions. They should not be cited or distributed as the published version of this manuscript.

BMJ Open is an open access journal and the full, final, typeset and author-corrected version of record of the manuscript is available on our site with no access controls, subscription charges or pay-per-view fees (http://bmjopen.bmj.com).

If you have any questions on BMJ Open's open peer review process please email info.bmjopen@bmj.com

BMJ Open

Labor in labor and delivery wards:Evidence on provider time-use from the BetterBirth Trial

Journal:	*BMJ Open*
Manuscript ID	bmjopen-2021-054164
Article Type:	Original research
Date Submitted by the Author:	15-Jun-2021

Complete List of Authors:	Lofgren, Katherine; Harvard University, Interfaculty Initiative in Health Policy<br>Bobanski , Lauren ; Ariadne Labs, Harvard T.H. Chan School of Public Health/Brigham and Women's Hospital, 401 Park Drive, 3rd floor West<br>Tuller, Danielle; Harvard TH Chan School of Public Health / Brigham and Women's Hospital<br>Singh, Vinay P.; Population Services International; Community Empowerment Lab<br>Marx Delaney , Megan; Ariadne Labs; Harvard T. H. Chan School of Public Health<br>Jurczak, Amanda; Ariadne Labs<br>Ragavan, Meera; University of California San Francisco, Department of Medicine, Division of Hematology/Oncology<br>Kalita, Tapan; Piramal Swasthya Management and Research Institute<br>Karlage, Ami; Ariadne Labs<br>Resch, Stephen; Harvard University T H Chan School of Public Health, Center for Health Decision Science<br>Semrau, Katherine; Brigham and Women's Hospital, Ariadne Labs; Brigham and Women's Hospital, Division of Global Health Equity & Department of Medicine

Keywords:	Health economics < HEALTH SERVICES ADMINISTRATION & MANAGEMENT, Maternal medicine < OBSTETRICS, Quality in health care < HEALTH SERVICES ADMINISTRATION & MANAGEMENT

SCHOLARONE™ Manuscripts

I, the Submitting Author has the right to grant and does grant on behalf of all authors of the Work (as defined in the below author licence), an exclusive licence and/or a non-exclusive licence for contributions from authors who are: i) UK Crown employees; ii) where BMJ has agreed a CC-BY licence shall apply, and/or iii) in accordance with the terms applicable for US Federal Government officers or employees acting as part of their official duties; on a worldwide, perpetual, irrevocable, royalty-free basis to BMJ Publishing Group Ltd ("BMJ") its licensees and where the relevant Journal is co-owned by BMJ to the co-owners of the Journal, to publish the Work in this journal and any other BMJ products and to exploit all rights, as set out in our licence.

The Submitting Author accepts and understands that any supply made under these terms is made by BMJ to the Submitting Author unless you are acting as an employee on behalf of your employer or a postgraduate student of an affiliated institution which is paying any applicable article publishing charge ("APC") for Open Access articles. Where the Submitting Author wishes to make the Work available on an Open Access basis (and intends to pay the relevant APC), the terms of reuse of such Open Access shall be governed by a Creative Commons licence – details of these licences and which Creative Commons licence will apply to this Work are set out in our licence referred to above.

Other than as permitted in any relevant BMJ Author's Self Archiving Policies, I confirm this Work has not been accepted for publication elsewhere, is not being considered for publication elsewhere and does not duplicate material already published. I confirm all authors consent to publication of this Work and authorise the granting of this licence.

For peer review only

Title: Labor in labor and delivery wards: Evidence on provider time-use from the BetterBirth Trial

Corresponding Author:
Katherine T. Lofgren
Harvard University, Interfaculty Initiative in Health Policy
14 Story St.
Cambridge, MA. 02138
kate.lofgren@gmail.com

Co-Authors:
Lauren Bobanski
Ariadne Labs, Brigham & Women's Hospital and Harvard T.H. Chan School of Public Health, Boston, MA., USA
Danielle E. Tuller
Ariadne Labs, Brigham & Women's Hospital and Harvard T.H. Chan School of Public Health, Boston, MA., USA
Vinay P. Singh
Population Services International, Lucknow, India
Community Empowerment Lab, Lucknow, India
Megan Marx Delaney
Ariadne Labs, Brigham & Women's Hospital and Harvard T.H. Chan School of Public Health, Boston, MA., USA
Amanda Jurczak
Ariadne Labs, Brigham & Women's Hospital and Harvard T.H. Chan School of Public Health, Boston, MA., USA
Meera Ragavan
University of California San Francisco, Department of Medicine, Division of Hematology/Oncology, San Francisco, CA.
Tapan Kalita
Piramal Swasthya Management and Research Institute, Hyderabad, India
Ami Karlage
Ariadne Labs, Brigham & Women's Hospital and Harvard T.H. Chan School of Public Health, Boston, MA., USA
Stephen Resch
Harvard Center for Health Decision Sciences, Harvard T.H. Chan School of Public Health, Boston, MA. USA
Katherine E. A. Semrau
Ariadne Labs, Brigham & Women's Hospital and Harvard T.H. Chan School of Public Health, Boston, MA., USA
Department of Medicine, Harvard Medical School, Boston, MA. USA
Division of Global Health Equity, Brigham & Women's Hospital, Boston, MA. USA

Word Count: 3157

For peer review only - http://bmjopen.bmj.com/site/about/guidelines.xhtml

Abstract:

Objectives

Despite global concern over the quality of maternal care, little is known about the time requirements to complete essential birth practices. Using three micro-costing data collection methods within the BetterBirth trial, we aimed to assess the birth attendant time use and the specific time requirements to incorporate the WHO Safe Childbirth Checklist into clinical practice.

Setting

We collected detailed data on birth attendant time-use in the BetterBirth trial in Uttar-Pradesh, India. It was a matched-pair, cluster-randomized, controlled trial to test whether the peer-coaching-based implementation of the WHO Safe Childbirth Checklist was effective in improving the quality of facility-based childbirth care.

Participants

We collected measurements of time-to-completion for 18 essential birth practices from July 2016 through October 2016 across 10 facilities in 5 districts. An anonymous survey asked about the impact of the WHO Safe Childbirth Checklist on birth attendants at every intervention facility (n=15) in the Lucknow hub. Additionally, data collectors visited facilities to conduct a census of patients and birth attendants across 20 facilities in 7 districts between June 2016 to November 2016.

Primary and secondary outcome measures

The primary outcome measure of this study is the percent of staff time required to complete the essential birth practices included in the WHO Safe Childbirth Checklist.

Results

When birth attendants were timed, we found practices (such as handwashing, use of neonatal bag mask, and skin-to-skin initiation) were completed rapidly (18 seconds – 2 minutes). As the patient load increased, time dedicated to clinical care increased but remained low relative to administrative and downtime.

Conclusions

On average, WHO Safe Childbirth Checklist clinical care accounted for less than 7% of birth attendant time-use per hour. However, questions remain regarding the performance quality of practices and how to accurately capture and interpret idle and break time.

Trial registration

NCT02148952

For peer review only - http://bmjopen.bmj.com/site/about/guidelines.xhtml

Strengths and Limitations

Strengths

- Few previous studies include micro-costing data collection to estimate the time-requirements of the proposed policy intervention.
- The combination of three distinct time-use capture methods creates a rich understanding of how birth attendants use their time and how long specific tasks take to complete

Limitations

- Both stop-watch and birth attendant reported time-to-complete individual evidence-based practices were shorter than expected *a priori*. Self-reported time-to-complete individual evidence-based practices were longer on average than stop-watched derived estimates.
- Even during times with high patient volume, administrative and downtime were the prevailing time-use categories of birth attendants. We are not however, able to distinguish between true downtime and watchful waiting in an active clinical care setting based on our methods

For peer review only

INTRODUCTION

Remarkable achievements have been made in the reduction of maternal and neonatal mortality globally.[1,2] One of the primary achievements of the Millennium Development Goals Era was increased rate of facility-based childbirth.[3] However, evidence suggests increased coverage of services does not necessarily lead to mortality and morbidity reductions.[4,5] A large portion of stillbirths and maternal and neonatal deaths remain preventable with timely, high-quality care.[6–8] As low- and middle-income countries (LMICs) continue to expand access to services, ensuring patients receive high-quality, evidence-based clinical care is essential for continued progress in population health.[9]

The use of evidence-based care in labor and delivery facilities remains low.[10,11] Even when women reach a facility in a timely manner, without adequate and appropriate treatment, preventable deaths occur.[12] Many interventions seek to improve the quality of care in LMIC health systems by increasing the number of essential birth practices performed for each laboring mother.[9] The World Health Organization's Safe Childbirth Checklist (Checklist) is one such effort.[13] The Checklist is a clinical care aid that synthesizes and prioritizes evidence-based essential birth practices (practices) from admission to discharge in order to increase the number of practices—like handwashing, checking the mother for bleeding, or discussing family planning—performed by birth attendants (BAs) at the point of care. Defining essential practices and creating mechanisms like the Checklist for clinical staff to consistently implement those practices has been successful across a diverse set of clinical contexts in both high- and low-income settings.[14–16]

For peer review only

For peer review only - http://bmjopen.bmj.com/site/about/guidelines.xhtml

One complicating factor in quality improvement efforts targeting labor and delivery wards specifically is staff time availability. Across health systems, BAs often report feeling overwhelmed and busy.[17] Additionally, staffing shortages are a known barrier to timely, high-quality clinical care.[18] With any quality-improvement intervention, clinical care may increase staffing time demands or replace existing low-value activities. The implementation of quality-improvement interventions requires understanding existing staff time capacity at baseline and how staff time-use changes post-implementation.

To study the effect of the Checklist on birth attendants adherence to evidence-based practices, as well as maternal and perinatal outcomes, we conducted a matched-pair, cluster-randomized, controlled trial of a coaching-based implementation of the Checklist in Uttar-Pradesh, India. We specifically tested the effect of the intervention on a composite outcome of perinatal mortality, maternal mortality, or maternal severe complication within 7 days of giving birth.[19,20] While the BetterBirth trial increased adherence to practices, it did not have an effect on morbidity or mortality.

As part of the BetterBirth trial, we conducted data collection to measure the time-demands of the Checklist practices, with the primary intent of informing a cost-effectiveness analysis (CEA) of the BetterBirth trial. When the main outcome of the BetterBirth trial was a null effect on maternal and neonatal mortality and morbidity, the CEA was rendered irrelevant. However, concerns remained about the possibility that the Checklist introduced a significant time burden on BAs. Prior to the implementation of the BetterBirth trial, BAs often reported feeling

For peer review only - http://bmjopen.bmj.com/site/about/guidelines.xhtml

overwhelmed and busy. As a result, we used the collected data to answer the following questions:

(1) What is the time-burden of the practices included in the Checklist?

(2) Do BAs perceive the Checklist as a significant stress- or time-burden?

(3) How does BA time-use change as their patient load increases?

METHODS

Study setting

The BetterBirth trial was a peer-coaching-based implementation of the Checklist in Uttar Pradesh, India. The matched-pair, cluster-randomized, controlled trial randomized the BetterBirth trial across 120 facilities (60 control, 60 treatment) with a study population of women and their newborns, the birth attendants (BAs) providing care, and the facility and district-level leadership. Study facilities included primary health centers, community health centers, and first referral units (which were to have cesarean section capacity); facilities had more than 1,000 deliveries per year and minimum of four labor and delivery staff. The study protocol and results have been published and include further details on the study population, design, and methods used to test the primary outcome of interest, maternal and perinatal mortality and morbidity outcomes.[19,20]

Time-use data collection

We conducted three time-use data collection methods to triangulate the time-burden of the Checklist practices within the broader time-demands on BAs. Data collectors (N=16) were junior nurses who received training and supportive supervision for data quality assurance across all

For peer review only

three data collection methods (each described in more detail below).We captured 18 specific Checklist practices (Appendix Table A1) as well as non-Checklist clinical care, administrative duties, and break/downtime. Although the intention was to distinguish between a scheduled break and non-scheduled downtime, efforts to delineate between these two activities by data collectors was difficult in practice. For the purposes of this paper, 'downtime' refers to a mix of scheduled breaks as well as idle time for other reasons, such as no patients or watchful waiting during clinical care.

The time-demand of Checklist practices

We collected measurements of Checklist practice time-to-completion for 18 practices over a four-month period from July 2016 through October 2016 across 10 facilities in 5 districts. Data collectors visited each facility 2-3 times per week, for 8 hours shifts between 7am-3pm or 11am-7pm. If available, a second data collector or a supervisor performed data quality assurance activities. Time-to-complete tasks were assessed by the data collectors with stopwatches, recorded on paper (Appendix Table A2), and transferred to an Excel spreadsheet. The time measurements were used to estimate the time required to complete each Checklist practice.

Perceived time-demand of the Checklist by BAs

We also surveyed BAs on their time-burden perceptions. The anonymous survey asked general questions about the impact of the Checklist on the daily routines and workloads of BAs at every intervention facility (n=15) in the Lucknow hub (the cost-effectiveness data collection survey region with 30 total facilities). The survey also asked respondents to rank the top three most time-consuming items on the Checklist and estimate the time required to complete those tasks.

For peer review only - http://bmjopen.bmj.com/site/about/guidelines.xhtml

The specific time estimates for Checklist practices were used to supplement and compare with the stopwatch time measurements.

BA time-use in the labor and delivery ward

How providers use their time depends on both the patient demand and the number of BAs on duty. To estimate the patient demand and health care labor supply, data collectors visited facilities to conduct a census of patients and BAs every 2 hours, recording the results on a paper form (Appendix Table A4-5). Observations were taken across 20 facilities in 7 districts from June 2016 to November 2016. This data was used to calculate the average number of patients per BA at given facilities and times of day.

In addition to the census, we also observed BAs conducting regular care (a work sampling approach) to capture the proportion of time spent on various types of clinical and non-clinical work.[21] A data collector visited a facility and, for each hour observed, recorded the type of activity the BA was engaged in at pre-specified 2-minute intervals on a paper form (Appendix Table A3-4). For example, if at 11:00 a.m. the BA was using a neonatal bag and mask, the data collector recorded that activity. At 11:02 a.m. the data collector would again record what the BA was doing; in some cases, she might still be using a neonatal bag and mask, while in other cases, there may be a new activity listed such as non-Checklist direct patient care. This type of data provides estimates of proportional time spent on various activities but does not directly estimate the time required for specific tasks.

For peer review only - http://bmjopen.bmj.com/site/about/guidelines.xhtml

If there were at least two BAs on duty at the same time, observations alternated between two BAs. For example, the 11:00 AM observation would pertain to BA1 while the 11:02 AM observation would pertain to BA2, alternating back and forth throughout the hour. If only one BA was available for observation, an observation was taken every 2 minutes for their work. We calculate the proportion of each BA's time spent in different general activity categories to estimate the overall time-use in given facility-hours (Appendix Table A5 maps specific activities to general categories).

Ethics approval

The study protocol was approved by the ethics committees of: Community Empowerment Lab (Ref no: 2014006), Jawaharlal Nehru Medical College (Ref no: MDC/IECHSR/2015–16/A-53), Harvard T.H. Chan School of Public Health (Protocol 21 975–102), Population Services International (Protocol ID: 47·2012), WHO (Protocol ID: RPC 501) and Indian Council of Medical Research. The protocol was reviewed and re-approved on an annual basis. We obtained consent from each facility's leadership for trial participation and data collection on eligible mothers from facility registers. Birth attendants and facility staff verbally agreed to participate prior to trial initiation. Independent observers obtained written consent from women or their surrogates and verbal consent from birth attendants prior to observation.

Public involvement in research

Patient and provider representatives worked with us to refine the Checklist when it was originally designed in 2009. The BetterBirth trial study research question and design did not have direct patient involvement, but did have a scientific advisory committee that included

For peer review only

clinicians, researchers, government officials who work in the same area. We did receive and modified the dissemination plan based on feedback from providers and government partners for each participating facility/district. Further, we published a report for wider dissemination and audience found at: betterbirth.ariadnelabs.org

RESULTS

The time-demand of Checklist practices

Across all Checklist practices, a total of 1,559 practices were directly timed from 35 unique birth attendants (BAs) across 10 facilities during clinical care. Handwashing (N=419) and the administration of medication (N=208) were the most frequently observed direct measurements, while referrals (N=21) and the assessment of the baby's breathing (N=9) had the fewest recorded observations. Directly measured task-times revealed a pattern of rapid time-to-complete practices on the Checklist. When Checklist practices were directly measured using stopwatches, the average time-to-complete the task ranged from 127 seconds (a referral) to 18 seconds (weighing the baby). Tasks like breastfeeding initiation and discussing family planning that require conversations and (potentially) complex patient-BA interactions both took less than 1 minute on average (black dots on Figure 1 and Appendix Table A6). Over 70% (N=12 out of 18 practices) of the average time-to-complete measurements were less than one minute.

Perceived time-demand of the Checklist by BAs

Across 15 facilities, there were 83 total respondents to the survey. The majority of BAs responded that the Checklist made their jobs easier (96%; N=80). When BAs were asked if the

For peer review only - http://bmjopen.bmj.com/site/about/guidelines.xhtml

Checklist took away from non-Checklist activities, only 17% of responders felt other clinical duties were rushed (N=11) or their workday was prolonged (N=3).

We directly asked BAs (n=83) to rank the three most time-consuming Checklist practices and estimate the time required to complete those three tasks. Discussing family planning was the most frequently reported time-consuming activity (ranked #1 by 49% of BAs (Appendix Table A7)). All tasks were estimated by BAs to take less than 5:07 minutes on average. The self-reported task-times were longer than the direct measurements, particularly in discussion-based practices like explaining danger signs and discussion family planning (Figure 1).

BA time-use in the labor and delivery ward

BA time-use incorporates data from the work sampling time-use data collection and the facility BA and patient census. In total, 610 2-hour facility periods were recorded for the patient census. Within the hours of data collection (7am-7pm), we found relatively constant median patient-load at 1.4 patients-per-BA with large variability in the potential patient-load for any given facility-hour (0 to 8 patients-per-BA observed range) (Appendix Figure A1).

Clinical care (both non-Checklist and Checklist) was 21% of the average facility-staff hour. As patients-per-BA increased, so did clinical care and administrative duties. When there were no patients, BAs spent the majority of their time in downtime (80% of time) or conducting administrative tasks (15% of time). Once the patient-load increased to 1-2 patients-per-BA, BA time-use shifted towards clinical care (23% of BA time) as well as administrative tasks (26% up from 15% with no patients). At 3 or more patients-per-BA, the Checklist accounted for 7% of

For peer review only - http://bmjopen.bmj.com/site/about/guidelines.xhtml

BA time (out of a total 24% of the hour spent on clinical care). However, even at high patient-loads (3+), the most common time-use, on average, was still recorded as downtime (40% in downtime compared to 24% in clinical care; Figure 2).

However, the average BA downtime is a misleading statistic. When the full distribution of BA downtime by facility-BA-hour are graphed, there is clear heterogeneity in the distribution that is not captured by summary measures like the mean or median percent of the hour spent in downtime. Particularly for the 1-2 patient categorization, there is a clear bi-modal trend with BAs spending the majority of staff-hours either completely in downtime or without any downtime. Similarly, for the 0 and 3+ patient categories, the distributions are highly skewed to all downtime (0 patients) and no downtime (3+ patients). Taken across all these categories, summary statistics mask the extreme downtime dichotomy experienced in practice by BAs (Figure 3, Appendix Figure A2).

DISCUSSION

The time-demand on BAs is an important piece of the maternal and newborn quality-of-care puzzle. Quality improvement efforts inherently require staff time to shift away from existing time uses and towards evidence-based practices such as those included in the Checklist. Using three different data collection efforts, we found that the Checklist practices were not an undue time burden on BAs. However, based on our data, we are not confident that practices were performed at sufficient quality. Further, our results show a high proportion of ambiguously measured downtime and potential issues with the face validity in the time-to-complete practice assessment. Concerns about the quality of care provided are consistent with the overall

For peer review only - http://bmjopen.bmj.com/site/about/guidelines.xhtml

BetterBirth trial findings—treatment facilities did not have reduced mortality and morbidity after the Checklist was implemented.

Several of the time-to-complete practice measures seemed implausibly fast to be of sufficient quality. In particular, tasks like initiation of breastfeeding, initiation of skin-to-skin contact, discussion or family planning, and referrals likely require more time in expectation than is currently being allocated based on our study results. Referrals, which were the most time-consuming task overall, were still completed within 2 minutes. Given the difficulty of breastfeeding initiation,[22,23] it is unlikely that a mean task-time of 24 seconds (SE = 16 seconds) accurately captures the true time required to successfully initiate breastfeeding. In other cases, the timing seems plausible. For example, the CDC recommends handwashing for 15-20 seconds.[24] In our sample, handwashing took an average of 29 seconds. Similarly, although the self-reported sample of task-time estimates is skewed towards tasks that the BAs perceived as relatively more burdensome, the self-reported time-to-complete tasks remained lower than expected *a priori*. These results are similarly indicative that time-to-complete Checklist-related practices are too low to have been consistently performed at high-quality. Further research is needed to estimate minimum time requirements for the performance of practices at high quality and how the Checklist, when implemented at high-quality, impacts the staffing needs of a facility. One potential downside of a checklist-based intervention is a desire to get through the items as quickly as possible rather than at the pace required to perform each task at high-quality. Further quality approaches may also consider how to incorporate incentives for not just completing a checklist but reaching quantifiable quality benchmarks for the checklist items.

For peer review only - http://bmjopen.bmj.com/site/about/guidelines.xhtml

Although individual practices took less than 2 minutes to complete on average and overall less than 5 minutes for the full practice list, it is still possible that the workload of clinical care (and/or administrative tasks) before the introduction of the Checklist was sufficiently demanding that BAs did not have the slack to take on any incremental tasks newly introduced with the Checklist. Across all our data collection methods, however, high-quality clinical care was not the major time-use of BAs in the BetterBirth study population. One of the main open questions from our time-use data collection is how to understand and estimate the time-constraints faced by labor and delivery ward BAs. The nature of labor and delivery ward care requires long periods of waiting followed by high-stress, high-demand moments of clinical care. Could moments of inactivity actually be high-stress, high-alert contexts compared to times when the BA is truly on break? How should we differentiate between breaks that are necessary versus time that could be reallocated towards high-quality clinical care? How would the percent of time spent conducting clinical care change if quality of care improved? Our data highlights the importance and difficulty of estimating supply-side constraints in the highly unpredictable context of labor and delivery wards. In the future, it will be important to continue to estimate how quality improvement interventions impact the time-use of providers including work to parse out time which appears to be free, but in reality may be a version of alert waiting.

Ensuring quality care at facilities not only requires thoughtful clinical care practices, it also requires staffing strategies.[25–29] Our data collection efforts add empiric evidence on how BAs in Uttar Pradesh, India use their time across both clinical and non-clinical care under varying levels of patient demand. In future implementations of the Checklist, our data on the time-to-complete clinical tasks as well as the time-use of BAs can serve as both a model of how to

For peer review only - http://bmjopen.bmj.com/site/about/guidelines.xhtml

collect data and as a baseline for potential data collection improvements that could address lingering questions raised in this paper.

There are several limitations in our methods and data collection. Although we began with separate categories for breaks and downtime, this distinction was not clear during data collection. We cannot reliably distinguish true breaks from inactive alert waiting. Practices were meant to be timed from start to finish, pausing for breaks. For instance, if a family planning discussion began but was interrupted by breastfeeding initiation, the stopwatch should have been stopped and restarted when the family planning discussion restarted to capture the overall time required for that practice. Given the consistently short task-time estimates, this may not have occurred. In our survey of BAs, our sample size is relatively small (N=83), the responses may not generalize to the broader BA population in our study and Uttar Pradesh more broadly. Instead of asking the BAs to estimate the task-time for all 18 practices, we only asked for the top three in an effort to keep the survey short. However, it limits our self-reported task-times to only those activities that BAs considered especially time consuming, biasing the self-reported results upwards.

There are often calls for measurable indicators of health care quality. In the recent Lancet Global Health Commission on High Quality Health Systems, many of the available quality-metrics rely on the proportion or number of evidence-based practices performed.[9] Although completion of tasks is important, our evidence suggests simply performing evidence-based care does not itself ensure quality. This outcome mirrors the message that coverage of services does not equate to quality. When future quality-improvement and evidence-based care interventions are implemented, it will remain important to understand how the intervention fits within the broader

For peer review only - http://bmjopen.bmj.com/site/about/guidelines.xhtml
For peer review only

responsibilities and time demands of BAs. Quality care requires essential care is completed at a satisfactory level beyond simple completion of tasks.

ACKNOWLEDGMENTS
We recognize the Governments of India and Uttar Pradesh for collaboration and support to conduct this trial in public health facilities. We thank the facility staff, women and newborns for their participation in the study. We are indebted to Sriya Srikrishnan, Ezinne Eze-Ajoku, Bharath Kumar, Krishna Kumar, Narender Sharma, and all the data collectors on the facility-based data associate team. We are grateful to the members of the trials' Scientific Advisory Committee who contributed crucial guidance to the development of this study protocol.

CONTRIBUTORSHIP STATEMENT
KEAS, SR, and KTL conceived of the study. KTL conducted the analysis. VPS and TK supervised field operations and reviewed survey instruments. KTL, AK, KEAS, and LB wrote the manuscript. AK provided extensive edits and manuscript support. LB, DET, MMD, and AJ provided operational support for the study. MR provided clinical inputs for the study. All authors (KEAS, LB, DET, VPS, MMD, AJ, MR, TK, AK, SR, and KTL) reviewed the manuscript and provided edits.

COMPETING INTERESTS
The authors have no competing interests to declare.

FUNDING
Bill and Melinda Gates Foundation (OPP1017378)

DATA SHARING
Data are available in a public, open access repository. Data are available on the Harvard Dataverse Platform under the BetterBirth Dataverse website. This can be found at: https://dataverse.harvard.edu/dataverse/BetterBirthData

For peer review only - http://bmjopen.bmj.com/site/about/guidelines.xhtml

REFERENCES

1. Hogan DR, Stevens GA, Hosseinpoor AR, et al. Monitoring universal health coverage within the Sustainable Development Goals: development and baseline data for an index of essential health services. *Lancet Glob Health* 2018 Feb;6(2):e152-e168. doi: 10.1016/S2214-109X(17)30472-2.

2. Lozano R, Wang H, Foreman KJ, et al. Progress towards Millennium Development Goals 4 and 5 on maternal and child mortality: an updated systematic analysis. *The Lancet*. 2011 Sep 24;378(9797):1139-65. doi: 10.1016/S0140-6736(11)61337-8.

3. United Nations Millennium Development Goals [Internet]. [access date 2018 Sep]. Available from: http://www.un.org/millenniumgoals/maternal.shtml

4. Ng M, Misra A, Diwan V, et al. An assessment of the impact of the JSY cash transfer program on maternal mortality reduction in Madhya Pradesh, India. *Glob Health Action*. 2014 Dec 3;7:24939. doi: 10.3402/gha.v7.24939.

5. Okeke, Edward N. and Amalavoyal V. Chari, Can Institutional Deliveries Reduce Newborn Mortality? Evidence from Rwanda. Santa Monica, CA: *RAND Corporation*, 2015. https://www.rand.org/pubs/working_papers/WR1072.html

6. Ronsmans C, Scott S, Qomariyah SN, et al. Professional assistance during birth and maternal mortality in two Indonesian districts. *Bull World Health Organ*. 2009 Jun;87(6):416-23. doi: 10.2471/blt.08.051581.

7. Lawn JE, Blencowe H, Pattinson R, et al. Stillbirths: Where? When? Why? How to make the data count? *The Lancet*. 2011 Apr 23;377(9775):1448-63. doi: 10.1016/S0140-6736(10)62187-3.

8. Kruk ME, Gage AD, Joseph NT, et al. Mortality due to low-quality health systems in the universal health coverage era: a systematic analysis of amenable deaths in 137 countries. *The Lancet*. 2018 Nov 17;392(10160):2203-2212. doi: 10.1016/S0140-6736(18)31668-4.

9. Kruk ME, Gage AD, Arsenault C, et al. High-quality health systems in the Sustainable Development Goals era: time for a revolution. *Lancet Glob Health*. 2018 Nov;6(11):e1196-e1252. doi: 10.1016/S2214-109X(18)30386-3.

10. Bhutta ZA, Das JK, Bahl R, et al. Can available interventions end preventable deaths in mothers, newborn babies, and stillbirths, and at what cost? *The Lancet*. 2014 Jul 26;384(9940):347-70. doi: 10.1016/S0140-6736(14)60792-3.

11. Leslie HH, Sun Z, Kruk ME. Association between infrastructure and observed quality of care in 4 healthcare services: A cross-sectional study of 4,300 facilities in 8 countries. *PLoS Med.* 2017 Dec 12;14(12):e1002464. doi: 10.1371/journal.pmed.1002464.

For peer review only - http://bmjopen.bmj.com/site/about/guidelines.xhtml

12. Thaddeus S, Maine D. Too far to walk: Maternal mortality in context. Soc Sci Med. 1994 Apr 1;38(8):1091–110.

13. WHO | WHO Safe Childbirth Checklist [Internet]. WHO. [access date 2018 May 3]. Available from: http://www.who.int/patientsafety/implementation/checklists/childbirth/en/

14. Perry WRG, Nejad SB, Tuomisto K, et al. Implementing the WHO Safe Childbirth Checklist: lessons from a global collaboration. *BMJ Glob Health*. 2017 Aug 19;2(3):e000241. doi: 10.1136/bmjgh-2016-000241.

15. Haynes AB, Weiser TG, Berry WR, et al. A Surgical Safety Checklist to Reduce Morbidity and Mortality in a Global Population. N Engl J Med. 2009 Jan 29;360(5):491-9. doi: 10.1056/NEJMsa0810119.

16. Arriaga AF, Bader AM, Wong JM, et al. Simulation-Based Trial of Surgical-Crisis Checklists. *N Engl J Med.* 2013 Jan 17;368(3):246-53. doi: 10.1056/NEJMsa1204720.

17. Munabi-Babigumira S, Glenton C, Lewin S, et al. Factors that influence the provision of intrapartum and postnatal care by skilled birth attendants in low- and middle-income countries: a qualitative evidence synthesis. *Cochrane Database Syst Rev.* 2017 Nov 17;11(11):CD011558. doi: 10.1002/14651858.CD011558.pub2.

18. Knight HE, Self A, Kennedy SH. Why are women dying when they reach hospital on time? A systematic review of the "third delay." *PLoS One.* 2013 May 21;8(5):e63846. doi: 10.1371/journal.pone.0063846.

19. Semrau KEA, Hirschhorn LR, Marx Delaney M, et al. Outcomes of a Coaching-Based WHO Safe Childbirth Checklist Program in India. *N Engl J Med.* 2017 Dec 14;377(24):2313-2324. doi: 10.1056/NEJMoa1701075.

20. Semrau KEA, Hirschhorn LR, Kodkany B, et al. Effectiveness of the WHO Safe Childbirth Checklist program in reducing severe maternal, fetal, and newborn harm in Uttar Pradesh, India: study protocol for a matched-pair, cluster-randomized controlled trial. *Trials.* 2016 Dec 7;17(1):576. doi: 10.1186/s13063-016-1673-x.

21. Tsai W-H. A technical note on using work sampling to estimate the effort on activities under activity-based costing. *Int J Prod Econ.* 1996 May 1;43(1):11–6. https://doi.org/10.1016/0925-5273(95)00189-1.

22. Wallwiener S, Müller M, Doster A, et al. Predictors of impaired breastfeeding initiation and maintenance in a diverse sample: what is important? *Arch Gynecol Obstet.* 2016 Sep;294(3):455-66. doi: 10.1007/s00404-015-3994-5.

23. Colin WB, Scott JA. Breastfeeding: reasons for starting, reasons for stopping and problems along the way. *Breastfeed Rev.* 2002 Jul;10(2):13-9

For peer review only - http://bmjopen.bmj.com/site/about/guidelines.xhtml

24. Centers for Disease Control and Prevention. Guideline for Hand Hygiene in Health-Care Settings: Recommendations of the Healthcare Infection Control Practices Advisory Committee and the HICPAC/SHEA/APIC/IDSA Hand Hygiene Task Force. MMWR 2002;51

25. Austin A, Langer A, Salam RA et al. Approaches to improve the quality of maternal and newborn health care: an overview of the evidence. *Reprod Health*. 2014 Sep 4;11 Suppl 2(Suppl 2):S1. doi: 10.1186/1742-4755-11-S2-S1.

26. Bryant AS, Worjoloh A, Caughey AB, et al. Racial/ethnic disparities in obstetric outcomes and care: prevalence and determinants. *Am J Obstet Gynecol.* 2010 Apr;202(4):335-43. doi: 10.1016/j.ajog.2009.10.864.

27. Mann S, Hollier LM, McKay K, et al. What We Can Do about Maternal Mortality — And How to Do It Quickly. *N Engl J Med.* 2018 Nov 1;379(18):1689-1691. doi: 10.1056/NEJMp1810649.

28. Koblinsky M, Moyer CA, Calvert C, et al. Quality maternity care for every woman, everywhere: a call to action. *The Lancet*. 2016 Nov 5;388(10057):2307-2320. doi: 10.1016/S0140-6736(16)31333-2.

29. Bustreo F, Say L, Koblinsky M, et al. Ending preventable maternal deaths: the time is now. *Lancet Glob Health.* 2013 Oct;1(4):e176-7. doi: 10.1016/S2214-109X(13)70059-7.

FIGURE 1
Title: Time-to-complete specific Checklist related tasks*
Legend: Tasks with 2 or fewer observations have been excluded from this graph but are included in Appendix Table A6

FIGURE 2
Title: Birth attendant tasks stratified by patient-load per provider
Legend: none

FIGURE 3
Title: Percent of facility-staff hours recorded as downtime by patient-load per birth attendant
Legend: none

For peer review only - http://bmjopen.bmj.com/site/about/guidelines.xhtml

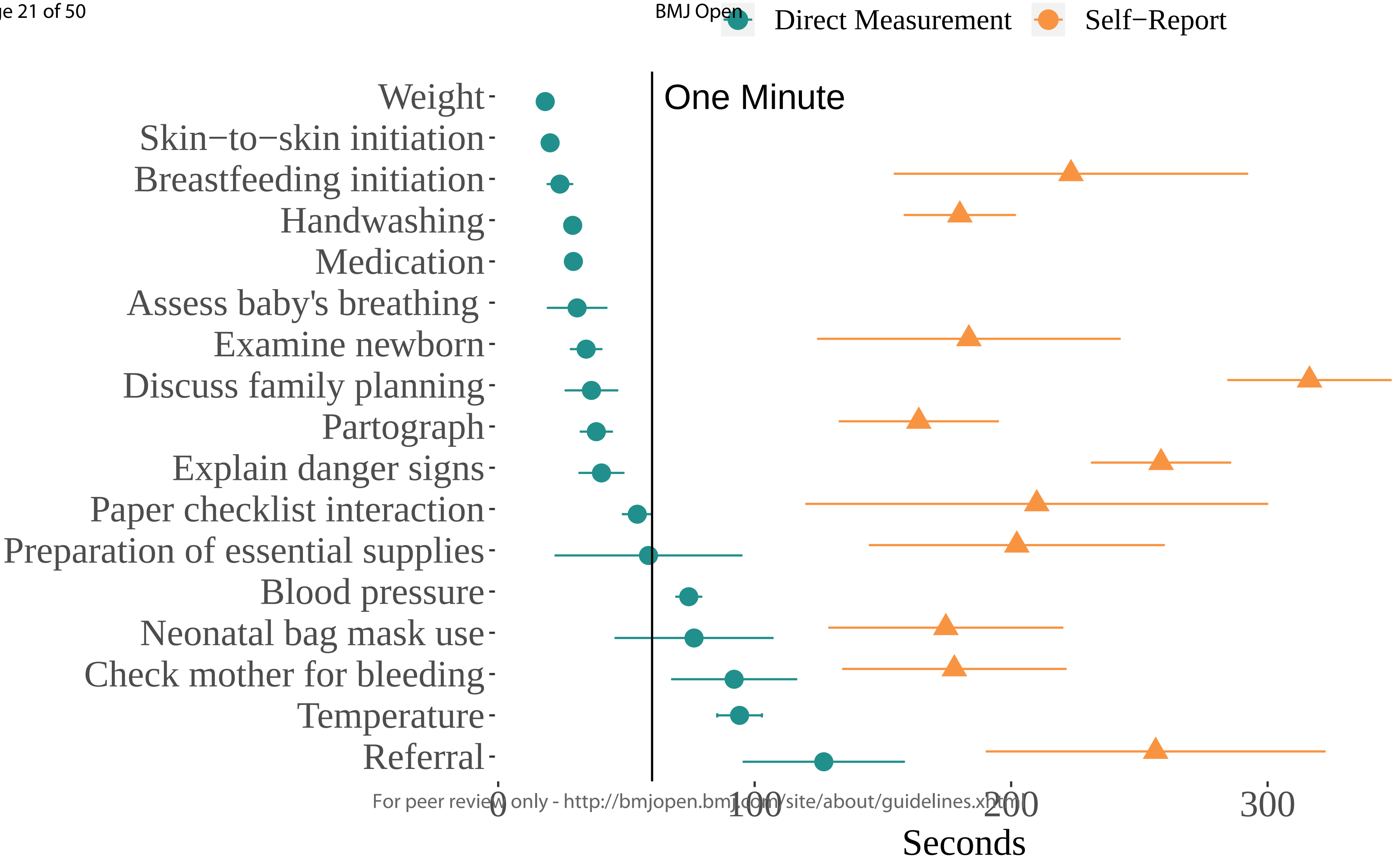
Direct Measurement
Self−Report
One Minute
Weight
Skin−to−skin initiation
Breastfeeding initiation
Handwashing
Medication
Assess baby's breathing
Examine newborn
Discuss family planning
Partograph
Explain danger signs
Paper checklist interaction
Preparation of essential supplies
Blood pressure
Neonatal bag mask use
Check mother for bleeding
Temperature
Referral
0
100
200
300
Seconds

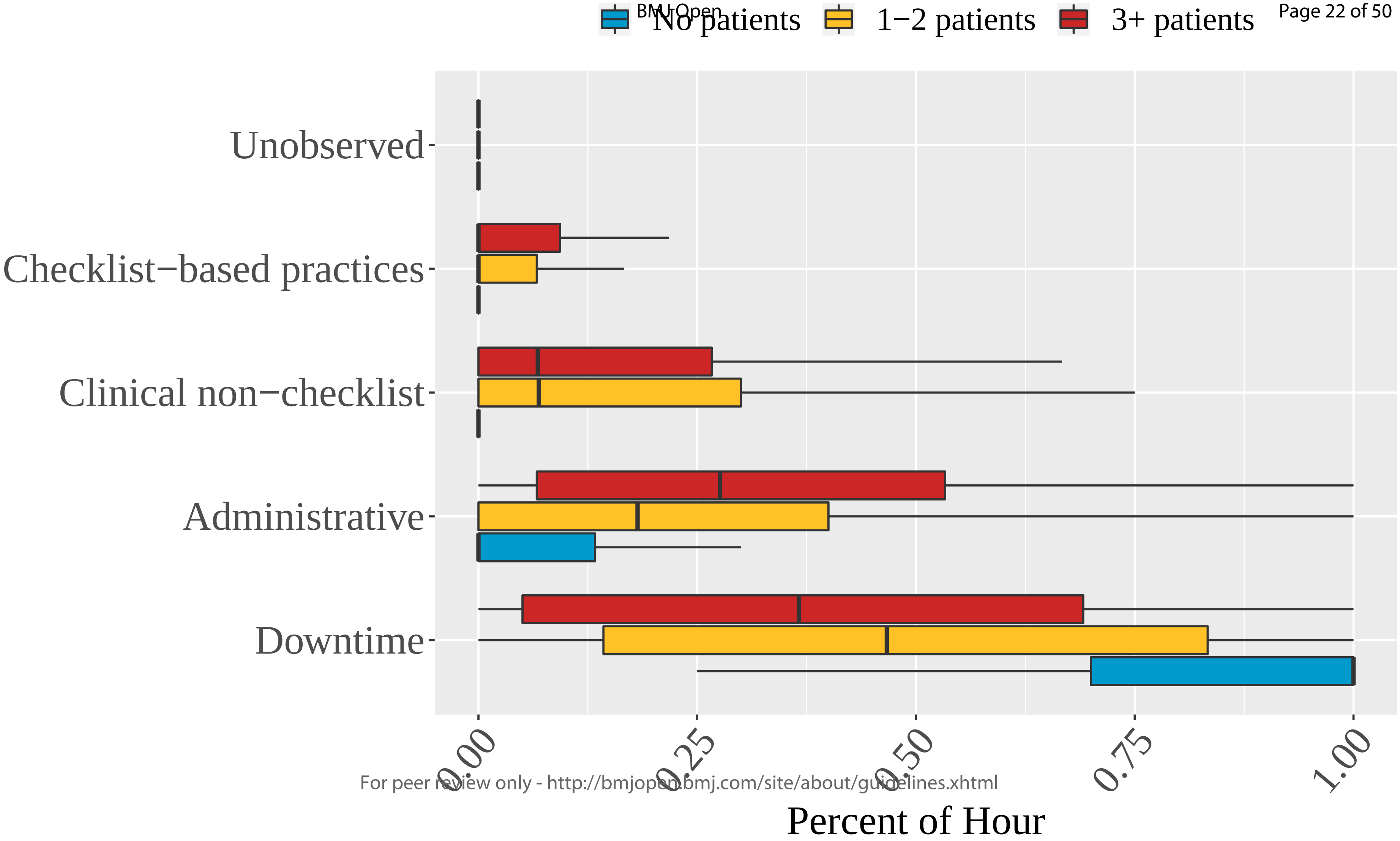

For peer review only - http://bmjopen.bmj.com/site/about/guidelines.xhtml

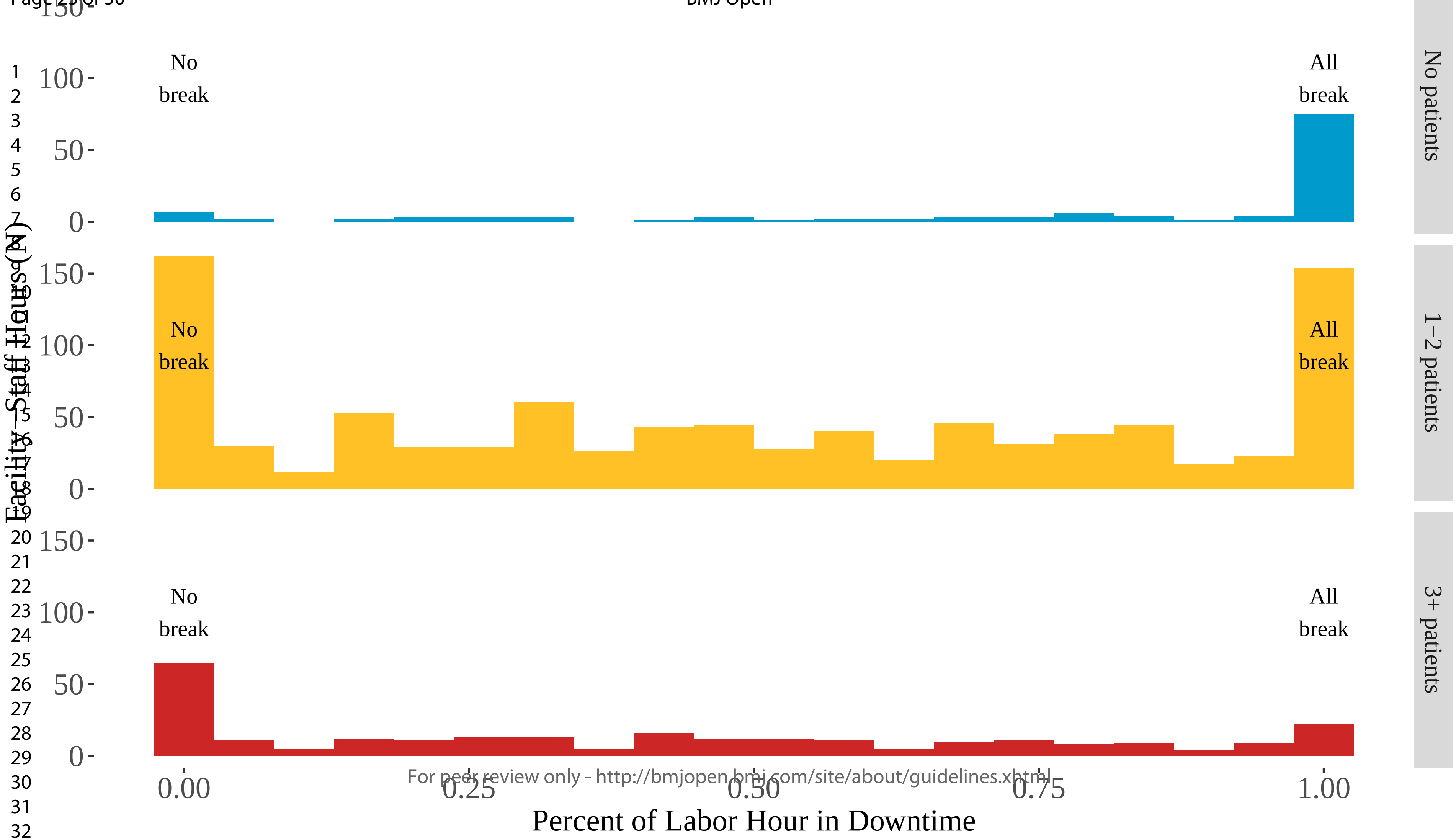
Facility−Staff Hours (N)
150
100
50
0
No
break
All
break
No patients
1−2 patients
3+ patients
0.00
0.25
0.50
0.75
1.00
Percent of Labor Hour in Downtime

Supplementary Appendix

Labor in labor and delivery wards:
Evidence on provider time-use from the BetterBirth Trial

Appendix Table A1: Activities measured by data collection method

Master (18)	Work Sampling (16)	Time Motion (17)	Time Use (14)
Temperature	Temperature	Temperature	Temperature
Blood pressure	Blood pressure	Blood Pressure	N/A
Partograph	Partograph	Partograph	Partograph
Paper checklist interaction	Checklist/ poster	Paper checklist interaction	Checklist/ poster
Medication	Medication	Admin. Antibiotics/ Admin. Vaccines	Medication
Handwashing	Handwash gloves or alcohol rub	Handwashing	Handwash gloves or alcohol rub
Preparation of essential supplies	Prep of EBS	Prep of essential supplies	Prep of EBS
Neonatal bag mask	Use neonatal bag mask	Neonatal bag	Use neonatal bag mask
Referral	Referring a patient	Referral	Referring a patient
Check mother for bleeding	Check Mother for bleeding	Check mother for bleeding	Check mother for bleeding
Examine newborn	Examination of Newborn (BA)<br>Examination of Newborn (ASHA)	Examine newborn<br>Examine newborn for danger signs	Examination of newborn<br>N/A
Skin-to-skin initiation	Initiation of skin-to-skin	Init. of skin-to-skin	N/A
Discuss family planning	Discussing family planning<br>Group discussion	Discuss family planning<br>Discuss family planning (Group)	Discussing family planning<br>N/A
Explain danger signs	Explaining danger signs	Explain danger signs	Explaining danger signs
Breastfeeding initiation	Initiation of breastfeeding	Init. of breastfeeding	Initiation of breastfeeding
Confirm vaccination	Confirmation of vaccination	N/A	Confirmation of vaccination
Weight	N/A	Weight	N/A
Check baby's breathing	N/A	Assess Baby's Breathing	N/A

For peer review only - http://bmjopen.bmj.com/site/about/guidelines.xhtml

Appendix Table A2: Time Motion Observation Tool
(see next page for start of PDF)

For peer review only

For peer review only - http://bmjopen.bmj.com/site/about/guidelines.xhtml

Time Motion Observation Tool

A	Facility Code	
B	Date of Observation (DD/MMM/YYYY)	____/____/____
C	Health Care Worker Unique ID	
D	Health Care Worker Cadre	☐ Doctor ☐ L.H.V ☐ A.N.M ☐ Staff Nurse ☐ Other
E	Years of experience as a Health Care Worker	________Years _______Months
F	Years of experience as a Health Care Worker at this health facility	________Years _______Months
G	Did Health Care Worker consent to Observation	___Yes ___No
H	Notes about Time Motion observations:	
I	Tool ID Code	
J	FADA Employee ID	
K	FADA Role	☐ First Observer ☐ DQA Observer
L	FADA Start Time	______:______
M	FADA End Time	______:______

	Patient Yearly Number___________<br><br>From which register did you get the yearly number?<br>❑ Delivery<br>❑ Referred Out<br>❑ Admission				Patient Yearly Number _____________<br><br>From which register did you get the yearly number?<br>❑ Delivery<br>❑ Referred Out<br>❑ Admission				Patient Yearly Number __________<br><br>From which register did you get the yearly number?<br>❑ Delivery<br>❑ Referred Out<br>❑ Admission				General Activity (cannot be assigned to specific patient)
	PP 1	PP 2	PP 3	PP 4	PP 1	PP 2	PP 3	PP 4	PP 1	PP 2	PP 3	PP 4	
**Maternal Temperature**													
**Blood Pressure**													
**Partograph interaction**													
**Paper checklist or poster interaction**													
**Administration of antibiotics, magnesium sulfate, oxytocin or antiretroviral**													
**Hand washing, clean gloves or alcohol rub**													
**Preparation of Essential Supplies at bedside table**													
**Use of neonatal bag and mask for baby**													
**Referring a patient**													

	Patient Yearly Number___________<br><br>From which register did you get the yearly number?<br>❑ Delivery<br>❑ Referred Out<br>❑ Admission				Patient Yearly Number _____________<br><br>From which register did you get the yearly number?<br>❑ Delivery<br>❑ Referred Out<br>❑ Admission				Patient Yearly Number __________<br><br>From which register did you get the yearly number?<br>❑ Delivery<br>❑ Referred Out<br>❑ Admission				General Activity (cannot be assigned to specific patient)
	PP 1	PP 2	PP 3	PP 4	PP 1	PP 2	PP 3	PP 4	PP 1	PP 2	PP 3	PP 4	
**Check mother for bleeding**													
**Examination of Newborn**													
Examine the Baby for Danger Signs													
Assess Baby's Breathing													
Take Baby's Temperature													
Take Baby's Weight													
Monitor the Baby in order to Take Appropriate Action for Special Care (Requires Resuscitation)													
**Initiation of skin-to-skin**													

	Patient Yearly Number___________<br><br>From which register did you get the yearly number?<br>❑ Delivery<br>❑ Referred Out<br>❑ Admission				Patient Yearly Number _____________<br><br>From which register did you get the yearly number?<br>❑ Delivery<br>❑ Referred Out<br>❑ Admission				Patient Yearly Number __________<br><br>From which register did you get the yearly number?<br>❑ Delivery<br>❑ Referred Out<br>❑ Admission				General Activity (cannot be assigned to specific patient)
	PP 1	PP 2	PP 3	PP 4	PP 1	PP 2	PP 3	PP 4	PP 1	PP 2	PP 3	PP 4	
**Discussing Family Planning**													
**Explaining Danger Signs for mother and child**													
Group Discussion (if family planning and danger signs could not be observed)													
**Initiation of breastfeeding**													
**Administration of Vaccination**													

	Patient Yearly Number___________<br><br>From which register did you get the yearly number?<br>❑ Delivery<br>❑ Referred Out<br>❑ Admission				Patient Yearly Number _____________<br><br>From which register did you get the yearly number?<br>❑ Delivery<br>❑ Referred Out<br>❑ Admission				Patient Yearly Number __________<br><br>From which register did you get the yearly number?<br>❑ Delivery<br>❑ Referred Out<br>❑ Admission				General Activity (cannot be assigned to specific patient)
	PP 1	PP 2	PP 3	PP 4	PP 1	PP 2	PP 3	PP 4	PP 1	PP 2	PP 3	PP 4	
**Maternal Temperature**													
**Blood Pressure**													
**Partograph interaction**													
**Paper checklist or poster interaction**													
**Administration of antibiotics, magnesium sulfate, oxytocin or antiretroviral**													
**Hand washing, clean gloves or alcohol rub**													
**Preparation of Essential Supplies at bedside table**													
**Use of neonatal bag and mask for baby**													
**Referring a patient**													

	**Patient Yearly Number___________**<br><br>**From which register did you get the yearly number?**<br>❑ **Delivery**<br>❑ **Referred Out**<br>❑ **Admission**				**Patient Yearly Number _____________**<br><br>**From which register did you get the yearly number?**<br>❑ **Delivery**<br>❑ **Referred Out**<br>❑ **Admission**				**Patient Yearly Number __________**<br><br>**From which register did you get the yearly number?**<br>❑ **Delivery**<br>❑ **Referred Out**<br>❑ **Admission**				**General Activity (cannot be assigned to specific patient)**
	PP 1	PP 2	PP 3	PP 4	PP 1	PP 2	PP 3	PP 4	PP 1	PP 2	PP 3	PP 4	
**Check mother for bleeding**													
**Examination of Newborn**													
Examine the Baby for Danger Signs													
Assess Baby's Breathing													
Take Baby's Temperature													
Take Baby's Weight													
Monitor the Baby in order to Take Appropriate Action for Special Care (Requires Resuscitation)													
**Initiation of skin-to-skin**													

	Patient Yearly Number___________<br><br>From which register did you get the yearly number?<br>❑ Delivery<br>❑ Referred Out<br>❑ Admission				Patient Yearly Number _____________<br><br>From which register did you get the yearly number?<br>❑ Delivery<br>❑ Referred Out<br>❑ Admission				Patient Yearly Number __________<br><br>From which register did you get the yearly number?<br>❑ Delivery<br>❑ Referred Out<br>❑ Admission				General Activity (cannot be assigned to specific patient)
	PP 1	PP 2	PP 3	PP 4	PP 1	PP 2	PP 3	PP 4	PP 1	PP 2	PP 3	PP 4	
**Discussing Family Planning**													
**Explaining Danger Signs for mother and child**													
Group Discussion (if family planning and danger signs could not be observed)													
**Initiation of breastfeeding**													
**Administration of Vaccination**													

Appendix Table A3: Work Sampling Census
(see next page for start of PDF)

For peer review only

BB Work Sampling Census Sheet

Cover Page

A	Facility Code	
B	Date of Observation (DD/MMM/YYYY)	______/______/______
C	Notes about Work Sampling Census Observation:	
D	FADA Employee ID	

Work Sampling Census Sheet 29April2016

BB Work Sampling Census Page

Directions: update these numbers when you begin work sampling and in the last 5 minutes of every 2 hours of observation. Record the time and update the numbers based on FADA TL observation only and not official record

Hour	Start	Start + 2	Start + 4	Start + 6	Start + 8	Start + 10				
Clock Time										
Number of Women Admitted During Previous 2 hours										
Number of Women CURRENTLY in Waiting Room										
Number of Women CURRENTLY in L&D										
Number of Women CURRENTLY in Recovery										
Number Women Discharged / Transferred / Died During Previous 2 hours										
Number of Birth Attendants CURRENTLY on Duty in L&D										
Number of Helpers CURRENTLY on Duty in L&D										

Work Sampling Census Sheet 29April2016

Appendix Table A4: Work Sampling Observation Tool
(see next page for start of PDF)

For peer review only

For peer review only - http://bmjopen.bmj.com/site/about/guidelines.xhtml

BB Work Sampling Observation Tool

A	Facility Code	
B	Date of Observation (DD/MMM/YYYY)	____/____/____
C	Health Care Worker Unique ID	
D	Health Care Worker Cadre	□ Doctor □ L.H.V □ A.N.M □ Staff Nurse □ Other
E	Years of Experience as a Health Worker	________ Years ________ Months
F	Years of Experience as a Health Worker at this health facility	
G	Did Health care Worker consent to Observation	___Yes ___No
H	Notes about Work Sampling Observation:	
I	Tool ID Code	
J	FADA Employee ID	
K	FADA Role	□ First Observer □ DQA Observer
L	FADA Start Time	______:______
M	FADA End Time	______:______

Patients Consented	
Yearly Number	From which register did you get the yearly number?

BB Work Sampling Tool 10Jun2016

For peer review only - http://bmjopen.bmj.com/site/about/guidelines.xhtml

WHO Safe Childbirth Checklist Activities																		Non WHO SCC Activities				
Observation Time	1. Mat Temp	2. Blood Pressure	3. Parto.	4. Checklist/ poster	5. Medication	6. Hand-wash gloves or alochol rub	7. Prep of EBS	8. Use neonatal bag mask	9. Referring a patient	10. Check Mother for bleeding	11. Examination of Newborn	12. Initiation of skin-to-skin	13. Discussing Family Planning	14. Danger signs	15. Group Discussion (if family planning and danger signs could not be observed)	16. Initiation of Breast-feeding	17. Confirmation of Vaccination	18. Non-CL Direct Patient Care	19. Admin. Duties	20. Break	21. Down-time	22. Unobserved
HOUR 1																						
:02																						
:04																						
:06																						
:08																						
:10																						
:12																						
:14																						
:16																						
:18																						
:20																						
:22																						
:24																						
:26																						
:28																						
:30																						
:32																						
:34																						
:36																						
:38																						
:40																						
:42																						
:44																						
:46																						
:48																						
:50																						
:52																						
:54																						
:56																						
:58																						

Notes:

For peer review only

Obser-vation Time	1. Mat Temp	2. Blood Pressure	3. Parto.	4. Checklist/ poster	5. Medication	6. Hand-wash gloves or alochol rub	7. Prep of EBS	8. Use neonatal bag mask	9. Referring a patient	10. Check Mother for bleeding	11. Exami-nation of Newborn	12. Initiation of skin-to-skin	13. Discuss-ing Family Planning	14. Danger signs	15. Group Discussion (if family planning and danger signs could not be observed)	16. Initiation of Breast-feeding	17. Confirm-ation of Vaccination	18. Non-CL Direct Patient Care	19. Admin. Duties	20. Break	21. Down-time	22. Unob-served
HOUR 2																						
:00																						
:02																						
:04																						
:06																						
:08																						
:10																						
:12																						
:14																						
:16																						
:18																						
:20																						
:22																						
:24																						
:26																						
:28																						
:30																						
:32																						
:34																						
:36																						
:38																						
:40																						
:42																						
:44																						
:46																						
:48																						
:50																						
:52																						
:54																						
:56																						
:58																						

Notes:

For peer review only

Obser-vation Time	1. Mat Temp	2. Blood Pressure	3. Parto.	4. Checklist/ poster	5. Medication	6. Hand-wash gloves or alochol rub	7. Prep of EBS	8. Use neonatal bag mask	9. Referring a patient	10. Check Mother for bleeding	11. Exami-nation of Newborn	12. Initiation of skin-to-skin	13. Discuss-ing Family Planning	14. Danger signs	15. Group Discussion (if family planning and danger signs could not be observed)	16. Initiation of Breast-feeding	17. Confirm-ation of Vaccination	18. Non-CL Direct Patient Care	19. Admin. Duties	20. Break	21. Down-time	22. Unob-served
HOUR 3																						
:00																						
:02																						
:04																						
:06																						
:08																						
:10																						
:12																						
:14																						
:16																						
:18																						
:20																						
:22																						
:24																						
:26																						
:28																						
:30																						
:32																						
:34																						
:36																						
:38																						
:40																						
:42																						
:44																						
:46																						
:48																						
:50																						
:52																						
:54																						
:56																						
:58																						

Notes:

For peer review only

Observation Time	1. Mat Temp	2. Blood Pressure	3. Parto.	4. Checklist/ poster	5. Medication	6. Hand-wash gloves or alochol rub	7. Prep of EBS	8. Use neonatal bag mask	9. Referring a patient	10. Check Mother for bleeding	11. Examination of Newborn	12. Initiation of skin-to-skin	13. Discussing Family Planning	14. Danger signs	15. Group Discussion (if family planning and danger signs could not be observed)	16. Initiation of Breast-feeding	17. Confirmation of Vaccination	18. Non-CL Direct Patient Care	19. Admin. Duties	20. Break	21. Down-time	22. Unobserved
HOUR 4																						
:00																						
:02																						
:04																						
:06																						
:08																						
:10																						
:12																						
:14																						
:16																						
:18																						
:20																						
:22																						
:24																						
:26																						
:28																						
:30																						
:32																						
:34																						
:36																						
:38																						
:40																						
:42																						
:44																						
:46																						
:48																						
:50																						
:52																						
:54																						
:56																						
:58																						

Notes:

For peer review only
For peer review only - http://bmjopen.bmj.com/site/about/guidelines.xhtml

Observation Time	1. Mat Temp	2. Blood Pressure	3. Parto.	4. Checklist/ poster	5. Medication	6. Handwash gloves or alochol rub	7. Prep of EBS	8. Use neonatal bag mask	9. Referring a patient	10. Check Mother for bleeding	11. Examination of Newborn	12. Initiation of skin-to-skin	13. Discussing Family Planning	14. Danger signs	15. Group Discussion (if family planning and danger signs could not be observed)	16. Initiation of Breast-feeding	17. Confirmation of Vaccination	18. Non-CL Direct Patient Care	19. Admin. Duties	20. Break	21. Downtime	22. Unobserved
HOUR 5																						
:00																						
:02																						
:04																						
:06																						
:08																						
:10																						
:12																						
:14																						
:16																						
:18																						
:20																						
:22																						
:24																						
:26																						
:28																						
:30																						
:32																						
:34																						
:36																						
:38																						
:40																						
:42																						
:44																						
:46																						
:48																						
:50																						
:52																						
:54																						
:56																						
:58																						

Notes:

For peer review only - http://bmjopen.bmj.com/site/about/guidelines.xhtml

Obser-vation Time	1. Mat Temp	2. Blood Pressure	3. Parto.	4. Checklist/ poster	5. Medication	6. Hand-wash gloves or alochol rub	7. Prep of EBS	8. Use neonatal bag mask	9. Referring a patient	10. Check Mother for bleeding	11. Exami-nation of Newborn	12. Initiation of skin-to-skin	13. Discuss-ing Family Planning	14. Danger signs	15. Group Discussion (if family planning and danger signs could not be observed)	16. Initiation of Breast-feeding	17. Confirm-ation of Vaccination	18. Non-CL Direct Patient Care	19. Admin. Duties	20. Break	21. Down-time	22. Unob-served
HOUR 6																						
:00																						
:02																						
:04																						
:06																						
:08																						
:10																						
:12																						
:14																						
:16																						
:18																						
:20																						
:22																						
:24																						
:26																						
:28																						
:30																						
:32																						
:34																						
:36																						
:38																						
:40																						
:42																						
:44																						
:46																						
:48																						
:50																						
:52																						
:54																						
:56																						
:58																						

Notes:

For peer review only

For peer review only

Observation Time	1. Mat Temp	2. Blood Pressure	3. Parto.	4. Checklist/ poster	5. Medication	6. Hand-wash gloves or alochol rub	7. Prep of EBS	8. Use neonatal bag mask	9. Referring a patient	10. Check Mother for bleeding	11. Examination of Newborn	12. Initiation of skin-to-skin	13. Discussing Family Planning	14. Danger signs	15. Group Discussion (if family planning and danger signs could not be observed)	16. Initiation of Breast-feeding	17. Confirmation of Vaccination	18. Non-CL Direct Patient Care	19. Admin. Duties	20. Break	21. Down-time	22. Unobserved
HOUR 7																						
:00																						
:02																						
:04																						
:06																						
:08																						
:10																						
:12																						
:14																						
:16																						
:18																						
:20																						
:22																						
:24																						
:26																						
:28																						
:30																						
:32																						
:34																						
:36																						
:38																						
:40																						
:42																						
:44																						
:46																						
:48																						
:50																						
:52																						
:54																						
:56																						
:58																						

Notes:

Observation Time	1. Mat Temp	2. Blood Pressure	3. Parto.	4. Checklist/ poster	5. Medication	6. Hand-wash gloves or alochol rub	7. Prep of EBS	8. Use neonatal bag mask	9. Referring a patient	10. Check Mother for bleeding	11. Examination of Newborn	12. Initiation of skin-to-skin	13. Discussing Family Planning	14. Danger signs	15. Group Discussion (if family planning and danger signs could not be observed)	16. Initiation of Breast-feeding	17. Confirmation of Vaccination	18. Non-CL Direct Patient Care	19. Admin. Duties	20. Break	21. Down-time	22. Unobserved
HOUR 8																						
:00																						
:02																						
:04																						
:06																						
:08																						
:10																						
:12																						
:14																						
:16																						
:18																						
:20																						
:22																						
:24																						
:26																						
:28																						
:30																						
:32																						
:34																						
:36																						
:38																						
:40																						
:42																						
:44																						
:46																						
:48																						
:50																						
:52																						
:54																						
:56																						
:58																						

Notes:

For peer review only

Appendix Table A5: General task categories in work sampling

General Group	Specific Activity
Checklist (CL)	Temperature<br>Blood pressure<br>Partograph<br>Paper checklist interaction<br>Medication<br>Handwashing<br>Prep of essential supplies<br>Neonatal bag mask<br>Referral<br>Check mother for bleeding<br>Examine newborn<br>Skin-to-skin initiation<br>Discuss family planning<br>Explain danger signs<br>Breastfeeding initiation<br>Confirm vaccination
Non-Checklist Clinical	Non-CL Direct Patient Care
Administrative	Admin. Duties
Downtime	Break<br>Downtime

For peer review only

Appendix Table A6: Task-time estimates for Essential Birth Practices

Essential Birth Practices	Time Source	Mean	SE	Min (seconds)	Max	Sample Size
Referral	Direct measurement	127	16	16	330	21
	Self-report	256	34	90	720	22
Temperature	Direct measurement	94	4	3	275	92
	Self-report	60	0	60	60	2
Check mother for bleeding	Direct measurement	92	12	2	320	37
	Self-report	178	22	60	300	14
Neonatal bag mask use	Direct measurement	76	16	6	300	22
	Self-report	175	23	60	300	11
Blood pressure	Direct measurement	74	2	20	165	126
	Self-report			N/A		N/A
Preparation of essential supplies	Direct measurement	59	18	8	436	31
	Self-report	202	29	120	600	18
Paper checklist interaction	Direct measurement	54	3	4	180	175
	Self-report	210	46	150	300	3
Explain danger signs	Direct measurement	40	4	8	109	41
	Self-report	258	14	60	600	65
Partograph	Direct measurement	38	3	9	88	38
	Self-report	164	16	30	240	15
Discuss family planning	Direct measurement	36	5	4	94	30
	Self-report	316	16	60	600	60
Examine newborn	Direct measurement	34	3	4	177	143
	Self-report	184	30	60	600	17
Assess baby's breathing	Direct measurement	31	6	10	60	9
	Self-report			N/A		N/A
Medication	Direct measurement	29	1	1	150	419
	Self-report	225	75	150	300	2
Handwashing	Direct measurement	29	1	1	81	208
	Self-report	180	11	150	210	6
Breastfeeding initiation	Direct measurement	24	2	6	60	43
	Self-report	223	35	120	420	9
Skin-to-skin initiation	Direct measurement	20	2	3	93	89
	Self-report			N/A		N/A
Weight	Direct measurement	18	2	5	44	35
	Self-report			N/A		N/A

Appendix Table A7: Heat map of self-reported most time-consuming Checklist tasks*

Checklist Activity	Rank 1	Rank 2 (Number of Respondents)	Rank 3	Total
Discussing family planning	41	16	3	60
Explaining danger signs	22	31	12	65
Prep of EBS	5	3	10	18
Check mother for bleeding	3	4	7	14
Initiation of breastfeeding	3	2	4	9
Referring a patient	3	11	8	22
Partograph	2	3	10	15
Use neonatal bag mask	2	1	8	11
Examination of newborn	1	4	12	17
Handwash gloves or alcohol rub	1	3	2	6
Checklist/ poster	0	2	1	3
Confirmation of vaccination	0	2	3	5
Medication	0	1	1	2
Temperature	0	0	2	2

*Number of staff reporting activity in each rank position; total staff interviewed = 83

For peer review only - http://bmjopen.bmj.com/site/about/guidelines.xhtml

Appendix Figure A1: Median labor and delivery ward patient load by hour (time of day) (1319 facility-hour observations; one facility-hour dropped in hour 20)

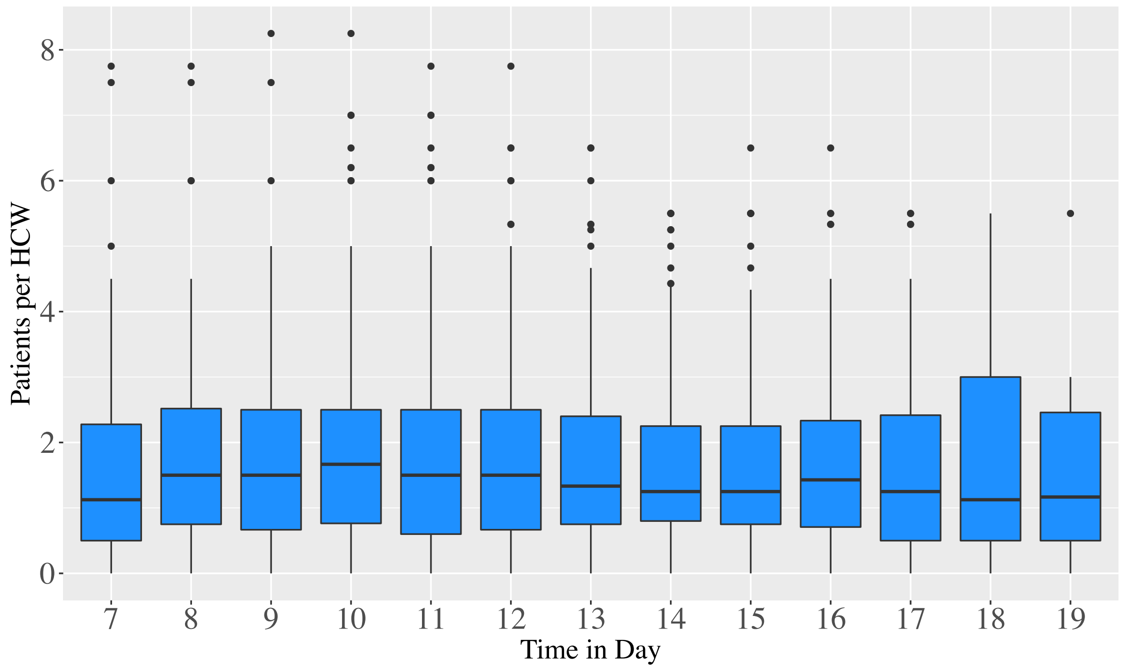

Appendix Figure A2: Median percent of hour on break by patient load per HCW (1320 facility-hour observations)

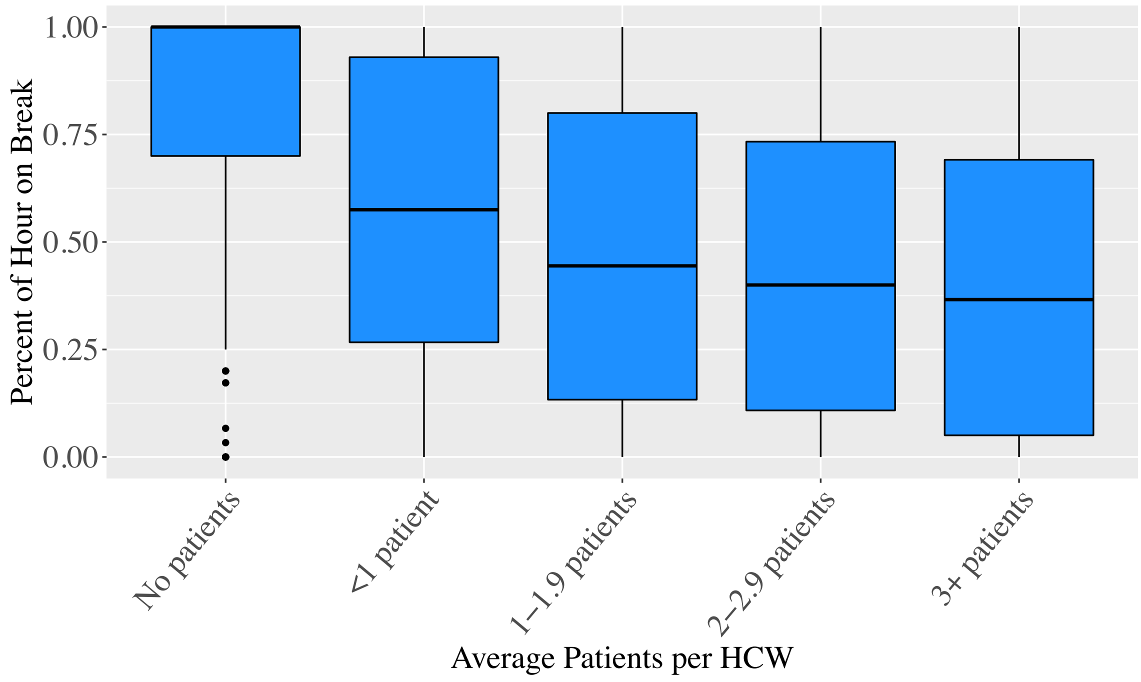

CONSORT 2010 checklist of information to include when reporting a randomised trial*

Section/Topic	Item No	Checklist item	Reported on page No
**Title and abstract**			
	1a	Identification as a randomised trial in the title	1
	1b	Structured summary of trial design, methods, results, and conclusions (for specific guidance see CONSORT for abstracts)	6
**Introduction**			
Background and objectives	2a	Scientific background and explanation of rationale	4-5
	2b	Specific objectives or hypotheses	6
**Methods**			
Trial design	3a	Description of trial design (such as parallel, factorial) including allocation ratio	6
	3b	Important changes to methods after trial commencement (such as eligibility criteria), with reasons	N/A
Participants	4a	Eligibility criteria for participants	6
	4b	Settings and locations where the data were collected	6
Interventions	5	The interventions for each group with sufficient details to allow replication, including how and when they were actually administered	6-8
Outcomes	6a	Completely defined pre-specified primary and secondary outcome measures, including how and when they were assessed	10-12
	6b	Any changes to trial outcomes after the trial commenced, with reasons	N/A
Sample size	7a	How sample size was determined	See protocol paper
	7b	When applicable, explanation of any interim analyses and stopping guidelines	N/A
Randomisation:			
Sequence generation	8a	Method used to generate the random allocation sequence	See protocol paper
	8b	Type of randomisation; details of any restriction (such as blocking and block size)	6 + abstract
Allocation concealment mechanism	9	Mechanism used to implement the random allocation sequence (such as sequentially numbered containers), describing any steps taken to conceal the sequence until interventions were assigned	See protocol paper
Implementation	10	Who generated the random allocation sequence, who enrolled participants, and who assigned participants to	See protocol

For peer review only - http://bmjopen.bmj.com/site/about/guidelines.xhtml

		interventions	paper
Blinding	11a	If done, who was blinded after assignment to interventions (for example, participants, care providers, those assessing outcomes) and how	N/A
	11b	If relevant, description of the similarity of interventions	N/A
Statistical methods	12a	Statistical methods used to compare groups for primary and secondary outcomes	Descriptive analysis in RCT
	12b	Methods for additional analyses, such as subgroup analyses and adjusted analyses	N/A
**Results**			
Participant flow (a diagram is strongly recommended)	13a	For each group, the numbers of participants who were randomly assigned, received intended treatment, and were analysed for the primary outcome	6 and 10-12
	13b	For each group, losses and exclusions after randomisation, together with reasons	N/A
Recruitment	14a	Dates defining the periods of recruitment and follow-up	7
	14b	Why the trial ended or was stopped	7
Baseline data	15	A table showing baseline demographic and clinical characteristics for each group	See main RCT results paper
Numbers analysed	16	For each group, number of participants (denominator) included in each analysis and whether the analysis was by original assigned groups	See main RCT results paper
Outcomes and estimation	17a	For each primary and secondary outcome, results for each group, and the estimated effect size and its precision (such as 95% confidence interval)	See main RCT results paper
	17b	For binary outcomes, presentation of both absolute and relative effect sizes is recommended	N/A
Ancillary analyses	18	Results of any other analyses performed, including subgroup analyses and adjusted analyses, distinguishing pre-specified from exploratory	All pre-specified measures
Harms	19	All important harms or unintended effects in each group (for specific guidance see CONSORT for harms)	N/A
**Discussion**			
Limitations	20	Trial limitations, addressing sources of potential bias, imprecision, and, if relevant, multiplicity of analyses	12-15
Generalisability	21	Generalisability (external validity, applicability) of the trial findings	12-15
Interpretation	22	Interpretation consistent with results, balancing benefits and harms, and considering other relevant evidence	12-15

For peer review only

**Other information**			
Registration	23	Registration number and name of trial registry	In submission fields
Protocol	24	Where the full trial protocol can be accessed, if available	Published manuscript included in submission
Funding	25	Sources of funding and other support (such as supply of drugs), role of funders	In submission fields

*We strongly recommend reading this statement in conjunction with the CONSORT 2010 Explanation and Elaboration for important clarifications on all the items. If relevant, we also recommend reading CONSORT extensions for cluster randomised trials, non-inferiority and equivalence trials, non-pharmacological treatments, herbal interventions, and pragmatic trials. Additional extensions are forthcoming: for those and for up to date references relevant to this checklist, see www.consort-statement.org.

For peer review only

BMJ Open

Estimating maternity ward birth attendant time use in India: A microcosting study

Journal:	*BMJ Open*
Manuscript ID	bmjopen-2021-054164.R1
Article Type:	Original research
Date Submitted by the Author:	17-Sep-2021
Complete List of Authors:	Lofgren, Katherine; Harvard University, Interfaculty Initiative in Health Policy<br>Bobanski , Lauren ; Ariadne Labs, Harvard T.H. Chan School of Public Health/Brigham and Women's Hospital, 401 Park Drive, 3rd floor West<br>Tuller, Danielle; Harvard TH Chan School of Public Health / Brigham and Women's Hospital<br>Singh, Vinay P.; Population Services International; Community Empowerment Lab<br>Marx Delaney , Megan; Ariadne Labs; Harvard T. H. Chan School of Public Health<br>Jurczak, Amanda; Ariadne Labs<br>Ragavan, Meera; University of California San Francisco, Department of Medicine, Division of Hematology/Oncology<br>Kalita, Tapan; Piramal Swasthya Management and Research Institute<br>Karlage, Ami; Ariadne Labs<br>Resch, Stephen; Harvard University T H Chan School of Public Health, Center for Health Decision Science<br>Semrau, Katherine; Brigham and Women's Hospital, Ariadne Labs; Brigham and Women's Hospital, Division of Global Health Equity & Department of Medicine
**Primary Subject Heading**:	Global health
Secondary Subject Heading:	Obstetrics and gynaecology
Keywords:	Health economics < HEALTH SERVICES ADMINISTRATION & MANAGEMENT, Maternal medicine < OBSTETRICS, Quality in health care < HEALTH SERVICES ADMINISTRATION & MANAGEMENT

SCHOLARONE™
Manuscripts

I, the Submitting Author has the right to grant and does grant on behalf of all authors of the Work (as defined in the below author licence), an exclusive licence and/or a non-exclusive licence for contributions from authors who are: i) UK Crown employees; ii) where BMJ has agreed a CC-BY licence shall apply, and/or iii) in accordance with the terms applicable for US Federal Government officers or employees acting as part of their official duties; on a worldwide, perpetual, irrevocable, royalty-free basis to BMJ Publishing Group Ltd ("BMJ") its licensees and where the relevant Journal is co-owned by BMJ to the co-owners of the Journal, to publish the Work in this journal and any other BMJ products and to exploit all rights, as set out in our licence.

The Submitting Author accepts and understands that any supply made under these terms is made by BMJ to the Submitting Author unless you are acting as an employee on behalf of your employer or a postgraduate student of an affiliated institution which is paying any applicable article publishing charge ("APC") for Open Access articles. Where the Submitting Author wishes to make the Work available on an Open Access basis (and intends to pay the relevant APC), the terms of reuse of such Open Access shall be governed by a Creative Commons licence – details of these licences and which Creative Commons licence will apply to this Work are set out in our licence referred to above.

Other than as permitted in any relevant BMJ Author's Self Archiving Policies, I confirm this Work has not been accepted for publication elsewhere, is not being considered for publication elsewhere and does not duplicate material already published. I confirm all authors consent to publication of this Work and authorise the granting of this licence.

For peer review only

Title: **Estimating maternity ward birth attendant time use in India: A microcosting study**

Corresponding Author: **Katherine T. Lofgren**
Harvard University, Interfaculty Initiative in Health Policy
14 Story St.
Cambridge, MA. 02138
kate.lofgren@gmail.com

Co-Authors:

Lauren Bobanski
Ariadne Labs, Brigham & Women's Hospital and Harvard T.H. Chan School of Public Health, Boston, MA., USA
Danielle E. Tuller
Ariadne Labs, Brigham & Women's Hospital and Harvard T.H. Chan School of Public Health, Boston, MA., USA
Vinay P. Singh
Population Services International, Lucknow, India
Community Empowerment Lab, Lucknow, India
Megan Marx Delaney
Ariadne Labs, Brigham & Women's Hospital and Harvard T.H. Chan School of Public Health, Boston, MA., USA
Amanda Jurczak
Ariadne Labs, Brigham & Women's Hospital and Harvard T.H. Chan School of Public Health, Boston, MA., USA
Meera Ragavan
University of California San Francisco, Department of Medicine, Division of Hematology/Oncology, San Francisco, CA.
Tapan Kalita
Piramal Swasthya Management and Research Institute, Hyderabad, India
Ami Karlage
Ariadne Labs, Brigham & Women's Hospital and Harvard T.H. Chan School of Public Health, Boston, MA., USA
Stephen Resch
Harvard Center for Health Decision Sciences, Harvard T.H. Chan School of Public Health, Boston, MA. USA
Katherine E. A. Semrau
Ariadne Labs, Brigham & Women's Hospital and Harvard T.H. Chan School of Public Health, Boston, MA., USA
Department of Medicine, Harvard Medical School, Boston, MA. USA
Division of Global Health Equity, Brigham & Women's Hospital, Boston, MA. USA

Word Count: 3157

For peer review only - http://bmjopen.bmj.com/site/about/guidelines.xhtml

Abstract:

Objectives

Despite global concern over the quality of maternal care, little is known about the time requirements to complete essential birth practices. Using three micro-costing data collection methods within the BetterBirth trial, we aimed to assess the birth attendant time use and the specific time requirements to incorporate the WHO Safe Childbirth Checklist into clinical practice.

Setting

We collected detailed survey data on birth attendant time-use within the BetterBirth trial in Uttar Pradesh, India. The BetterBirth trial tested whether the peer-coaching-based implementation of the WHO Checklist was effective in improving the quality of facility-based childbirth care.

Participants

We collected measurements of time-to-completion for 18 essential birth practices from July 2016 through October 2016 across 10 facilities in 5 districts (1559 total timed observations). An anonymous survey asked about the impact of the WHO Checklist on birth attendants at every intervention facility (15 facilities, 83 respondents) in the Lucknow hub. Additionally, data collectors visited facilities to conduct a census of patients and birth attendants across 20 facilities in 7 districts between June 2016 to November 2016 (610 2-hour facility observations).

Primary and secondary outcome measures

The primary outcome measure of this study is the percent of staff time required to complete the essential birth practices included in the WHO Checklist.

Results

When birth attendants were timed, we found practices (such as handwashing, use of neonatal bag mask, and skin-to-skin initiation) were completed rapidly (18 seconds – 2 minutes). As the patient load increased, time dedicated to clinical care increased but remained low relative to administrative and downtime.

Conclusions

On average, WHO Checklist clinical care accounted for less than 7% of birth attendant time-use per hour. However, questions remain regarding the performance quality of practices and how to accurately capture and interpret idle and break time.

Trial registration

NCT02148952

For peer review only - http://bmjopen.bmj.com/site/about/guidelines.xhtml

Strengths and Limitations

- Few studies include micro-costing data collection to estimate the time-requirements of a proposed policy or clinical intervention.
- The combination of three distinct time-use capture methods creates a rich understanding of how birth attendants use their time and how long specific tasks take to complete
- Both stop-watch and birth attendant reported time-to-complete individual evidence-based practices were shorter than expected *a priori*. Self-reported time-to-complete individual evidence-based practices were longer on average than stop-watched derived estimates.
- Even during times with high patient volume, administrative and downtime were the prevailing time-use categories of birth attendants. However, we are not able to distinguish between true downtime and watchful waiting in an active clinical care setting based on our methods.

For peer review only

INTRODUCTION

Remarkable achievements have been made in the reduction of maternal and neonatal mortality globally.[1,2] One of the primary achievements of the Millennium Development Goals Era was increased rate of facility-based childbirth.[3] However, evidence suggests increased coverage of services does not necessarily lead to mortality and morbidity reductions.[4,5] A large portion of stillbirths and maternal and neonatal deaths remain preventable with timely, high-quality care.[6–8] As low- and middle-income countries (LMICs) continue to expand access to services, ensuring patients receive high-quality, evidence-based clinical care is essential for continued progress in population health.[9]

The use of evidence-based care in labor and delivery facilities remains low.[10,11] Even when women reach a facility in a timely manner, without adequate and appropriate treatment, preventable deaths occur.[12] Many interventions seek to improve the quality of care in LMIC health systems by increasing the number of essential birth practices performed for each laboring mother.[9] The World Health Organization's Safe Childbirth Checklist (Checklist) is one such effort.[13] The Checklist is a clinical care aid that synthesizes and prioritizes evidence-based essential birth practices (practices) from admission to discharge in order to increase the number of practices—like handwashing, checking the mother for bleeding, or discussing family planning—performed by birth attendants (BAs) at the point of care. Defining essential practices and creating mechanisms like the Checklist for clinical staff to consistently implement those practices has been successful across a diverse set of clinical contexts in both high- and low-income settings.[14–16]

For peer review only

One complicating factor in quality improvement efforts targeting labor and delivery wards specifically is staff time availability. Across health systems, BAs often report feeling overwhelmed and busy.[17] Additionally, staffing shortages are a known barrier to timely, high-quality clinical care.[18] With any quality-improvement intervention, clinical care may increase staffing time demands or replace existing low-value activities. The implementation of quality-improvement interventions requires understanding existing staff time capacity at baseline and how staff time-use changes post-implementation.

The BetterBirth trial was a matched-pair, cluster-randomized, controlled trial of a coaching-based implementation of the Checklist in Uttar-Pradesh, India to test the effect of the intervention on a composite outcome of perinatal mortality, maternal mortality, or maternal severe complication within 7 days of giving birth.[19,20] Embedded in the BetterBirth trial, we conducted data collection to measure the time-demands of the Checklist practices, with the primary intent of informing a cost-effectiveness analysis (CEA) of the BetterBirth trial. When the main outcome of the BetterBirth trial was a null effect on maternal and neonatal mortality and morbidity, the CEA was rendered irrelevant. However, concerns remained about the possibility that the Checklist introduced a significant time burden on BAs. Prior to the implementation of the BetterBirth trial, BAs often reported feeling overwhelmed and busy. As a result, we used the collected data to answer the following questions:

(1) What is the time-burden of the practices included in the Checklist?

(2) Do BAs perceive the Checklist as a significant stress- or time-burden?

(3) How does BA time-use change as their patient load increases?

For peer review only - http://bmjopen.bmj.com/site/about/guidelines.xhtml

METHODS

Study setting

The BetterBirth trial was a peer-coaching-based implementation of the Checklist in Uttar Pradesh, India. The matched-pair, cluster-randomized, controlled trial randomized the BetterBirth trial across 120 facilities (60 control, 60 treatment) with a study population of women and their newborns, the birth attendants (BAs) providing care.. Study facilities had more than 1,000 deliveries per year and minimum of four labor and delivery staff. The study protocol and results have been published and include further details on the study population, design, and methods used to test the primary outcome of interest, maternal and perinatal mortality and morbidity outcomes.[19,20]

This paper details three time-use data collection methods to triangulate the time-burden of the Checklist practices within the broader time-demands on BAs within the BetterBirth trial. Data collectors (N=16) were junior nurses who received training and supportive supervision for data quality assurance across all three data collection methods (each described in more detail in subsections below). We captured 18 specific Checklist practices (Appendix Table A1) as well as non-Checklist clinical care, administrative duties, and break/downtime. Although the intention was to distinguish between a scheduled break and non-scheduled downtime, efforts to delineate between these two activities by data collectors was difficult in practice. For the purposes of this paper, ‘downtime’ refers to a mix of scheduled breaks as well as idle time for other reasons, such as no patients or watchful waiting during clinical care. We first measured time-to-completion for 18 practices via direct BA observation during clinical practice (time-demand). We then surveyed BAs about their experience during the BetterBirth trial (perceived time-demand). Finally, we

For peer review only - http://bmjopen.bmj.com/site/about/guidelines.xhtml

visited facilities and conducted both a census of births as well as observing clinical care activities at regular intervals (BA time-use).

The time-demand of Checklist practices

We collected measurements of Checklist practice time-to-completion for 18 practices over a four-month period from July 2016 through October 2016 across 10 facilities in 5 districts. Data collectors visited each facility 2-3 times per week, for 8 hours shifts between 7am-3pm or 11am-7pm. If available, a second data collector or a supervisor performed data quality assurance activities. Time-to-complete tasks were assessed by the data collectors with stopwatches, recorded on paper (Appendix Table A2), and transferred to an Excel spreadsheet. The time measurements were used to estimate the time required to complete each Checklist practice.

Perceived time-demand of the Checklist by BAs

We also surveyed BAs on their time-burden perceptions. The anonymous survey asked general questions about the impact of the Checklist on the daily routines and workloads of BAs (83 respondents) at every intervention facility (15 facilities) in the Lucknow hub (the cost-effectiveness data collection survey region with 30 total facilities) from June to July 2016. All staff working at the facility on the day of data collection were provided the survey and could answer anonymously. The survey also asked respondents to rank the top three most time-consuming items on the Checklist and estimate the time required to complete those tasks. The specific time estimates for Checklist practices were used to supplement and compare with the stopwatch time measurements.

BA time-use in the labor and delivery ward

For peer review only - http://bmjopen.bmj.com/site/about/guidelines.xhtml

How providers use their time depends on both the patient demand and the number of BAs on duty. To estimate the patient demand and health care labor supply, data collectors visited facilities to conduct a census of patients and BAs every 2 hours, recording the results on a paper form (Appendix Table A4-5). Observations were taken across 20 facilities in 7 districts from June 2016 to November 2016. This data was used to calculate the average number of patients per BA at given facilities and times of day.

In addition to the census, we also observed BAs conducting regular care (a work sampling approach) to capture the proportion of time spent on various types of clinical and non-clinical work.[21] A data collector visited a facility and, for each hour observed, recorded the type of activity the BA was engaged in at pre-specified 2-minute intervals on a paper form (Appendix Table A3-4). For example, if at 11:00 a.m. the BA was using a neonatal bag and mask, the data collector recorded that activity. At 11:02 a.m. the data collector would again record what the BA was doing; in some cases, she might still be using a neonatal bag and mask, while in other cases, there may be a new activity listed such as non-Checklist direct patient care. This type of data provides estimates of proportional time spent on various activities but does not directly estimate the time required for specific tasks.

If there were at least two BAs on duty at the same time, observations alternated between two BAs. For example, the 11:00 AM observation would pertain to BA1 while the 11:02 AM observation would pertain to BA2, alternating back and forth throughout the hour. If only one BA was available for observation, an observation was taken every 2 minutes for their work. We calculate the proportion of each BA's time spent in different general activity categories to

For peer review only - http://bmjopen.bmj.com/site/about/guidelines.xhtml

estimate the overall time-use in given facility-hours (Appendix Table A5 maps specific activities to general categories).

Ethics approval

The study protocol was approved by the ethics committees of: Community Empowerment Lab (Ref no: 2014006), Jawaharlal Nehru Medical College (Ref no: MDC/IECHSR/2015–16/A-53), Harvard T.H. Chan School of Public Health (Protocol 21 975–102), Population Services International (Protocol ID: 47·2012), WHO (Protocol ID: RPC 501) and Indian Council of Medical Research. The protocol was reviewed and re-approved on an annual basis. We obtained consent from each facility's leadership for trial participation and data collection on eligible mothers from facility registers. Birth attendants and facility staff verbally agreed to participate prior to trial initiation. Independent observers obtained written consent from women or their surrogates and verbal consent from birth attendants prior to observation.

Public involvement in research

Patient and provider representatives worked with us to refine the Checklist when it was originally designed in 2009. The BetterBirth trial study research question and design did not have direct patient involvement, but did have a scientific advisory committee that included clinicians, researchers, government officials who work in the same area. We did receive and modified the dissemination plan based on feedback from providers and government partners for each participating facility/district. Further, we published a report for wider dissemination and audience found at: betterbirth.ariadnelabs.org

For peer review only - http://bmjopen.bmj.com/site/about/guidelines.xhtml

RESULTS

The time-demand of Checklist practices

Across all Checklist practices, a total of 1,559 practices were directly timed from 35 unique birth attendants (BAs) across 10 facilities during clinical care (see Appendix Table A6 for practice-specific sample sizes). Handwashing (N=419) and the administration of medication (N=208) were the most frequently observed direct measurements, while referrals (N=21) and the assessment of the baby's breathing (N=9) had the fewest recorded observations. Directly measured task-times revealed a pattern of rapid time-to-complete practices on the Checklist. When Checklist practices were directly measured using stopwatches, the average time-to-complete the task ranged from 127 seconds (a referral) to 18 seconds (weighing the baby). Tasks like breastfeeding initiation and discussing family planning that require conversations and (potentially) complex patient-BA interactions both took less than 1 minute on average (black dots on Figure 1 and Appendix Table A6). Over 70% (N=12 out of 18 practices) of the average time-to-complete measurements were less than one minute.

Perceived time-demand of the Checklist by BAs

Across 15 facilities, there were 83 total respondents to the survey. The majority of BAs responded that the Checklist made their jobs easier (96%; N=80). When BAs were asked if the Checklist took away from non-Checklist activities, only 17% of responders felt other clinical duties were rushed (N=11) or their workday was prolonged (N=3).

For peer review only - http://bmjopen.bmj.com/site/about/guidelines.xhtml

Respondents were asked to rank the three most time-consuming Checklist practices and estimate the time required to complete those three tasks. Discussing family planning was the most frequently reported time-consuming activity (ranked #1 by 49% of BAs (Appendix Table A7)). All tasks were estimated by BAs to take less than 5:07 minutes on average. The self-reported task-times were longer than the direct measurements, particularly in discussion-based practices like explaining danger signs and discussion family planning (Figure 1).

BA time-use in the labor and delivery ward

BA time-use incorporates data from the work sampling time-use data collection and the facility BA and patient census. In total, 610 2-hour facility periods were recorded for the patient census and 27,768 individual task observations were recorded in our work sampling survey. Within the hours of data collection (7am-7pm), we found relatively constant median patient-load at 1.4 patients-per-BA with large variability in the potential patient-load for any given facility-hour (0 to 8 patients-per-BA observed range) (Appendix Figure A1).

Clinical care (both non-Checklist and Checklist) was 21% of the average facility-staff hour. As patients-per-BA increased, so did clinical care and administrative duties. When there were no patients, BAs spent the majority of their time in downtime (80% of time) or conducting administrative tasks (15% of time). Once the patient-load increased to 1-2 patients-per-BA, BA time-use shifted towards clinical care (23% of BA time) as well as administrative tasks (26% up from 15% with no patients). At 3 or more patients-per-BA, the Checklist accounted for 7% of BA time (out of a total 24% of the hour spent on clinical care). However, even at high patient-loads (3+), the most common time-use, on average, was still recorded as downtime (40% in

For peer review only - http://bmjopen.bmj.com/site/about/guidelines.xhtml

downtime compared to 24% in clinical care; Figure 2). Sample size breakdowns for individual work sampling observations by patient load and task-type are available in Appendix Table A8.

However, the average BA downtime is a misleading statistic. When the full distribution of BA downtime by facility-BA-hour are graphed, there is clear heterogeneity in the distribution that is not captured by summary measures like the mean or median percent of the hour spent in downtime. Particularly for the 1-2 patient categorization, there is a clear bi-modal trend with BAs spending the majority of staff-hours either completely in downtime or without any downtime. Similarly, for the 0 and 3+ patient categories, the distributions are highly skewed to all downtime (0 patients) and no downtime (3+ patients). Taken across all these categories, summary statistics mask the extreme downtime dichotomy experienced in practice by BAs (Figure 3, Appendix Figure A2). Sample size breakdowns for individual work sampling observations by patient load and task-type are available in Appendix Table A9.

DISCUSSION

The time-demand on BAs is an important piece of the maternal and newborn quality-of-care puzzle. Quality improvement efforts inherently require staff time to shift away from existing time uses and towards evidence-based practices such as those included in the Checklist. Using three different data collection efforts, we found that the Checklist practices were not an undue time burden on BAs. However, based on our data, we are not confident that practices were performed at sufficient quality. Further, our results show a high proportion of ambiguously measured downtime, a lesson to learn from in future studies to differentiate watchful waiting from true downtime. Concerns about the quality of care provided are consistent with the overall

For peer review only - http://bmjopen.bmj.com/site/about/guidelines.xhtml

BetterBirth trial findings—treatment facilities did not have reduced mortality and morbidity after the Checklist was implemented.

Several of the time-to-complete practice measures seemed implausibly fast to be of sufficient quality. In particular, tasks like initiation of breastfeeding, initiation of skin-to-skin contact, discussion or family planning, and referrals likely require more time in expectation than is currently being allocated based on our study results. Referrals, which were the most time-consuming task overall, were still completed within 2 minutes. Given the difficulty of breastfeeding initiation,[22,23] it is unlikely that a mean task-time of 24 seconds (SE = 16 seconds) accurately captures the true time required to successfully initiate breastfeeding. In other cases, the timing seems plausible. For example, the CDC recommends handwashing for 15-20 seconds.[24] In our sample, handwashing took an average of 29 seconds. Similarly, although the self-reported sample of task-time estimates is skewed towards tasks that the BAs perceived as relatively more burdensome, the self-reported time-to-complete tasks remained lower than expected *a priori*. These results are similarly indicative that time-to-complete Checklist-related practices are too low to have been consistently performed at high-quality. Further research is needed to estimate minimum time requirements for the performance of practices at high quality and how the Checklist, when implemented at high-quality, impacts the staffing needs of a facility. One potential downside of a checklist-based intervention is a desire to get through the items as quickly as possible rather than at the pace required to perform each task at high-quality. Further quality approaches may also consider how to incorporate incentives for not just completing a checklist but reaching quantifiable quality benchmarks for the checklist items.

For peer review only - http://bmjopen.bmj.com/site/about/guidelines.xhtml

Although individual practices took less than 2 minutes to complete on average and overall less than 5 minutes for the full practice list, it is still possible that the workload of clinical care (and/or administrative tasks) before the introduction of the Checklist was sufficiently demanding that BAs did not have the slack to take on any incremental tasks newly introduced with the Checklist. Across all our data collection methods, however, high-quality clinical care was not the major time-use of BAs in the BetterBirth study population. One of the main open questions from our time-use data collection is how to understand and estimate the time-constraints faced by labor and delivery ward BAs. The nature of labor and delivery ward care requires long periods of waiting followed by high-stress, high-demand moments of clinical care. Could moments of inactivity actually be high-stress, high-alert contexts compared to times when the BA is truly on break? How should we differentiate between breaks that are necessary versus time that could be reallocated towards high-quality clinical care? How would the percent of time spent conducting clinical care change if quality of care improved? Our data highlights the importance and difficulty of estimating supply-side constraints in the highly unpredictable context of labor and delivery wards. In the future, it will be important to continue to estimate how quality improvement interventions impact the time-use of providers including work to parse out time which appears to be free, but in reality may be a version of alert waiting.

For peer review only

Ensuring quality care at facilities not only requires thoughtful clinical care practices, it also requires staffing strategies.[25–29] Our data collection efforts add empiric evidence on how BAs in Uttar Pradesh, India use their time across both clinical and non-clinical care under varying levels of patient demand. In future implementations of the Checklist, our data on the time-to-complete clinical tasks as well as the time-use of BAs can serve as both a model of how to

For peer review only - http://bmjopen.bmj.com/site/about/guidelines.xhtml

collect data and as a baseline for potential data collection improvements that could address lingering questions raised in this paper.

There are several limitations in our methods and data collection. Although we began with separate categories for breaks and downtime, this distinction was not clear during the actual observation. We cannot reliably distinguish true breaks from watchful waiting. Practices were meant to be timed from start to finish, pausing for breaks. For instance, if a family planning discussion began but was interrupted by breastfeeding initiation, the stopwatch should have been stopped and restarted when the family planning discussion restarted to capture the overall time required for that practice. Given the consistently short task-time estimates, this may not have occurred. In our survey of BAs, our sample size is relatively small (N=83), the responses may not generalize to the broader BA population in our study and Uttar Pradesh more broadly. Instead of asking the BAs to estimate the task-time for all 18 practices, we only asked for the top three in an effort to keep the survey short. However, it limits our self-reported task-times to only those activities that BAs considered especially time consuming, biasing the self-reported results upwards.

There are often calls for measurable indicators of health care quality. In the recent Lancet Global Health Commission on High Quality Health Systems, many of the available quality-metrics rely on the proportion or number of evidence-based practices performed.[9] Although completion of tasks is important, our evidence suggests simply performing evidence-based care does not itself ensure quality. This outcome mirrors the message that coverage of services does not equate to quality. When future quality-improvement and evidence-based care interventions are

For peer review only - http://bmjopen.bmj.com/site/about/guidelines.xhtml

implemented, it will remain important to understand how the intervention fits within the broader responsibilities and time demands of BAs as well as estimating time demands by facility-type. Quality care requires essential care is completed at a satisfactory level beyond simple completion of tasks.

ACKNOWLEDGMENTS
We recognize the Governments of India and Uttar Pradesh for collaboration and support to conduct this trial in public health facilities. We thank the facility staff, women and newborns for their participation in the study. We are indebted to Sriya Srikrishnan, Ezinne Eze-Ajoku, Bharath Kumar, Krishna Kumar, the late Dr. Narender Sharma, and all the data collectors on the facility-based data associate team. We are grateful to the members of the trial's Scientific Advisory Committee who contributed crucial guidance to the development of this study protocol.

CONTRIBUTORSHIP STATEMENT
KEAS, SR, and KTL conceived of the study. KTL conducted the analysis. VPS and TK supervised field operations and reviewed survey instruments. KTL, AK, KEAS, and LB wrote the manuscript. AK provided extensive edits and manuscript support. LB, DET, MMD, and AJ provided operational support for the study. MR provided clinical inputs for the study. All authors (KEAS, LB, DET, VPS, MMD, AJ, MR, TK, AK, SR, and KTL) reviewed the manuscript and provided edits.

COMPETING INTERESTS
The authors have no competing interests to declare.

FUNDING
Bill and Melinda Gates Foundation (OPP1017378)

DATA SHARING
Data are available in a public, open access repository. Data are available on the Harvard Dataverse Platform under the BetterBirth Dataverse website. This can be found at: https://dataverse.harvard.edu/dataverse/BetterBirthData

For peer review only - http://bmjopen.bmj.com/site/about/guidelines.xhtml

REFERENCES

1. Hogan DR, Stevens GA, Hosseinpoor AR, et al. Monitoring universal health coverage within the Sustainable Development Goals: development and baseline data for an index of essential health services. *Lancet Glob Health* 2018 Feb;6(2):e152-e168. doi: 10.1016/S2214-109X(17)30472-2.

2. Lozano R, Wang H, Foreman KJ, et al. Progress towards Millennium Development Goals 4 and 5 on maternal and child mortality: an updated systematic analysis. *The Lancet*. 2011 Sep 24;378(9797):1139-65. doi: 10.1016/S0140-6736(11)61337-8.

3. United Nations Millennium Development Goals [Internet]. [access date 2018 Sep]. Available from: http://www.un.org/millenniumgoals/maternal.shtml

4. Ng M, Misra A, Diwan V, et al. An assessment of the impact of the JSY cash transfer program on maternal mortality reduction in Madhya Pradesh, India. *Glob Health Action*. 2014 Dec 3;7:24939. doi: 10.3402/gha.v7.24939.

5. Okeke, Edward N. and Amalavoyal V. Chari, Can Institutional Deliveries Reduce Newborn Mortality? Evidence from Rwanda. Santa Monica, CA: *RAND Corporation*, 2015. https://www.rand.org/pubs/working_papers/WR1072.html

6. Ronsmans C, Scott S, Qomariyah SN, et al. Professional assistance during birth and maternal mortality in two Indonesian districts. *Bull World Health Organ*. 2009 Jun;87(6):416-23. doi: 10.2471/blt.08.051581.

7. Lawn JE, Blencowe H, Pattinson R, et al. Stillbirths: Where? When? Why? How to make the data count? *The Lancet*. 2011 Apr 23;377(9775):1448-63. doi: 10.1016/S0140-6736(10)62187-3.

8. Kruk ME, Gage AD, Joseph NT, et al. Mortality due to low-quality health systems in the universal health coverage era: a systematic analysis of amenable deaths in 137 countries. *The Lancet*. 2018 Nov 17;392(10160):2203-2212. doi: 10.1016/S0140-6736(18)31668-4.

9. Kruk ME, Gage AD, Arsenault C, et al. High-quality health systems in the Sustainable Development Goals era: time for a revolution. *Lancet Glob Health*. 2018 Nov;6(11):e1196-e1252. doi: 10.1016/S2214-109X(18)30386-3.

10. Bhutta ZA, Das JK, Bahl R, et al. Can available interventions end preventable deaths in mothers, newborn babies, and stillbirths, and at what cost? *The Lancet*. 2014 Jul 26;384(9940):347-70. doi: 10.1016/S0140-6736(14)60792-3.

For peer review only

11. Leslie HH, Sun Z, Kruk ME. Association between infrastructure and observed quality of care in 4 healthcare services: A cross-sectional study of 4,300 facilities in 8 countries. *PLoS Med.* 2017 Dec 12;14(12):e1002464. doi: 10.1371/journal.pmed.1002464.

12. Thaddeus S, Maine D. Too far to walk: Maternal mortality in context. Soc Sci Med. 1994 Apr 1;38(8):1091–110.

13. WHO | WHO Safe Childbirth Checklist [Internet]. WHO. [access date 2018 May 3]. Available from: http://www.who.int/patientsafety/implementation/checklists/childbirth/en/

14. Perry WRG, Nejad SB, Tuomisto K, et al. Implementing the WHO Safe Childbirth Checklist: lessons from a global collaboration. *BMJ Glob Health*. 2017 Aug 19;2(3):e000241. doi: 10.1136/bmjgh-2016-000241.

15. Haynes AB, Weiser TG, Berry WR, et al. A Surgical Safety Checklist to Reduce Morbidity and Mortality in a Global Population. N Engl J Med. 2009 Jan 29;360(5):491-9. doi: 10.1056/NEJMsa0810119.

16. Arriaga AF, Bader AM, Wong JM, et al. Simulation-Based Trial of Surgical-Crisis Checklists. *N Engl J Med.* 2013 Jan 17;368(3):246-53. doi: 10.1056/NEJMsa1204720.

17. Munabi-Babigumira S, Glenton C, Lewin S, et al. Factors that influence the provision of intrapartum and postnatal care by skilled birth attendants in low- and middle-income countries: a qualitative evidence synthesis. *Cochrane Database Syst Rev.* 2017 Nov 17;11(11):CD011558. doi: 10.1002/14651858.CD011558.pub2.

18. Knight HE, Self A, Kennedy SH. Why are women dying when they reach hospital on time? A systematic review of the "third delay." *PLoS One.* 2013 May 21;8(5):e63846. doi: 10.1371/journal.pone.0063846.

19. Semrau KEA, Hirschhorn LR, Marx Delaney M, et al. Outcomes of a Coaching-Based WHO Safe Childbirth Checklist Program in India. *N Engl J Med.* 2017 Dec 14;377(24):2313-2324. doi: 10.1056/NEJMoa1701075.

20. Semrau KEA, Hirschhorn LR, Kodkany B, et al. Effectiveness of the WHO Safe Childbirth Checklist program in reducing severe maternal, fetal, and newborn harm in Uttar Pradesh, India: study protocol for a matched-pair, cluster-randomized controlled trial. *Trials.* 2016 Dec 7;17(1):576. doi: 10.1186/s13063-016-1673-x.

21. Tsai W-H. A technical note on using work sampling to estimate the effort on activities under activity-based costing. *Int J Prod Econ.* 1996 May 1;43(1):11–6. https://doi.org/10.1016/0925-5273(95)00189-1.

22. Wallwiener S, Müller M, Doster A, et al. Predictors of impaired breastfeeding initiation and maintenance in a diverse sample: what is important? *Arch Gynecol Obstet.* 2016 Sep;294(3):455-66. doi: 10.1007/s00404-015-3994-5.

For peer review only - http://bmjopen.bmj.com/site/about/guidelines.xhtml

23. Colin WB, Scott JA. Breastfeeding: reasons for starting, reasons for stopping and problems along the way. *Breastfeed Rev.* 2002 Jul;10(2):13-9

24. Centers for Disease Control and Prevention. Guideline for Hand Hygiene in Health-Care Settings: Recommendations of the Healthcare Infection Control Practices Advisory Committee and the HICPAC/SHEA/APIC/IDSA Hand Hygiene Task Force. MMWR 2002;51

25. Austin A, Langer A, Salam RA et al. Approaches to improve the quality of maternal and newborn health care: an overview of the evidence. *Reprod Health*. 2014 Sep 4;11 Suppl 2(Suppl 2):S1. doi: 10.1186/1742-4755-11-S2-S1.

26. Bryant AS, Worjoloh A, Caughey AB, et al. Racial/ethnic disparities in obstetric outcomes and care: prevalence and determinants. *Am J Obstet Gynecol.* 2010 Apr;202(4):335-43. doi: 10.1016/j.ajog.2009.10.864.

27. Mann S, Hollier LM, McKay K, et al. What We Can Do about Maternal Mortality — And How to Do It Quickly. *N Engl J Med.* 2018 Nov 1;379(18):1689-1691. doi: 10.1056/NEJMp1810649.

28. Koblinsky M, Moyer CA, Calvert C, et al. Quality maternity care for every woman, everywhere: a call to action. *The Lancet.* 2016 Nov 5;388(10057):2307-2320. doi: 10.1016/S0140-6736(16)31333-2.

29. Bustreo F, Say L, Koblinsky M, et al. Ending preventable maternal deaths: the time is now. *Lancet Glob Health.* 2013 Oct;1(4):e176-7. doi: 10.1016/S2214-109X(13)70059-7.

FIGURE 1
Title: Time-to-complete specific Checklist related tasks*
Legend: Tasks with 2 or fewer observations have been excluded from this graph but are included in Appendix Table A6

FIGURE 2
Title: Birth attendant tasks stratified by patient-load per provider
Legend: colored bar regions represent the interquartile range.

FIGURE 3
Title: Percent of facility-staff hours recorded as downtime by patient-load per birth attendant
Legend: none

For peer review only

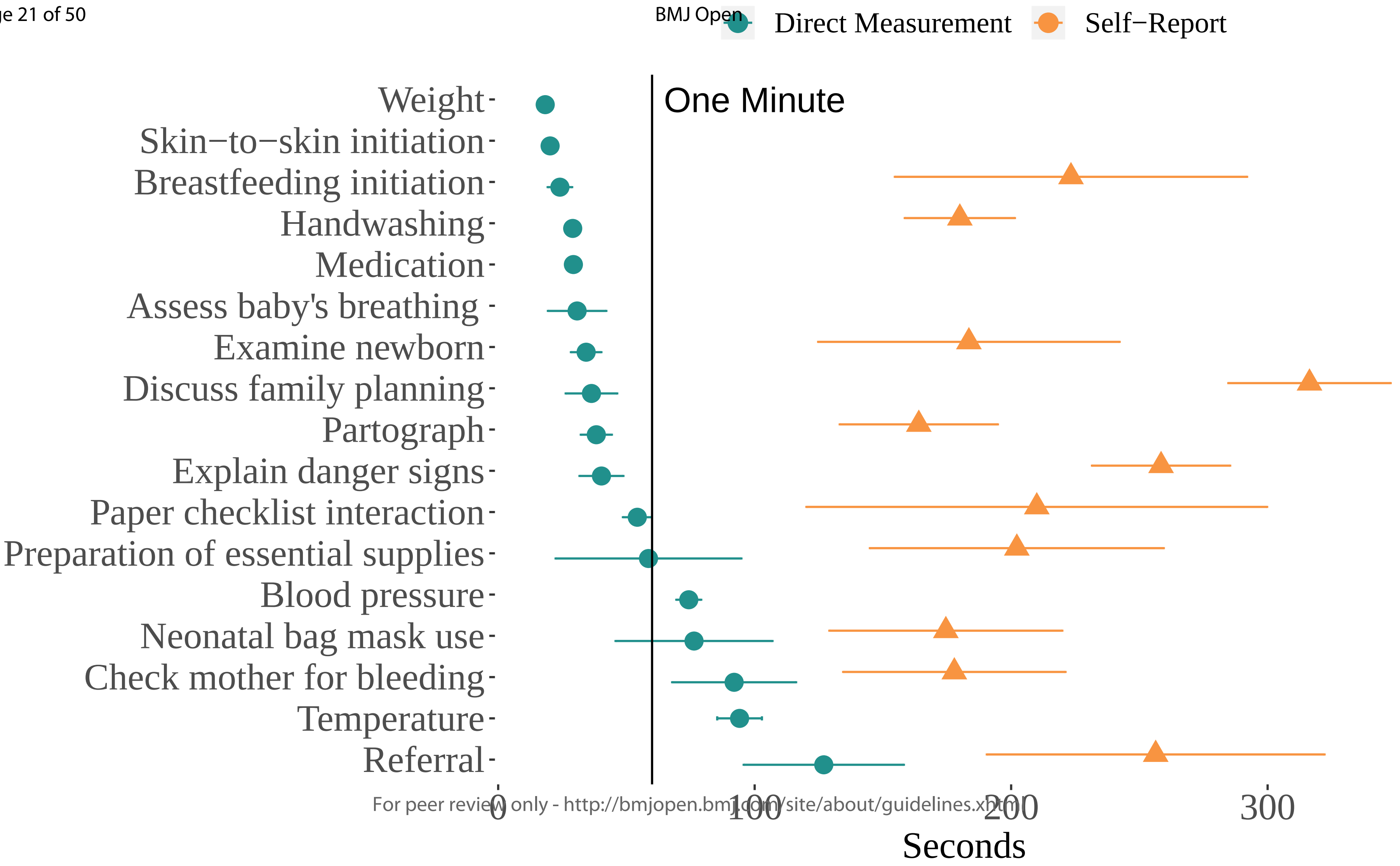
Direct Measurement
Self−Report
One Minute
Weight
Skin−to−skin initiation
Breastfeeding initiation
Handwashing
Medication
Assess baby's breathing
Examine newborn
Discuss family planning
Partograph
Explain danger signs
Paper checklist interaction
Preparation of essential supplies
Blood pressure
Neonatal bag mask use
Check mother for bleeding
Temperature
Referral
0
100
200
300
Seconds

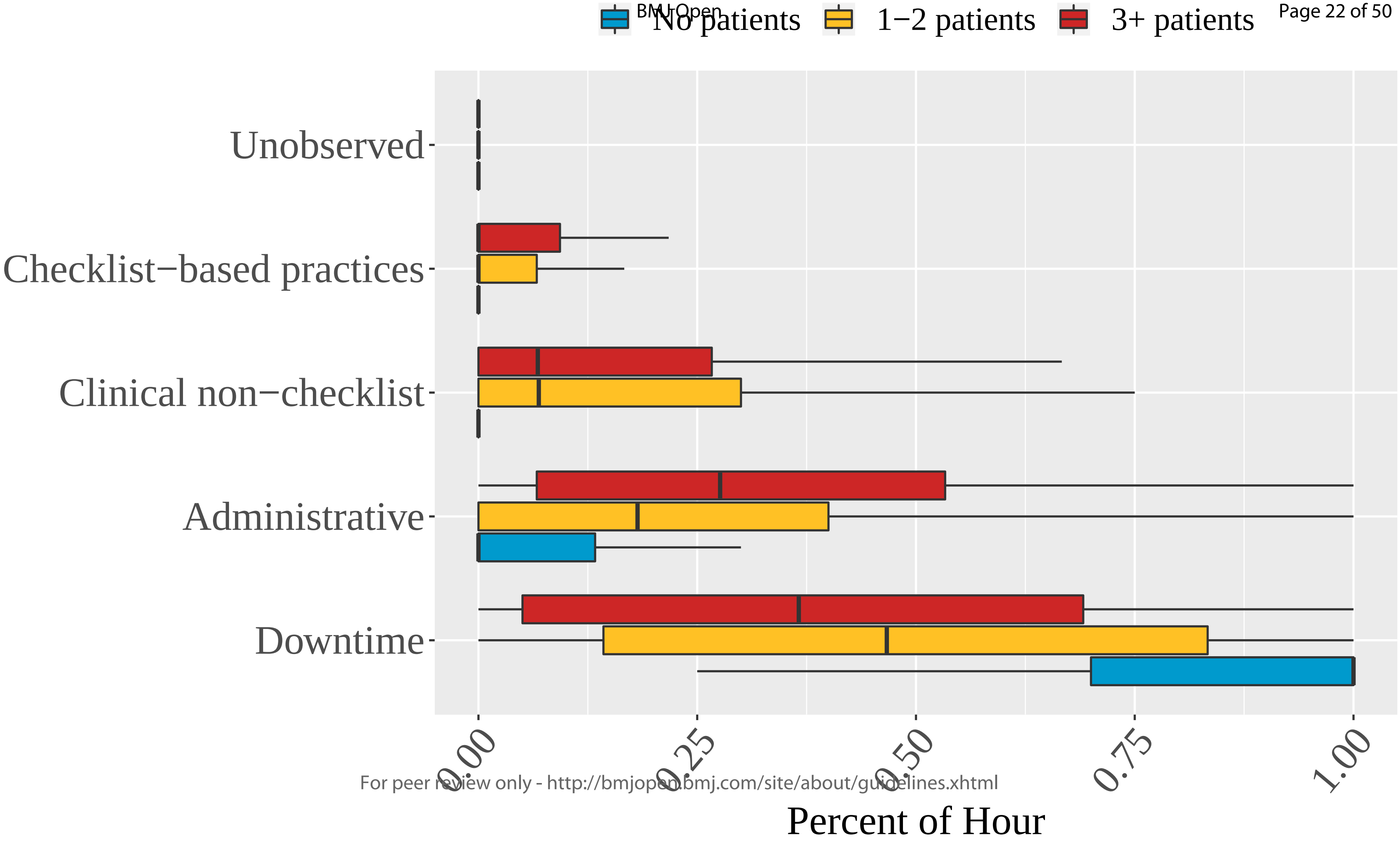
No patients
1−2 patients
3+ patients
Unobserved
Checklist−based practices
Clinical non−checklist
Administrative
Downtime
0.00
0.25
0.50
0.75
1.00
Percent of Hour

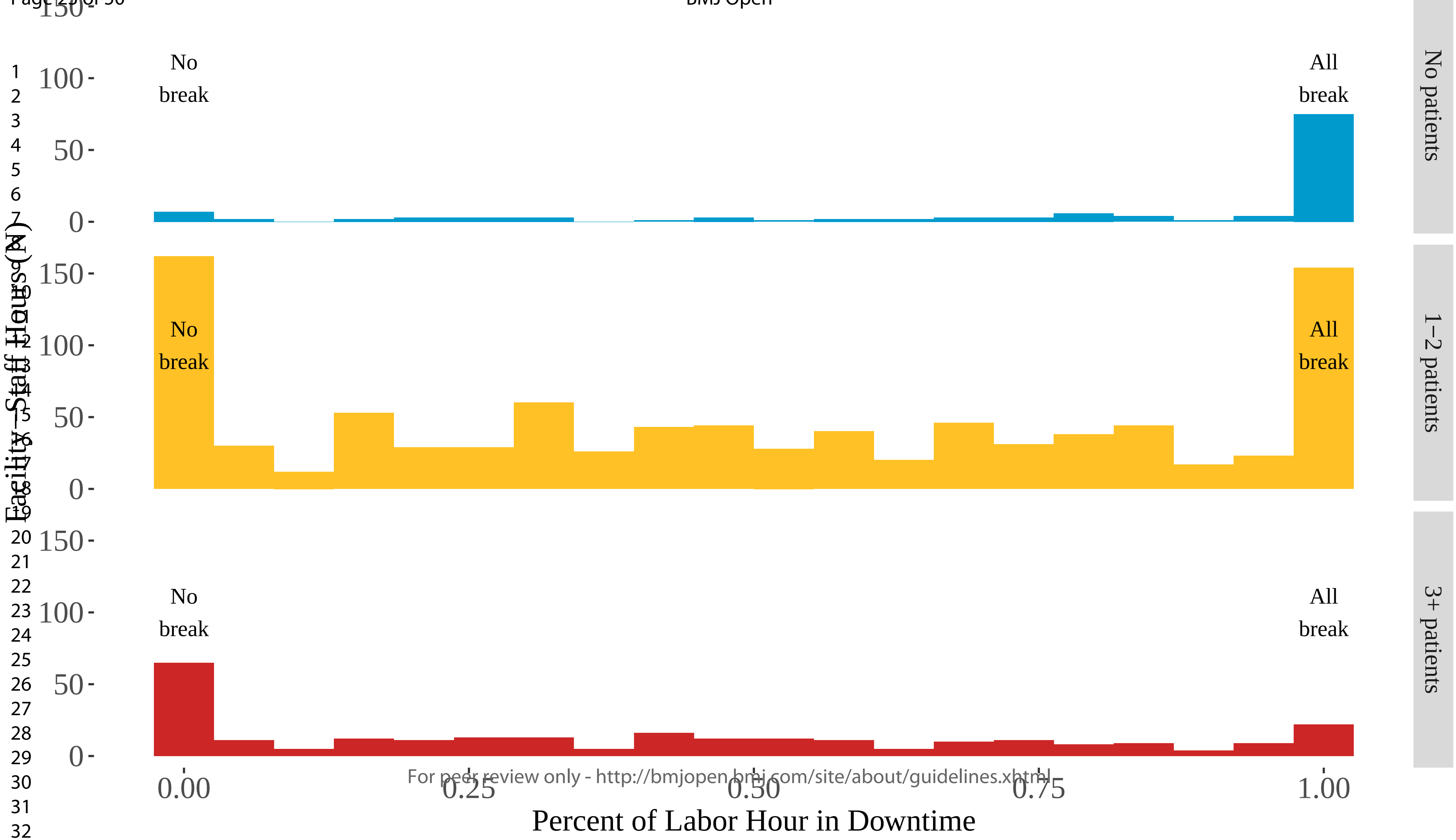
Facility - Staff Hours (N)
150
100
50
0
No
break
All
break
No patients
1–2 patients
3+ patients
0.00
0.25
0.50
0.75
1.00
Percent of Labor Hour in Downtime

Supplementary Appendix

Labor in labor and delivery wards: Evidence on provider time-use from the BetterBirth Trial

Appendix Table A1: Activities measured by data collection method

Master (18)	Work Sampling (16)	Time Motion (17)	Time Use (14)
Temperature	Temperature	Temperature	Temperature
Blood pressure	Blood pressure	Blood Pressure	N/A
Partograph	Partograph	Partograph	Partograph
Paper checklist interaction	Checklist/ poster	Paper checklist interaction	Checklist/ poster
Medication	Medication	Admin. Antibiotics/ Admin. Vaccines	Medication
Handwashing	Handwash gloves or alcohol rub	Handwashing	Handwash gloves or alcohol rub
Preparation of essential supplies	Prep of EBS	Prep of essential supplies	Prep of EBS
Neonatal bag mask	Use neonatal bag mask	Neonatal bag	Use neonatal bag mask
Referral	Referring a patient	Referral	Referring a patient
Check mother for bleeding	Check Mother for bleeding	Check mother for bleeding	Check mother for bleeding
Examine newborn	Examination of Newborn (BA)<br>Examination of Newborn (ASHA)	Examine newborn<br>Examine newborn for danger signs	Examination of newborn<br>N/A
Skin-to-skin initiation	Initiation of skin-to-skin	Init. of skin-to-skin	N/A
Discuss family planning	Discussing family planning<br>Group discussion	Discuss family planning<br>Discuss family planning (Group)	Discussing family planning<br>N/A
Explain danger signs	Explaining danger signs	Explain danger signs	Explaining danger signs
Breastfeeding initiation	Initiation of breastfeeding	Init. of breastfeeding	Initiation of breastfeeding
Confirm vaccination	Confirmation of vaccination	N/A	Confirmation of vaccination
Weight	N/A	Weight	N/A
Check baby's breathing	N/A	Assess Baby's Breathing	N/A

For peer review only

For peer review only - http://bmjopen.bmj.com/site/about/guidelines.xhtml

Appendix Table A2: Time Motion Observation Tool
(see next page for start of PDF)

For peer review only

For peer review only - http://bmjopen.bmj.com/site/about/guidelines.xhtml

Time Motion Observation Tool

A	Facility Code	
B	Date of Observation (DD/MMM/YYYY)	____/____/____
C	Health Care Worker Unique ID	
D	Health Care Worker Cadre	☐ Doctor ☐ L.H.V ☐ A.N.M ☐ Staff Nurse ☐ Other
E	Years of experience as a Health Care Worker	________Years _______Months
F	Years of experience as a Health Care Worker at this health facility	________Years _______Months
G	Did Health Care Worker consent to Observation	___Yes ___No
H	Notes about Time Motion observations:	
I	Tool ID Code	
J	FADA Employee ID	
K	FADA Role	☐ First Observer ☐ DQA Observer
L	FADA Start Time	______:______
M	FADA End Time	______:______

	Patient Yearly Number___________<br><br>From which register did you get the yearly number?<br>❑ Delivery<br>❑ Referred Out<br>❑ Admission				Patient Yearly Number _____________<br><br>From which register did you get the yearly number?<br>❑ Delivery<br>❑ Referred Out<br>❑ Admission				Patient Yearly Number __________<br><br>From which register did you get the yearly number?<br>❑ Delivery<br>❑ Referred Out<br>❑ Admission				General Activity (cannot be assigned to specific patient)
	PP 1	PP 2	PP 3	PP 4	PP 1	PP 2	PP 3	PP 4	PP 1	PP 2	PP 3	PP 4	
**Maternal Temperature**													
**Blood Pressure**													
**Partograph interaction**													
**Paper checklist or poster interaction**													
**Administration of antibiotics, magnesium sulfate, oxytocin or antiretroviral**													
**Hand washing, clean gloves or alcohol rub**													
**Preparation of Essential Supplies at bedside table**													
**Use of neonatal bag and mask for baby**													
**Referring a patient**													

	**Patient Yearly Number___________**<br><br>**From which register did you get the yearly number?**<br>❑ **Delivery**<br>❑ **Referred Out**<br>❑ **Admission**				**Patient Yearly Number _____________**<br><br>**From which register did you get the yearly number?**<br>❑ **Delivery**<br>❑ **Referred Out**<br>❑ **Admission**				**Patient Yearly Number __________**<br><br>**From which register did you get the yearly number?**<br>❑ **Delivery**<br>❑ **Referred Out**<br>❑ **Admission**				**General Activity (cannot be assigned to specific patient)**
	PP 1	PP 2	PP 3	PP 4	PP 1	PP 2	PP 3	PP 4	PP 1	PP 2	PP 3	PP 4	
**Check mother for bleeding**													
**Examination of Newborn**													
Examine the Baby for Danger Signs													
Assess Baby's Breathing													
Take Baby's Temperature													
Take Baby's Weight													
Monitor the Baby in order to Take Appropriate Action for Special Care (Requires Resuscitation)													
**Initiation of skin-to-skin**													

	Patient Yearly Number___________<br><br>From which register did you get the yearly number?<br>❑ Delivery<br>❑ Referred Out<br>❑ Admission				Patient Yearly Number _____________<br><br>From which register did you get the yearly number?<br>❑ Delivery<br>❑ Referred Out<br>❑ Admission				Patient Yearly Number __________<br><br>From which register did you get the yearly number?<br>❑ Delivery<br>❑ Referred Out<br>❑ Admission				General Activity (cannot be assigned to specific patient)
	PP 1	PP 2	PP 3	PP 4	PP 1	PP 2	PP 3	PP 4	PP 1	PP 2	PP 3	PP 4	
**Discussing Family Planning**													
**Explaining Danger Signs for mother and child**													
Group Discussion (if family planning and danger signs could not be observed)													
**Initiation of breastfeeding**													
**Administration of Vaccination**													

	Patient Yearly Number___________<br>From which register did you get the yearly number?<br>❑ Delivery<br>❑ Referred Out<br>❑ Admission				Patient Yearly Number _____________<br>From which register did you get the yearly number?<br>❑ Delivery<br>❑ Referred Out<br>❑ Admission				Patient Yearly Number __________<br>From which register did you get the yearly number?<br>❑ Delivery<br>❑ Referred Out<br>❑ Admission				General Activity (cannot be assigned to specific patient)
	PP 1	PP 2	PP 3	PP 4	PP 1	PP 2	PP 3	PP 4	PP 1	PP 2	PP 3	PP 4	
**Maternal Temperature**													
**Blood Pressure**													
**Partograph interaction**													
**Paper checklist or poster interaction**													
**Administration of antibiotics, magnesium sulfate, oxytocin or antiretroviral**													
**Hand washing, clean gloves or alcohol rub**													
**Preparation of Essential Supplies at bedside table**													
**Use of neonatal bag and mask for baby**													
**Referring a patient**													

	Patient Yearly Number___________<br><br>From which register did you get the yearly number?<br>❑ Delivery<br>❑ Referred Out<br>❑ Admission				Patient Yearly Number _____________<br><br>From which register did you get the yearly number?<br>❑ Delivery<br>❑ Referred Out<br>❑ Admission				Patient Yearly Number __________<br><br>From which register did you get the yearly number?<br>❑ Delivery<br>❑ Referred Out<br>❑ Admission				General Activity (cannot be assigned to specific patient)
	PP 1	PP 2	PP 3	PP 4	PP 1	PP 2	PP 3	PP 4	PP 1	PP 2	PP 3	PP 4	
**Check mother for bleeding**													
**Examination of Newborn**													
Examine the Baby for Danger Signs													
Assess Baby's Breathing													
Take Baby's Temperature													
Take Baby's Weight													
Monitor the Baby in order to Take Appropriate Action for Special Care (Requires Resuscitation)													
**Initiation of skin-to-skin**													

	Patient Yearly Number___________<br><br>From which register did you get the yearly number?<br>❑ Delivery<br>❑ Referred Out<br>❑ Admission				Patient Yearly Number _____________<br><br>From which register did you get the yearly number?<br>❑ Delivery<br>❑ Referred Out<br>❑ Admission				Patient Yearly Number __________<br><br>From which register did you get the yearly number?<br>❑ Delivery<br>❑ Referred Out<br>❑ Admission				General Activity (cannot be assigned to specific patient)
	PP 1	PP 2	PP 3	PP 4	PP 1	PP 2	PP 3	PP 4	PP 1	PP 2	PP 3	PP 4	
**Discussing Family Planning**													
**Explaining Danger Signs for mother and child**													
Group Discussion (if family planning and danger signs could not be observed)													
**Initiation of breastfeeding**													
**Administration of Vaccination**													

Appendix Table A3: Work Sampling Census
(see next page for start of PDF)

For peer review only

For peer review only - http://bmjopen.bmj.com/site/about/guidelines.xhtml

BB Work Sampling Census Sheet

Cover Page

A	Facility Code	
B	Date of Observation (DD/MMM/YYYY)	______/______/______
C	Notes about Work Sampling Census Observation:	
D	FADA Employee ID	

Work Sampling Census Sheet 29April2016

BB Work Sampling Census Page

Directions: update these numbers when you begin work sampling and in the last 5 minutes of every 2 hours of observation. Record the time and update the numbers based on FADA TL observation only and not official record

Hour	Start	Start + 2	Start + 4	Start + 6	Start + 8	Start + 10				
Clock Time										
Number of Women Admitted During Previous 2 hours										
Number of Women CURRENTLY in Waiting Room										
Number of Women CURRENTLY in L&D										
Number of Women CURRENTLY in Recovery										
Number Women Discharged / Transferred / Died During Previous 2 hours										
Number of Birth Attendants CURRENTLY on Duty in L&D										
Number of Helpers CURRENTLY on Duty in L&D										

Work Sampling Census Sheet 29April2016

Appendix Table A4: Work Sampling Observation Tool
(see next page for start of PDF)

For peer review only

For peer review only - http://bmjopen.bmj.com/site/about/guidelines.xhtml

BB Work Sampling Observation Tool

A	Facility Code	
B	Date of Observation (DD/MMM/YYYY)	____/____/____
C	Health Care Worker Unique ID	
D	Health Care Worker Cadre	□ Doctor □ L.H.V □ A.N.M □ Staff Nurse □ Other
E	Years of Experience as a Health Worker	________ Years ________ Months
F	Years of Experience as a Health Worker at this health facility	
G	Did Health care Worker consent to Observation	___Yes ___No
H	Notes about Work Sampling Observation:	
I	Tool ID Code	
J	FADA Employee ID	
K	FADA Role	□ First Observer □ DQA Observer
L	FADA Start Time	______:______
M	FADA End Time	______:______

Patients Consented	
Yearly Number	From which register did you get the yearly number?

BB Work Sampling Tool 10Jun2016

For peer review only - http://bmjopen.bmj.com/site/about/guidelines.xhtml

WHO Safe Childbirth Checklist Activities																		Non WHO SCC Activities				
Obser-vation Time	1. Mat Temp	2. Blood Pressure	3. Parto.	4. Checklist/ poster	5. Medication	6. Hand-wash gloves or alochol rub	7. Prep of EBS	8. Use neonatal bag mask	9. Referring a patient	10. Check Mother for bleeding	11. Exami-nation of Newborn	12. Initiation of skin-to-skin	13. Discuss-ing Family Planning	14. Danger signs	15. Group Discussion (if family planning and danger signs could not be observed)	16. Initiation of Breast-feeding	17. Confirm-ation of Vaccination	18. Non-CL Direct Patient Care	19. Admin. Duties	20. Break	21. Down-time	22. Unob served
HOUR 1																						
:02																						
:04																						
:06																						
:08																						
:10																						
:12																						
:14																						
:16																						
:18																						
:20																						
:22																						
:24																						
:26																						
:28																						
:30																						
:32																						
:34																						
:36																						
:38																						
:40																						
:42																						
:44																						
:46																						
:48																						
:50																						
:52																						
:54																						
:56																						
:58																						

Notes:

For peer review only

Observation Time	1. Mat Temp	2. Blood Pressure	3. Parto.	4. Checklist/ poster	5. Medication	6. Hand-wash gloves or alochol rub	7. Prep of EBS	8. Use neonatal bag mask	9. Referring a patient	10. Check Mother for bleeding	11. Examination of Newborn	12. Initiation of skin-to-skin	13. Discussing Family Planning	14. Danger signs	15. Group Discussion (if family planning and danger signs could not be observed)	16. Initiation of Breast-feeding	17. Confirmation of Vaccination	18. Non-CL Direct Patient Care	19. Admin. Duties	20. Break	21. Downtime	22. Unobserved
HOUR 2																						
:00																						
:02																						
:04																						
:06																						
:08																						
:10																						
:12																						
:14																						
:16																						
:18																						
:20																						
:22																						
:24																						
:26																						
:28																						
:30																						
:32																						
:34																						
:36																						
:38																						
:40																						
:42																						
:44																						
:46																						
:48																						
:50																						
:52																						
:54																						
:56																						
:58																						

Notes:

For peer review only

Observation Time	1. Mat Temp	2. Blood Pressure	3. Parto.	4. Checklist/ poster	5. Medication	6. Hand-wash gloves or alochol rub	7. Prep of EBS	8. Use neonatal bag mask	9. Referring a patient	10. Check Mother for bleeding	11. Examination of Newborn	12. Initiation of skin-to-skin	13. Discussing Family Planning	14. Danger signs	15. Group Discussion (if family planning and danger signs could not be observed)	16. Initiation of Breast-feeding	17. Confirmation of Vaccination	18. Non-CL Direct Patient Care	19. Admin. Duties	20. Break	21. Down-time	22. Unobserved
HOUR 3																						
:00																						
:02																						
:04																						
:06																						
:08																						
:10																						
:12																						
:14																						
:16																						
:18																						
:20																						
:22																						
:24																						
:26																						
:28																						
:30																						
:32																						
:34																						
:36																						
:38																						
:40																						
:42																						
:44																						
:46																						
:48																						
:50																						
:52																						
:54																						
:56																						
:58																						

Notes:

For peer review only

Observation Time	1. Mat Temp	2. Blood Pressure	3. Parto.	4. Checklist/ poster	5. Medication	6. Hand-wash gloves or alochol rub	7. Prep of EBS	8. Use neonatal bag mask	9. Referring a patient	10. Check Mother for bleeding	11. Examination of Newborn	12. Initiation of skin-to-skin	13. Discussing Family Planning	14. Danger signs	15. Group Discussion (if family planning and danger signs could not be observed)	16. Initiation of Breast-feeding	17. Confirmation of Vaccination	18. Non-CL Direct Patient Care	19. Admin. Duties	20. Break	21. Down-time	22. Unobserved
HOUR 4																						
:00																						
:02																						
:04																						
:06																						
:08																						
:10																						
:12																						
:14																						
:16																						
:18																						
:20																						
:22																						
:24																						
:26																						
:28																						
:30																						
:32																						
:34																						
:36																						
:38																						
:40																						
:42																						
:44																						
:46																						
:48																						
:50																						
:52																						
:54																						
:56																						
:58																						

Notes:

For peer review only

For peer review only - http://bmjopen.bmj.com/site/about/guidelines.xhtml

Obser-vation Time	1. Mat Temp	2. Blood Pressure	3. Parto.	4. Checklist/ poster	5. Medication	6. Hand-wash gloves or alochol rub	7. Prep of EBS	8. Use neonatal bag mask	9. Referring a patient	10. Check Mother for bleeding	11. Exami-nation of Newborn	12. Initiation of skin-to-skin	13. Discuss-ing Family Planning	14. Danger signs	15. Group Discussion (if family planning and danger signs could not be observed)	16. Initiation of Breast-feeding	17. Confirm-ation of Vaccination	18. Non-CL Direct Patient Care	19. Admin. Duties	20. Break	21. Down-time	22. Unob-served
HOUR 5																						
:00																						
:02																						
:04																						
:06																						
:08																						
:10																						
:12																						
:14																						
:16																						
:18																						
:20																						
:22																						
:24																						
:26																						
:28																						
:30																						
:32																						
:34																						
:36																						
:38																						
:40																						
:42																						
:44																						
:46																						
:48																						
:50																						
:52																						
:54																						
:56																						
:58																						

Notes:

For peer review only

Obser-vation Time	1. Mat Temp	2. Blood Pressure	3. Parto.	4. Checklist/ poster	5. Medication	6. Hand-wash gloves or alochol rub	7. Prep of EBS	8. Use neonatal bag mask	9. Referring a patient	10. Check Mother for bleeding	11. Exami-nation of Newborn	12. Initiation of skin-to-skin	13. Discuss-ing Family Planning	14. Danger signs	15. Group Discussion (if family planning and danger signs could not be observed)	16. Initiation of Breast-feeding	17. Confirm-ation of Vaccination	18. Non-CL Direct Patient Care	19. Admin. Duties	20. Break	21. Down-time	22. Unob-served
HOUR 6																						
:00																						
:02																						
:04																						
:06																						
:08																						
:10																						
:12																						
:14																						
:16																						
:18																						
:20																						
:22																						
:24																						
:26																						
:28																						
:30																						
:32																						
:34																						
:36																						
:38																						
:40																						
:42																						
:44																						
:46																						
:48																						
:50																						
:52																						
:54																						
:56																						
:58																						

Notes:

For peer review only

Observation Time	1. Mat Temp	2. Blood Pressure	3. Parto.	4. Checklist/ poster	5. Medication	6. Hand-wash gloves or alochol rub	7. Prep of EBS	8. Use neonatal bag mask	9. Referring a patient	10. Check Mother for bleeding	11. Examination of Newborn	12. Initiation of skin-to-skin	13. Discussing Family Planning	14. Danger signs	15. Group Discussion (if family planning and danger signs could not be observed)	16. Initiation of Breast-feeding	17. Confirmation of Vaccination	18. Non-CL Direct Patient Care	19. Admin. Duties	20. Break	21. Down-time	22. Unobserved
HOUR 7																						
:00																						
:02																						
:04																						
:06																						
:08																						
:10																						
:12																						
:14																						
:16																						
:18																						
:20																						
:22																						
:24																						
:26																						
:28																						
:30																						
:32																						
:34																						
:36																						
:38																						
:40																						
:42																						
:44																						
:46																						
:48																						
:50																						
:52																						
:54																						
:56																						
:58																						

Notes:

For peer review only

Obser-vation Time	1. Mat Temp	2. Blood Pressure	3. Parto.	4. Checklist/ poster	5. Medication	6. Hand-wash gloves or alochol rub	7. Prep of EBS	8. Use neonatal bag mask	9. Referring a patient	10. Check Mother for bleeding	11. Exami-nation of Newborn	12. Initiation of skin-to-skin	13. Discuss-ing Family Planning	14. Danger signs	15. Group Discussion (if family planning and danger signs could not be observed)	16. Initiation of Breast-feeding	17. Confirm-ation of Vaccination	18. Non-CL Direct Patient Care	19. Admin. Duties	20. Break	21. Down-time	22. Unob-served
HOUR 8																						
:00																						
:02																						
:04																						
:06																						
:08																						
:10																						
:12																						
:14																						
:16																						
:18																						
:20																						
:22																						
:24																						
:26																						
:28																						
:30																						
:32																						
:34																						
:36																						
:38																						
:40																						
:42																						
:44																						
:46																						
:48																						
:50																						
:52																						
:54																						
:56																						
:58																						

Notes:

For peer review only

Appendix Table A5: General task categories in work sampling

General Group	Specific Activity
Checklist (CL)	Temperature<br>Blood pressure<br>Partograph<br>Paper checklist interaction<br>Medication<br>Handwashing<br>Prep of essential supplies<br>Neonatal bag mask<br>Referral<br>Check mother for bleeding<br>Examine newborn<br>Skin-to-skin initiation<br>Discuss family planning<br>Explain danger signs<br>Breastfeeding initiation<br>Confirm vaccination
Non-Checklist Clinical	Non-CL Direct Patient Care
Administrative	Admin. Duties
Downtime	Break<br>Downtime

For peer review only

Appendix Table A6: Task-time estimates for Essential Birth Practices

Essential Birth Practices	Time Source	Mean	SE	Min (seconds)	Max	Sample Size
Referral	Direct measurement	127	16	16	330	21
	Self-report	256	34	90	720	22
Temperature	Direct measurement	94	4	3	275	92
	Self-report	60	0	60	60	2
Check mother for bleeding	Direct measurement	92	12	2	320	37
	Self-report	178	22	60	300	14
Neonatal bag mask use	Direct measurement	76	16	6	300	22
	Self-report	175	23	60	300	11
Blood pressure	Direct measurement	74	2	20	165	126
	Self-report			N/A		N/A
Preparation of essential supplies	Direct measurement	59	18	8	436	31
	Self-report	202	29	120	600	18
Paper checklist interaction	Direct measurement	54	3	4	180	175
	Self-report	210	46	150	300	3
Explain danger signs	Direct measurement	40	4	8	109	41
	Self-report	258	14	60	600	65
Partograph	Direct measurement	38	3	9	88	38
	Self-report	164	16	30	240	15
Discuss family planning	Direct measurement	36	5	4	94	30
	Self-report	316	16	60	600	60
Examine newborn	Direct measurement	34	3	4	177	143
	Self-report	184	30	60	600	17
Assess baby's breathing	Direct measurement	31	6	10	60	9
	Self-report			N/A		N/A
Medication	Direct measurement	29	1	1	150	419
	Self-report	225	75	150	300	2
Handwashing	Direct measurement	29	1	1	81	208
	Self-report	180	11	150	210	6
Breastfeeding initiation	Direct measurement	24	2	6	60	43
	Self-report	223	35	120	420	9
Skin-to-skin initiation	Direct measurement	20	2	3	93	89
	Self-report			N/A		N/A
Weight	Direct measurement	18	2	5	44	35
	Self-report			N/A		N/A

Appendix Table A7: Heat map of self-reported most time-consuming Checklist tasks*

Checklist Activity	Rank 1 (Number of Respondents)	Rank 2	Rank 3	Total
Discussing family planning	41	16	3	60
Explaining danger signs	22	31	12	65
Prep of EBS	5	3	10	18
Check mother for bleeding	3	4	7	14
Initiation of breastfeeding	3	2	4	9
Referring a patient	3	11	8	22
Partograph	2	3	10	15
Use neonatal bag mask	2	1	8	11
Examination of newborn	1	4	12	17
Handwash gloves or alcohol rub	1	3	2	6
Checklist/ poster	0	2	1	3
Confirmation of vaccination	0	2	3	5
Medication	0	1	1	2
Temperature	0	0	2	2

*Number of staff reporting activity in each rank position; total staff interviewed = 83

For peer review only - http://bmjopen.bmj.com/site/about/guidelines.xhtml

Appendix Figure A1: Median labor and delivery ward patient load by hour (time of day) (1319 facility-hour observations; one facility-hour dropped in hour 20)

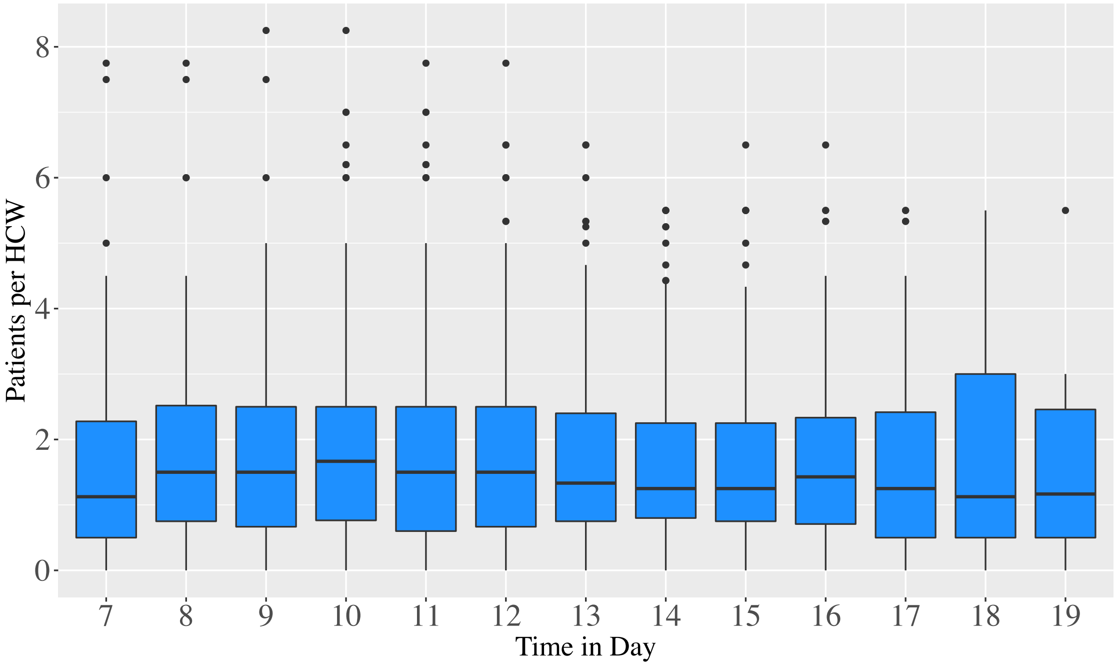

Appendix Figure A2: Median percent of hour on break by patient load per HCW (1320 facility-hour observations)

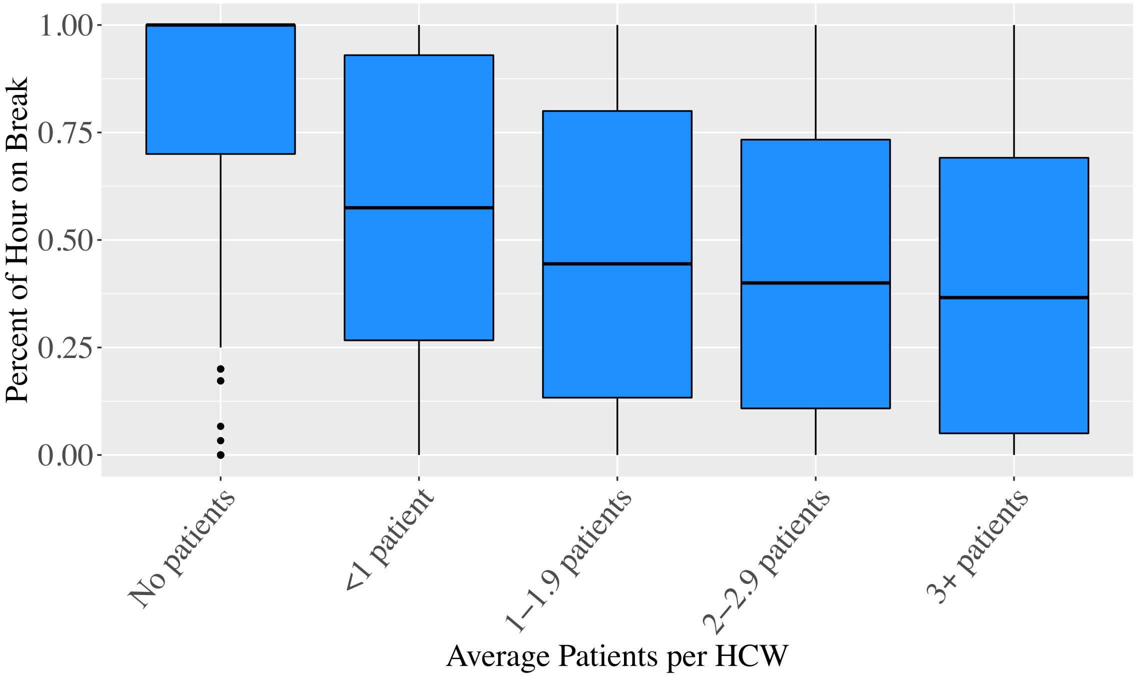

CONSORT 2010 checklist of information to include when reporting a randomised trial*

Section/Topic	Item No	Checklist item	Reported on page No
**Title and abstract**			
	1a	Identification as a randomised trial in the title	1
	1b	Structured summary of trial design, methods, results, and conclusions (for specific guidance see CONSORT for abstracts)	6
**Introduction**			
Background and objectives	2a	Scientific background and explanation of rationale	4-5
	2b	Specific objectives or hypotheses	6
**Methods**			
Trial design	3a	Description of trial design (such as parallel, factorial) including allocation ratio	6
	3b	Important changes to methods after trial commencement (such as eligibility criteria), with reasons	N/A
Participants	4a	Eligibility criteria for participants	6
	4b	Settings and locations where the data were collected	6
Interventions	5	The interventions for each group with sufficient details to allow replication, including how and when they were actually administered	6-8
Outcomes	6a	Completely defined pre-specified primary and secondary outcome measures, including how and when they were assessed	10-12
	6b	Any changes to trial outcomes after the trial commenced, with reasons	N/A
Sample size	7a	How sample size was determined	See protocol paper
	7b	When applicable, explanation of any interim analyses and stopping guidelines	N/A
Randomisation:			
Sequence generation	8a	Method used to generate the random allocation sequence	See protocol paper
	8b	Type of randomisation; details of any restriction (such as blocking and block size)	6 + abstract
Allocation concealment mechanism	9	Mechanism used to implement the random allocation sequence (such as sequentially numbered containers), describing any steps taken to conceal the sequence until interventions were assigned	See protocol paper
Implementation	10	Who generated the random allocation sequence, who enrolled participants, and who assigned participants to	See protocol

For peer review only - http://bmjopen.bmj.com/site/about/guidelines.xhtml
For peer review only

		interventions	paper
Blinding	11a	If done, who was blinded after assignment to interventions (for example, participants, care providers, those assessing outcomes) and how	N/A
	11b	If relevant, description of the similarity of interventions	N/A
Statistical methods	12a	Statistical methods used to compare groups for primary and secondary outcomes	Descriptive analysis in RCT
	12b	Methods for additional analyses, such as subgroup analyses and adjusted analyses	N/A
**Results**			
Participant flow (a diagram is strongly recommended)	13a	For each group, the numbers of participants who were randomly assigned, received intended treatment, and were analysed for the primary outcome	6 and 10-12
	13b	For each group, losses and exclusions after randomisation, together with reasons	N/A
Recruitment	14a	Dates defining the periods of recruitment and follow-up	7
	14b	Why the trial ended or was stopped	7
Baseline data	15	A table showing baseline demographic and clinical characteristics for each group	See main RCT results paper
Numbers analysed	16	For each group, number of participants (denominator) included in each analysis and whether the analysis was by original assigned groups	See main RCT results paper
Outcomes and estimation	17a	For each primary and secondary outcome, results for each group, and the estimated effect size and its precision (such as 95% confidence interval)	See main RCT results paper
	17b	For binary outcomes, presentation of both absolute and relative effect sizes is recommended	N/A
Ancillary analyses	18	Results of any other analyses performed, including subgroup analyses and adjusted analyses, distinguishing pre-specified from exploratory	All pre-specified measures
Harms	19	All important harms or unintended effects in each group (for specific guidance see CONSORT for harms)	N/A
**Discussion**			
Limitations	20	Trial limitations, addressing sources of potential bias, imprecision, and, if relevant, multiplicity of analyses	12-15
Generalisability	21	Generalisability (external validity, applicability) of the trial findings	12-15
Interpretation	22	Interpretation consistent with results, balancing benefits and harms, and considering other relevant evidence	12-15

For peer review only

**Other information**			
Registration	23	Registration number and name of trial registry	In submission fields
Protocol	24	Where the full trial protocol can be accessed, if available	Published manuscript included in submission
Funding	25	Sources of funding and other support (such as supply of drugs), role of funders	In submission fields

*We strongly recommend reading this statement in conjunction with the CONSORT 2010 Explanation and Elaboration for important clarifications on all the items. If relevant, we also recommend reading CONSORT extensions for cluster randomised trials, non-inferiority and equivalence trials, non-pharmacological treatments, herbal interventions, and pragmatic trials. Additional extensions are forthcoming: for those and for up to date references relevant to this checklist, see www.consort-statement.org.

For peer review only

For peer review only - http://bmjopen.bmj.com/site/about/guidelines.xhtml

BMJ Open

Estimating maternity ward birth attendant time use in India: A microcosting study

Journal:	*BMJ Open*
Manuscript ID	bmjopen-2021-054164.R2
Article Type:	Original research
Date Submitted by the Author:	30-Dec-2021
Complete List of Authors:	Lofgren, Katherine; Harvard University, Interfaculty Initiative in Health Policy<br>Bobanski , Lauren ; Ariadne Labs, Harvard T.H. Chan School of Public Health/Brigham and Women’s Hospital, 401 Park Drive, 3rd floor West<br>Tuller, Danielle; Harvard TH Chan School of Public Health / Brigham and Women’s Hospital<br>Singh, Vinay P.; Population Services International; Community Empowerment Lab<br>Marx Delaney , Megan; Ariadne Labs; Harvard T. H. Chan School of Public Health<br>Jurczak, Amanda; Ariadne Labs<br>Ragavan, Meera; University of California San Francisco, Department of Medicine, Division of Hematology/Oncology<br>Kalita, Tapan; Piramal Swasthya Management and Research Institute<br>Karlage, Ami; Ariadne Labs<br>Resch, Stephen; Harvard University T H Chan School of Public Health, Center for Health Decision Science<br>Semrau, Katherine; Brigham and Women's Hospital, Ariadne Labs; Brigham and Women's Hospital, Division of Global Health Equity & Department of Medicine
**Primary Subject Heading**:	Global health
Secondary Subject Heading:	Obstetrics and gynaecology
Keywords:	Health economics < HEALTH SERVICES ADMINISTRATION & MANAGEMENT, Maternal medicine < OBSTETRICS, Quality in health care < HEALTH SERVICES ADMINISTRATION & MANAGEMENT

SCHOLARONE™
Manuscripts

I, the Submitting Author has the right to grant and does grant on behalf of all authors of the Work (as defined in the below author licence), an exclusive licence and/or a non-exclusive licence for contributions from authors who are: i) UK Crown employees; ii) where BMJ has agreed a CC-BY licence shall apply, and/or iii) in accordance with the terms applicable for US Federal Government officers or employees acting as part of their official duties; on a worldwide, perpetual, irrevocable, royalty-free basis to BMJ Publishing Group Ltd ("BMJ") its licensees and where the relevant Journal is co-owned by BMJ to the co-owners of the Journal, to publish the Work in this journal and any other BMJ products and to exploit all rights, as set out in our licence.

The Submitting Author accepts and understands that any supply made under these terms is made by BMJ to the Submitting Author unless you are acting as an employee on behalf of your employer or a postgraduate student of an affiliated institution which is paying any applicable article publishing charge ("APC") for Open Access articles. Where the Submitting Author wishes to make the Work available on an Open Access basis (and intends to pay the relevant APC), the terms of reuse of such Open Access shall be governed by a Creative Commons licence – details of these licences and which Creative Commons licence will apply to this Work are set out in our licence referred to above.

Other than as permitted in any relevant BMJ Author's Self Archiving Policies, I confirm this Work has not been accepted for publication elsewhere, is not being considered for publication elsewhere and does not duplicate material already published. I confirm all authors consent to publication of this Work and authorise the granting of this licence.

For peer review only

Title: **Estimating maternity ward birth attendant time use in India: A microcosting study**

Corresponding Author: **Katherine T. Lofgren**
Harvard University, Interfaculty Initiative in Health Policy
14 Story St.
Cambridge, MA. 02138
kate.lofgren@gmail.com

Co-Authors:

Lauren Bobanski
Ariadne Labs, Brigham & Women's Hospital and Harvard T.H. Chan School of Public Health, Boston, MA., USA
Danielle E. Tuller
Ariadne Labs, Brigham & Women's Hospital and Harvard T.H. Chan School of Public Health, Boston, MA., USA
Vinay P. Singh
Population Services International, Lucknow, India
Community Empowerment Lab, Lucknow, India
Megan Marx Delaney
Ariadne Labs, Brigham & Women's Hospital and Harvard T.H. Chan School of Public Health, Boston, MA., USA
Amanda Jurczak
Ariadne Labs, Brigham & Women's Hospital and Harvard T.H. Chan School of Public Health, Boston, MA., USA
Meera Ragavan
University of California San Francisco, Department of Medicine, Division of Hematology/Oncology, San Francisco, CA.
Tapan Kalita
Piramal Swasthya Management and Research Institute, Hyderabad, India
Ami Karlage
Ariadne Labs, Brigham & Women's Hospital and Harvard T.H. Chan School of Public Health, Boston, MA., USA
Stephen Resch
Harvard Center for Health Decision Sciences, Harvard T.H. Chan School of Public Health, Boston, MA. USA
Katherine E. A. Semrau
Ariadne Labs, Brigham & Women's Hospital and Harvard T.H. Chan School of Public Health, Boston, MA., USA
Department of Medicine, Harvard Medical School, Boston, MA. USA
Division of Global Health Equity, Brigham & Women's Hospital, Boston, MA. USA

Word Count: 3294

For peer review only

Abstract:

Objectives

Despite global concern over the quality of maternal care, little is known about the time requirements to complete essential birth practices. Using three micro-costing data collection methods within the BetterBirth trial, we aimed to assess time use and the specific time requirements to incorporate the WHO Safe Childbirth Checklist into clinical practice.

Setting

We collected detailed survey data on birth attendant time-use within the BetterBirth trial in Uttar Pradesh, India. The BetterBirth trial tested whether the peer-coaching-based implementation of the WHO Checklist was effective in improving the quality of facility-based childbirth care.

Participants

We collected measurements of time-to-completion for 18 essential birth practices from July 2016 through October 2016 across 10 facilities in 5 districts (1559 total timed observations). An anonymous survey asked about the impact of the WHO Checklist on birth attendants at every intervention facility (15 facilities, 83 respondents) in the Lucknow hub. Additionally, data collectors visited facilities to conduct a census of patients and birth attendants across 20 facilities in 7 districts between June 2016 to November 2016 (610 2-hour facility observations).

Primary and secondary outcome measures

The primary outcome measure of this study is the percent of staff time required to complete the essential birth practices included in the WHO Checklist.

Results

When birth attendants were timed, we found practices were completed rapidly (18 seconds – 2 minutes). As the patient load increased, time dedicated to clinical care increased but remained low relative to administrative and downtime. On average, WHO Checklist clinical care accounted for less than 7% of birth attendant time-use per hour.

Conclusions

We did not find that a coaching-based implementation of the WHO Checklist was a burden on birth attendant's time-use. However, questions remain regarding the performance quality of practices and how to accurately capture and interpret idle and break time.

Trial registration

NCT02148952

For peer review only - http://bmjopen.bmj.com/site/about/guidelines.xhtml

Strengths and Limitations

- Three distinct time-use capture methods were used to estimate the time-requirements of a coaching-based implementation of the World Health Organization (WHO) Safe Childbirth Checklist
- Both stop-watch and birth attendant reported time-to-complete individual evidence-based practices were used to estimate time-to-complete essential birth practices
- A census of birth attendants and patients in combination with work sampling data on birth attendant time use was used to estimate the percent of a staffing hour spent on general task categories including administrative duties, Checklist-based practices, Clinical non-Checklist, and downtime.
- As this work was embedded within the BetterBirth trial, only a subset of treatment facilities were sampled and we are not able to compare across treatment and control facilities or conduct sub-analyses by facility type
- Further work is needed to differentiate true downtime from watchful waiting as this study does not include breakdowns within the broad category of downtime.

For peer review only

INTRODUCTION

Remarkable achievements have been made in the reduction of maternal and neonatal mortality globally.[1,2] One of the primary achievements of the Millennium Development Goals Era was increased rate of facility-based childbirth.[3] However, evidence suggests increased coverage of services does not necessarily lead to mortality and morbidity reductions.[4,5] A large portion of stillbirths and maternal and neonatal deaths remain preventable with timely, high-quality care.[6–8] As low- and middle-income countries (LMICs) continue to expand access to services, ensuring patients receive high-quality, evidence-based clinical care is essential for continued progress in population health.[9]

The use of evidence-based care in labor and delivery facilities remains low.[10,11] Even when women reach a facility in a timely manner, without adequate and appropriate treatment, preventable deaths occur.[12] Many interventions seek to improve the quality of care in LMIC health systems by increasing the number of essential birth practices performed for each laboring mother.[9] The World Health Organization's Safe Childbirth Checklist (Checklist) is one such effort.[13] The Checklist is a clinical care aid that synthesizes and prioritizes evidence-based essential birth practices (practices) from admission to discharge in order to increase the number of practices—like handwashing, checking the mother for bleeding, or discussing family planning—performed by birth attendants (BAs) at the point of care. Defining essential practices and creating mechanisms like the Checklist for clinical staff to consistently implement those practices has been successful across a diverse set of clinical contexts in both high- and low-income settings.[14–16]

For peer review only - http://bmjopen.bmj.com/site/about/guidelines.xhtml

One complicating factor in quality improvement efforts targeting labor and delivery wards specifically is staff time availability. Across health systems, BAs often report feeling overwhelmed and busy.[17] Additionally, staffing shortages are a known barrier to timely, high-quality clinical care.[18] With any quality-improvement intervention, clinical care may increase staffing time demands or replace existing low-value activities. The implementation of quality-improvement interventions requires understanding existing staff time capacity at baseline and how staff time-use changes post-implementation.

The BetterBirth trial was a matched-pair, cluster-randomized, controlled trial of a coaching-based implementation of the Checklist in Uttar-Pradesh, India to test the effect of the intervention on a composite outcome of perinatal mortality, maternal mortality, or maternal severe complication within 7 days of giving birth.[19,20] Embedded in the BetterBirth trial, we conducted data collection to measure the time-demands of the Checklist practices, with the primary intent of informing a cost-effectiveness analysis (CEA) of the BetterBirth trial. When the main outcome of the BetterBirth trial was a null effect on maternal and neonatal mortality and morbidity, the CEA was rendered irrelevant. However, concerns remained about the possibility that the Checklist introduced a significant time burden on BAs. Prior to the implementation of the BetterBirth trial, BAs often reported feeling overwhelmed and busy. As a result, we used the collected data to answer the following questions:

(1) What is the time-burden of the practices included in the Checklist?

(2) Do BAs perceive the Checklist as a significant stress- or time-burden?

(3) How does BA time-use change as their patient load increases?

For peer review only - http://bmjopen.bmj.com/site/about/guidelines.xhtml

METHODS

Study setting

The BetterBirth trial was a peer-coaching-based implementation of the Checklist in Uttar Pradesh, India. The matched-pair, cluster-randomized, controlled trial randomized the BetterBirth trial across 120 facilities (60 control, 60 treatment) with a study population of women and their newborns, the birth attendants (BAs) providing care.. Study facilities had more than 1,000 deliveries per year and minimum of four labor and delivery staff. The study protocol and results have been published and include further details on the study population, design, and methods used to test the primary outcome of interest, maternal and perinatal mortality and morbidity outcomes.[19,20]

This paper details three time-use data collection methods to triangulate the time-burden of the Checklist practices within the broader time-demands on BAs within the BetterBirth trial.[21] Data collectors (N=16) were junior nurses who received training and supportive supervision for data quality assurance across all three data collection methods (each described in more detail in subsections below). We captured 18 specific Checklist practices (Appendix Table A1) as well as non-Checklist clinical care, administrative duties, and break/downtime. Although the intention was to distinguish between a scheduled break and non-scheduled downtime, efforts to delineate between these two activities by data collectors was difficult in practice. For the purposes of this paper, 'downtime' refers to a mix of scheduled breaks as well as idle time for other reasons, such as no patients or watchful waiting during clinical care. We first measured time-to-completion for 18 practices via direct BA observation during clinical practice (time-demand). We then surveyed BAs about their experience during the BetterBirth trial (perceived time-demand). Finally, we

For peer review only - http://bmjopen.bmj.com/site/about/guidelines.xhtml

visited facilities and conducted both a census of births as well as observing clinical care activities at regular intervals (BA time-use).

The time-demand of Checklist practices

We collected measurements of Checklist practice time-to-completion for 18 practices over a four-month period from July 2016 through October 2016 across 10 facilities in 5 districts. Data collectors visited each facility 2-3 times per week, for 8 hours shifts between 7am-3pm or 11am-7pm. If available, a second data collector or a supervisor performed data quality assurance activities. Time-to-complete tasks were assessed by the data collectors with stopwatches, recorded on paper (Appendix Table A2), and transferred to an Excel spreadsheet. The time measurements were used to estimate the time required to complete each Checklist practice.

Perceived time-demand of the Checklist by BAs

We also surveyed BAs on their time-burden perceptions. The anonymous survey asked general questions about the impact of the Checklist on the daily routines and workloads of BAs (83 respondents) at every intervention facility (15 facilities) in the Lucknow hub (the cost-effectiveness data collection survey region with 30 total facilities) from June to July 2016. All staff working at the facility on the day of data collection were provided the survey and could answer anonymously. The survey also asked respondents to rank the top three most time-consuming items on the Checklist and estimate the time required to complete those tasks. The specific time estimates for Checklist practices were used to supplement and compare with the stopwatch time measurements.

BA time-use in the labor and delivery ward

For peer review only - http://bmjopen.bmj.com/site/about/guidelines.xhtml

How providers use their time depends on both the patient demand and the number of BAs on duty. To estimate the patient demand and health care labor supply, data collectors visited facilities to conduct a census of patients and BAs every 2 hours, recording the results on a paper form (Appendix Table A3). Observations were taken across 20 facilities in 7 districts from June 2016 to November 2016. This data was used to calculate the average number of patients per BA at given facilities and times of day.

In addition to the census, we also observed BAs conducting regular care (a work sampling approach) to capture the proportion of time spent on various types of clinical and non-clinical work.[22] A data collector visited a facility and, for each hour observed, recorded the type of activity the BA was engaged in at pre-specified 2-minute intervals on a paper form (Appendix Table A4). For example, if at 11:00 a.m. the BA was using a neonatal bag and mask, the data collector recorded that activity. At 11:02 a.m. the data collector would again record what the BA was doing; in some cases, she might still be using a neonatal bag and mask, while in other cases, there may be a new activity listed such as non-Checklist direct patient care. This type of data provides estimates of proportional time spent on various activities but does not directly estimate the time required for specific tasks.

If there were at least two BAs on duty at the same time, observations alternated between two BAs. For example, the 11:00 AM observation would pertain to BA1 while the 11:02 AM observation would pertain to BA2, alternating back and forth throughout the hour. If only one BA was available for observation, an observation was taken every 2 minutes for their work. We calculate the proportion of each BA's time spent in different general activity categories to

For peer review only - http://bmjopen.bmj.com/site/about/guidelines.xhtml

estimate the overall time-use in given facility-hours (Appendix Table A5 maps specific activities to general categories).

Ethics approval

The study protocol was approved by the ethics committees of: Community Empowerment Lab (Ref no: 2014006), Jawaharlal Nehru Medical College (Ref no: MDC/IECHSR/2015–16/A-53), Harvard T.H. Chan School of Public Health (Protocol 21 975–102), Population Services International (Protocol ID: 47·2012), WHO (Protocol ID: RPC 501) and Indian Council of Medical Research. The protocol was reviewed and re-approved on an annual basis. We obtained consent from each facility's leadership for trial participation and data collection on eligible mothers from facility registers. Birth attendants and facility staff verbally agreed to participate prior to trial initiation. Independent observers obtained written consent from women or their surrogates and verbal consent from birth attendants prior to observation.

Public involvement in research

Patient and provider representatives worked with us to refine the Checklist when it was originally designed in 2009. The BetterBirth trial study research question and design did not have direct patient involvement, but did have a scientific advisory committee that included clinicians, researchers, government officials who work in the same area. We did receive and modified the dissemination plan based on feedback from providers and government partners for each participating facility/district. Further, we published a report for wider dissemination and audience found at: betterbirth.ariadnelabs.org

For peer review only - http://bmjopen.bmj.com/site/about/guidelines.xhtml

RESULTS

The time-demand of Checklist practices

Across all Checklist practices, a total of 1,559 practices were directly timed from 35 unique birth attendants (BAs) across 10 facilities during clinical care (see Appendix Table A6 for practice-specific sample sizes). Handwashing (N=419) and the administration of medication (N=208) were the most frequently observed direct measurements, while referrals (N=21) and the assessment of the baby's breathing (N=9) had the fewest recorded observations. Directly measured task-times revealed a pattern of rapid time-to-complete practices on the Checklist. When Checklist practices were directly measured using stopwatches, the average time-to-complete the task ranged from 127 seconds (a referral) to 18 seconds (weighing the baby). Tasks like breastfeeding initiation and discussing family planning that require conversations and (potentially) complex patient-BA interactions both took less than 1 minute on average (black dots on Figure 1 and Appendix Table A6). Over 70% (N=12 out of 18 practices) of the average time-to-complete measurements were less than one minute.

Perceived time-demand of the Checklist by BAs

Across 15 facilities, there were 83 total respondents to the survey. The majority of BAs responded that the Checklist made their jobs easier (96%; N=80). When BAs were asked if the Checklist took away from non-Checklist activities, only 17% of responders felt other clinical duties were rushed (N=11) or their workday was prolonged (N=3).

For peer review only - http://bmjopen.bmj.com/site/about/guidelines.xhtml

Respondents were asked to rank the three most time-consuming Checklist practices and estimate the time required to complete those three tasks. Discussing family planning was the most frequently reported time-consuming activity (ranked #1 by 49% of BAs (Appendix Table A7)). All tasks were estimated by BAs to take less than 5:07 minutes on average. The self-reported task-times were longer than the direct measurements, particularly in discussion-based practices like explaining danger signs and discussion family planning (Figure 1).

BA time-use in the labor and delivery ward

BA time-use incorporates data from the work sampling time-use data collection and the facility BA and patient census. In total, 610 2-hour facility periods were recorded for the patient census and 27,768 individual task observations were recorded in our work sampling survey. Within the hours of data collection (7am-7pm), we found relatively constant median patient-load at 1.4 patients-per-BA with large variability in the potential patient-load for any given facility-hour (0 to 8 patients-per-BA observed range) (Appendix Figure A1).

Clinical care (both non-Checklist and Checklist) was 21% of the average facility-staff hour. As patients-per-BA increased, so did clinical care and administrative duties. When there were no patients, BAs spent the majority of their time in downtime (80% of time) or conducting administrative tasks (15% of time) and less than 1% of time on Checklist clinical care. Once the patient-load increased to 1-2 patients-per-BA, BA time-use shifted towards clinical care (23% of BA time; 5% Checklist specific) as well as administrative tasks (26% up from 15% with no patients). At 3 or more patients-per-BA, the Checklist accounted for 7% of BA time (out of a total 24% of the hour spent on clinical care). However, even at high patient-loads (3+), the most

For peer review only - http://bmjopen.bmj.com/site/about/guidelines.xhtml

common time-use, on average, was still recorded as downtime (40% in downtime compared to 24% in clinical care; Figure 2). Sample size breakdowns for individual work sampling observations by patient load and task-type are available in Appendix Table A8.

However, the average BA downtime is a misleading statistic. When the full distribution of BA downtime by facility-BA-hour are graphed, there is clear heterogeneity in the distribution that is not captured by summary measures like the mean or median percent of the hour spent in downtime. Particularly for the 1-2 patient categorization, there is a clear bi-modal trend with BAs spending the majority of staff-hours either completely in downtime or without any downtime. Similarly, for the 0 and 3+ patient categories, the distributions are highly skewed to all downtime (0 patients) and no downtime (3+ patients). Taken across all these categories, summary statistics mask the extreme downtime dichotomy experienced in practice by BAs (Figure 3, Appendix Figure A2). Sample size breakdowns for individual work sampling observations by patient load and task-type are available in Appendix Table A9.

DISCUSSION

The time-demand on BAs is an important piece of the maternal and newborn quality-of-care puzzle. Quality improvement efforts inherently require staff time to shift away from existing time uses and towards evidence-based practices such as those included in the Checklist. Using three different data collection efforts, we found that the Checklist practices were not an undue time burden on BAs. However, based on our data, we are not confident that practices were performed at sufficient quality. Further, our results show a high proportion of ambiguously measured downtime, a lesson to learn from in future studies to differentiate watchful waiting

For peer review only - http://bmjopen.bmj.com/site/about/guidelines.xhtml

from true downtime. Concerns about the quality of care provided are consistent with the overall BetterBirth trial findings—treatment facilities did not have reduced mortality and morbidity after the Checklist was implemented.

Several of the time-to-complete practice measures seemed implausibly fast to be of sufficient quality. In particular, tasks like initiation of breastfeeding, initiation of skin-to-skin contact, discussion or family planning, and referrals likely require more time in expectation than is currently being allocated based on our study results. Referrals, which were the most time-consuming task overall, were still completed within 2 minutes. Given the difficulty of breastfeeding initiation,[23,24] it is unlikely that a mean task-time of 24 seconds (SE = 16 seconds) accurately captures the true time required to successfully initiate breastfeeding. In other cases, the timing seems plausible. For example, the CDC recommends handwashing for 15-20 seconds.[25] In our sample, handwashing took an average of 29 seconds. Similarly, although the self-reported sample of task-time estimates is skewed towards tasks that the BAs perceived as relatively more burdensome, the self-reported time-to-complete tasks remained lower than expected *a priori*. These results are similarly indicative that time-to-complete Checklist-related practices are too low to have been consistently performed at high-quality. Further research is needed to estimate minimum time requirements for the performance of practices at high quality and how the Checklist, when implemented at high-quality, impacts the staffing needs of a facility. One potential downside of a checklist-based intervention is a desire to get through the items as quickly as possible rather than at the pace required to perform each task at high-quality. Further quality approaches may also consider how to incorporate incentives for not just completing a checklist but reaching quantifiable quality benchmarks for the checklist items.

For peer review only - http://bmjopen.bmj.com/site/about/guidelines.xhtml

Although individual practices took less than 2 minutes to complete on average and overall less than 5 minutes for the full practice list, it is still possible that the workload of clinical care (and/or administrative tasks) before the introduction of the Checklist was sufficiently demanding that BAs did not have the slack to take on any incremental tasks newly introduced with the Checklist. Across all our data collection methods, however, high-quality clinical care was not the major time-use of BAs in the BetterBirth study population. One of the main open questions from our time-use data collection is how to understand and estimate the time-constraints faced by labor and delivery ward BAs. The nature of labor and delivery ward care requires long periods of waiting followed by high-stress, high-demand moments of clinical care. Could moments of inactivity actually be high-stress, high-alert contexts compared to times when the BA is truly on break? How should we differentiate between breaks that are necessary versus time that could be reallocated towards high-quality clinical care? How would the percent of time spent conducting clinical care change if quality of care improved? Our data highlights the importance and difficulty of estimating supply-side constraints in the highly unpredictable context of labor and delivery wards. In the future, it will be important to continue to estimate how quality improvement interventions impact the time-use of providers including work to parse out time which appears to be free, but in reality may be a version of alert waiting.

Ensuring quality care at facilities not only requires thoughtful clinical care practices, it also requires staffing strategies.[26–30] Our data collection efforts add empiric evidence on how BAs in Uttar Pradesh, India use their time across both clinical and non-clinical care under varying levels of patient demand. In future implementations of the Checklist, our data on the time-to-

For peer review only - http://bmjopen.bmj.com/site/about/guidelines.xhtml

complete clinical tasks as well as the time-use of BAs can serve as both a model of how to collect data and as a baseline for potential data collection improvements that could address lingering questions raised in this paper.

There are several limitations in our methods and data collection. Although we began with separate categories for breaks and downtime, this distinction was not clear during the actual observation. We cannot reliably distinguish true breaks from watchful waiting. Practices were meant to be timed from start to finish, pausing for breaks. For instance, if a family planning discussion began but was interrupted by breastfeeding initiation, the stopwatch should have been stopped and restarted when the family planning discussion restarted to capture the overall time required for that practice. Given the consistently short task-time estimates, this may not have occurred. In our survey of BAs, our sample size is relatively small (N=83), the responses may not generalize to the broader BA population in our study and Uttar Pradesh more broadly. Instead of asking the BAs to estimate the task-time for all 18 practices, we only asked for the top three in an effort to keep the survey short. However, it limits our self-reported task-times to only those activities that BAs considered especially time consuming, biasing the self-reported results upwards. Finally, this study was not designed to study variation in birth attendant time-use by facility-type, a stratified analysis by facility-type may help explain some of the variation in patient load per birth attendant and birth attendant time-use.

There are often calls for measurable indicators of health care quality. In the recent Lancet Global Health Commission on High Quality Health Systems, many of the available quality-metrics rely on the proportion or number of evidence-based practices performed.[9] Although completion of

For peer review only - http://bmjopen.bmj.com/site/about/guidelines.xhtml

tasks is important, our evidence suggests simply performing evidence-based care does not itself ensure quality. This outcome mirrors the message that coverage of services does not equate to quality. When future quality-improvement and evidence-based care interventions are implemented, it will remain important to understand how the intervention fits within the broader responsibilities and time demands of BAs as well as estimating time demands by facility-type. Quality care requires essential care is completed at a satisfactory level beyond simple completion of tasks.

ACKNOWLEDGMENTS
We recognize the Governments of India and Uttar Pradesh for collaboration and support to conduct this trial in public health facilities. We thank the facility staff, women and newborns for their participation in the study. We are indebted to Sriya Srikrishnan, Ezinne Eze-Ajoku, Bharath Kumar, Krishna Kumar, the late Dr. Narender Sharma, and all the data collectors on the facility-based data associate team. We are grateful to the members of the trial's Scientific Advisory Committee who contributed crucial guidance to the development of this study protocol.

CONTRIBUTORSHIP STATEMENT
KEAS, SR, and KTL conceived of the study. KTL conducted the analysis. VPS and TK supervised field operations and reviewed survey instruments. KTL, AK, KEAS, and LB wrote the manuscript. AK provided extensive edits and manuscript support. LB, DET, MMD, and AJ provided operational support for the study. MR provided clinical inputs for the study. All authors (KEAS, LB, DET, VPS, MMD, AJ, MR, TK, AK, SR, and KTL) reviewed the manuscript and provided edits.

COMPETING INTERESTS
The authors have no competing interests to declare.

FUNDING
Bill and Melinda Gates Foundation (OPP1017378)

DATA SHARING
Data are available in a public, open access repository. Data are available on the Harvard Dataverse Platform under the BetterBirth Dataverse website. This can be found at: https://dataverse.harvard.edu/dataverse/BetterBirthData

For peer review only

REFERENCES

1. Hogan DR, Stevens GA, Hosseinpoor AR, et al. Monitoring universal health coverage within the Sustainable Development Goals: development and baseline data for an index of essential health services. *Lancet Glob Health* 2018 Feb;6(2):e152-e168. doi: 10.1016/S2214-109X(17)30472-2.

2. Lozano R, Wang H, Foreman KJ, et al. Progress towards Millennium Development Goals 4 and 5 on maternal and child mortality: an updated systematic analysis. *The Lancet*. 2011 Sep 24;378(9797):1139-65. doi: 10.1016/S0140-6736(11)61337-8.

3. United Nations Millennium Development Goals [Internet]. [access date 2018 Sep]. Available from: http://www.un.org/millenniumgoals/maternal.shtml

4. Ng M, Misra A, Diwan V, et al. An assessment of the impact of the JSY cash transfer program on maternal mortality reduction in Madhya Pradesh, India. *Glob Health Action*. 2014 Dec 3;7:24939. doi: 10.3402/gha.v7.24939.

5. Okeke, Edward N. and Amalavoyal V. Chari, Can Institutional Deliveries Reduce Newborn Mortality? Evidence from Rwanda. Santa Monica, CA: *RAND Corporation*, 2015. https://www.rand.org/pubs/working_papers/WR1072.html

6. Ronsmans C, Scott S, Qomariyah SN, et al. Professional assistance during birth and maternal mortality in two Indonesian districts. *Bull World Health Organ*. 2009 Jun;87(6):416-23. doi: 10.2471/blt.08.051581.

7. Lawn JE, Blencowe H, Pattinson R, et al. Stillbirths: Where? When? Why? How to make the data count? *The Lancet*. 2011 Apr 23;377(9775):1448-63. doi: 10.1016/S0140-6736(10)62187-3.

8. Kruk ME, Gage AD, Joseph NT, et al. Mortality due to low-quality health systems in the universal health coverage era: a systematic analysis of amenable deaths in 137 countries. *The Lancet*. 2018 Nov 17;392(10160):2203-2212. doi: 10.1016/S0140-6736(18)31668-4.

9. Kruk ME, Gage AD, Arsenault C, et al. High-quality health systems in the Sustainable Development Goals era: time for a revolution. *Lancet Glob Health*. 2018 Nov;6(11):e1196-e1252. doi: 10.1016/S2214-109X(18)30386-3.

10. Bhutta ZA, Das JK, Bahl R, et al. Can available interventions end preventable deaths in mothers, newborn babies, and stillbirths, and at what cost? *The Lancet*. 2014 Jul 26;384(9940):347-70. doi: 10.1016/S0140-6736(14)60792-3.

11. Leslie HH, Sun Z, Kruk ME. Association between infrastructure and observed quality of care in 4 healthcare services: A cross-sectional study of 4,300 facilities in 8 countries. *PLoS Med.* 2017 Dec 12;14(12):e1002464. doi: 10.1371/journal.pmed.1002464.

For peer review only - http://bmjopen.bmj.com/site/about/guidelines.xhtml

12. Thaddeus S, Maine D. Too far to walk: Maternal mortality in context. Soc Sci Med. 1994 Apr 1;38(8):1091–110.

13. WHO | WHO Safe Childbirth Checklist [Internet]. WHO. [access date 2018 May 3]. Available from: http://www.who.int/patientsafety/implementation/checklists/childbirth/en/

14. Perry WRG, Nejad SB, Tuomisto K, et al. Implementing the WHO Safe Childbirth Checklist: lessons from a global collaboration. *BMJ Glob Health*. 2017 Aug 19;2(3):e000241. doi: 10.1136/bmjgh-2016-000241.

15. Haynes AB, Weiser TG, Berry WR, et al. A Surgical Safety Checklist to Reduce Morbidity and Mortality in a Global Population. N Engl J Med. 2009 Jan 29;360(5):491-9. doi: 10.1056/NEJMsa0810119.

16. Arriaga AF, Bader AM, Wong JM, et al. Simulation-Based Trial of Surgical-Crisis Checklists. *N Engl J Med.* 2013 Jan 17;368(3):246-53. doi: 10.1056/NEJMsa1204720.

17. Munabi-Babigumira S, Glenton C, Lewin S, et al. Factors that influence the provision of intrapartum and postnatal care by skilled birth attendants in low- and middle-income countries: a qualitative evidence synthesis. *Cochrane Database Syst Rev.* 2017 Nov 17;11(11):CD011558. doi: 10.1002/14651858.CD011558.pub2.

18. Knight HE, Self A, Kennedy SH. Why are women dying when they reach hospital on time? A systematic review of the "third delay." *PLoS One.* 2013 May 21;8(5):e63846. doi: 10.1371/journal.pone.0063846.

19. Semrau KEA, Hirschhorn LR, Marx Delaney M, et al. Outcomes of a Coaching-Based WHO Safe Childbirth Checklist Program in India. *N Engl J Med.* 2017 Dec 14;377(24):2313-2324. doi: 10.1056/NEJMoa1701075.

20. Semrau KEA, Hirschhorn LR, Kodkany B, et al. Effectiveness of the WHO Safe Childbirth Checklist program in reducing severe maternal, fetal, and newborn harm in Uttar Pradesh, India: study protocol for a matched-pair, cluster-randomized controlled trial. *Trials.* 2016 Dec 7;17(1):576. doi: 10.1186/s13063-016-1673-x.

21. Finkler, SA, Knickman JR, Hendrickson G, et al. A Comparison of Work-Sampling and Time-and-Motion Techniques for Studies in Health Services Research. *HSR: Health Services Research*. 1993 Dec; 28(5):577-97

22. Tsai W-H. A technical note on using work sampling to estimate the effort on activities under activity-based costing. *Int J Prod Econ.* 1996 May 1;43(1):11–6. https://doi.org/10.1016/0925-5273(95)00189-1.

23. Wallwiener S, Müller M, Doster A, et al. Predictors of impaired breastfeeding initiation and maintenance in a diverse sample: what is important? *Arch Gynecol Obstet.* 2016 Sep;294(3):455-66. doi: 10.1007/s00404-015-3994-5.

For peer review only - http://bmjopen.bmj.com/site/about/guidelines.xhtml

24. Colin WB, Scott JA. Breastfeeding: reasons for starting, reasons for stopping and problems along the way. *Breastfeed Rev.* 2002 Jul;10(2):13-9

25. Centers for Disease Control and Prevention. Guideline for Hand Hygiene in Health-Care Settings: Recommendations of the Healthcare Infection Control Practices Advisory Committee and the HICPAC/SHEA/APIC/IDSA Hand Hygiene Task Force. MMWR 2002;51

26. Austin A, Langer A, Salam RA et al. Approaches to improve the quality of maternal and newborn health care: an overview of the evidence. *Reprod Health*. 2014 Sep 4;11 Suppl 2(Suppl 2):S1. doi: 10.1186/1742-4755-11-S2-S1.

27. Bryant AS, Worjoloh A, Caughey AB, et al. Racial/ethnic disparities in obstetric outcomes and care: prevalence and determinants. *Am J Obstet Gynecol.* 2010 Apr;202(4):335-43. doi: 10.1016/j.ajog.2009.10.864.

28. Mann S, Hollier LM, McKay K, et al. What We Can Do about Maternal Mortality — And How to Do It Quickly. *N Engl J Med.* 2018 Nov 1;379(18):1689-1691. doi: 10.1056/NEJMp1810649.

29. Koblinsky M, Moyer CA, Calvert C, et al. Quality maternity care for every woman, everywhere: a call to action. *The Lancet*. 2016 Nov 5;388(10057):2307-2320. doi: 10.1016/S0140-6736(16)31333-2.

30. Bustreo F, Say L, Koblinsky M, et al. Ending preventable maternal deaths: the time is now. *Lancet Glob Health.* 2013 Oct;1(4):e176-7. doi: 10.1016/S2214-109X(13)70059-7.

FIGURE 1
Title: Time-to-complete specific Checklist related tasks*
Legend: Tasks with 2 or fewer observations have been excluded from this graph but are included in Appendix Table A6

FIGURE 2
Title: Birth attendant tasks stratified by patient-load per provider
Legend: colored bar regions represent the interquartile range.

FIGURE 3
Title: Percent of facility-staff hours recorded as downtime by patient-load per birth attendant
Legend: none

For peer review only

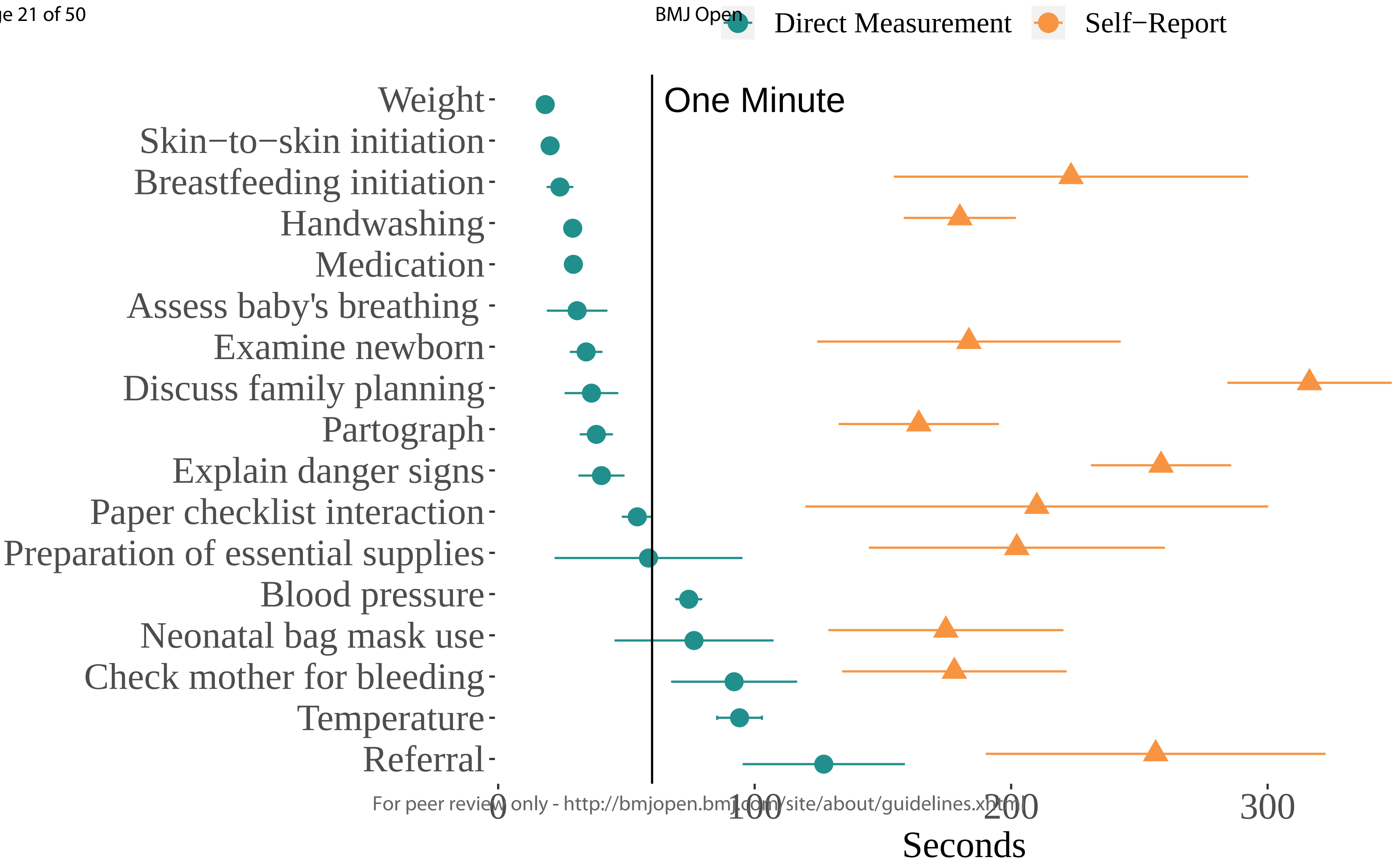
Direct Measurement
Self−Report
One Minute
Weight
Skin−to−skin initiation
Breastfeeding initiation
Handwashing
Medication
Assess baby's breathing
Examine newborn
Discuss family planning
Partograph
Explain danger signs
Paper checklist interaction
Preparation of essential supplies
Blood pressure
Neonatal bag mask use
Check mother for bleeding
Temperature
Referral
0
100
200
300
Seconds

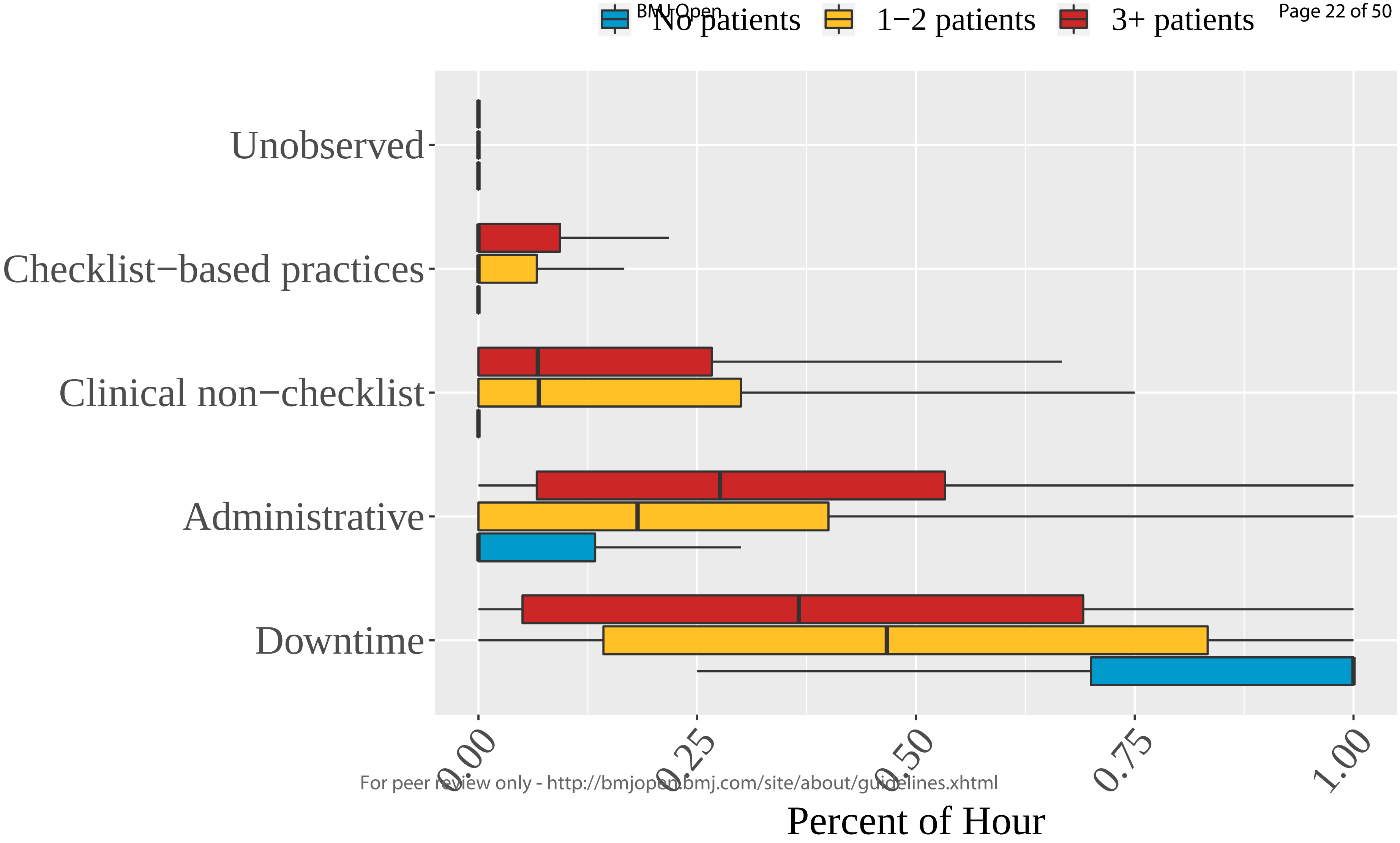
No patients
1−2 patients
3+ patients
Unobserved
Checklist−based practices
Clinical non−checklist
Administrative
Downtime
0.00
0.25
0.50
0.75
1.00
Percent of Hour

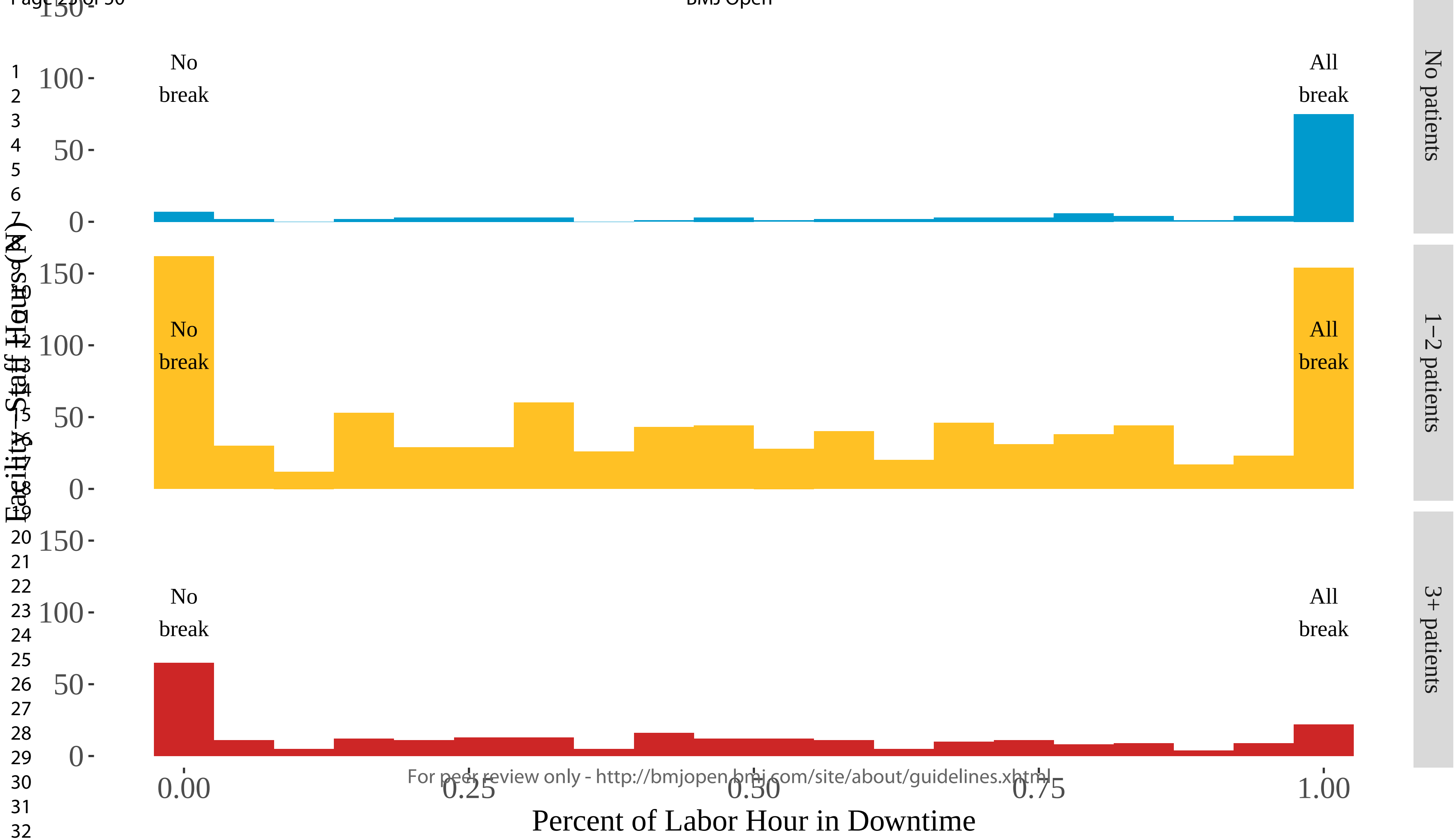
150
100
50
0
No
break
All
break
No patients
1–2 patients
3+ patients
Facility - Staff Hours (N)
0.00
0.25
0.50
0.75
1.00
Percent of Labor Hour in Downtime

Supplementary Appendix

Labor in labor and delivery wards:
Evidence on provider time-use from the BetterBirth Trial

Appendix Table A1: Activities measured by data collection method

Master (18)	Work Sampling (16)	Time Motion (17)	Time Use (14)
Temperature	Temperature	Temperature	Temperature
Blood pressure	Blood pressure	Blood Pressure	N/A
Partograph	Partograph	Partograph	Partograph
Paper checklist interaction	Checklist/ poster	Paper checklist interaction	Checklist/ poster
Medication	Medication	Admin. Antibiotics/ Admin. Vaccines	Medication
Handwashing	Handwash gloves or alcohol rub	Handwashing	Handwash gloves or alcohol rub
Preparation of essential supplies	Prep of EBS	Prep of essential supplies	Prep of EBS
Neonatal bag mask	Use neonatal bag mask	Neonatal bag	Use neonatal bag mask
Referral	Referring a patient	Referral	Referring a patient
Check mother for bleeding	Check Mother for bleeding	Check mother for bleeding	Check mother for bleeding
Examine newborn	Examination of Newborn (BA)<br>Examination of Newborn (ASHA)	Examine newborn<br>Examine newborn for danger signs	Examination of newborn<br>N/A
Skin-to-skin initiation	Initiation of skin-to-skin	Init. of skin-to-skin	N/A
Discuss family planning	Discussing family planning<br>Group discussion	Discuss family planning<br>Discuss family planning (Group)	Discussing family planning<br>N/A
Explain danger signs	Explaining danger signs	Explain danger signs	Explaining danger signs
Breastfeeding initiation	Initiation of breastfeeding	Init. of breastfeeding	Initiation of breastfeeding
Confirm vaccination	Confirmation of vaccination	N/A	Confirmation of vaccination
Weight	N/A	Weight	N/A
Check baby's breathing	N/A	Assess Baby's Breathing	N/A

For peer review only

Appendix Table A2: Time Motion Observation Tool
(see next page for start of PDF)

For peer review only

For peer review only - http://bmjopen.bmj.com/site/about/guidelines.xhtml

Time Motion Observation Tool

A	Facility Code	
B	Date of Observation (DD/MMM/YYYY)	____/____/____
C	Health Care Worker Unique ID	
D	Health Care Worker Cadre	☐ Doctor ☐ L.H.V ☐ A.N.M ☐ Staff Nurse ☐ Other
E	Years of experience as a Health Care Worker	________Years _______Months
F	Years of experience as a Health Care Worker at this health facility	________Years _______Months
G	Did Health Care Worker consent to Observation	___Yes ___No
H	Notes about Time Motion observations:	
I	Tool ID Code	
J	FADA Employee ID	
K	FADA Role	☐ First Observer ☐ DQA Observer
L	FADA Start Time	______:______
M	FADA End Time	______:______

	Patient Yearly Number___________<br><br>From which register did you get the yearly number?<br>❑ Delivery<br>❑ Referred Out<br>❑ Admission				Patient Yearly Number _____________<br><br>From which register did you get the yearly number?<br>❑ Delivery<br>❑ Referred Out<br>❑ Admission				Patient Yearly Number __________<br><br>From which register did you get the yearly number?<br>❑ Delivery<br>❑ Referred Out<br>❑ Admission				General Activity (cannot be assigned to specific patient)
	PP 1	PP 2	PP 3	PP 4	PP 1	PP 2	PP 3	PP 4	PP 1	PP 2	PP 3	PP 4	
**Maternal Temperature**													
**Blood Pressure**													
**Partograph interaction**													
**Paper checklist or poster interaction**													
**Administration of antibiotics, magnesium sulfate, oxytocin or antiretroviral**													
**Hand washing, clean gloves or alcohol rub**													
**Preparation of Essential Supplies at bedside table**													
**Use of neonatal bag and mask for baby**													
**Referring a patient**													

	Patient Yearly Number___________ From which register did you get the yearly number? ❑ Delivery ❑ Referred Out ❑ Admission				Patient Yearly Number _____________ From which register did you get the yearly number? ❑ Delivery ❑ Referred Out ❑ Admission				Patient Yearly Number __________ From which register did you get the yearly number? ❑ Delivery ❑ Referred Out ❑ Admission				General Activity (cannot be assigned to specific patient)
	PP 1	PP 2	PP 3	PP 4	PP 1	PP 2	PP 3	PP 4	PP 1	PP 2	PP 3	PP 4	
**Check mother for bleeding**													
**Examination of Newborn**													
Examine the Baby for Danger Signs													
Assess Baby's Breathing													
Take Baby's Temperature													
Take Baby's Weight													
Monitor the Baby in order to Take Appropriate Action for Special Care (Requires Resuscitation)													
**Initiation of skin-to-skin**													

	Patient Yearly Number____________<br><br>From which register did you get the yearly number?<br>❑ Delivery<br>❑ Referred Out<br>❑ Admission				Patient Yearly Number ______________<br><br>From which register did you get the yearly number?<br>❑ Delivery<br>❑ Referred Out<br>❑ Admission				Patient Yearly Number __________<br><br>From which register did you get the yearly number?<br>❑ Delivery<br>❑ Referred Out<br>❑ Admission				General Activity (cannot be assigned to specific patient)
	PP 1	PP 2	PP 3	PP 4	PP 1	PP 2	PP 3	PP 4	PP 1	PP 2	PP 3	PP 4	
**Discussing Family Planning**													
**Explaining Danger Signs for mother and child**													
Group Discussion (if family planning and danger signs could not be observed)													
**Initiation of breastfeeding**													
**Administration of Vaccination**													

	Patient Yearly Number____________<br><br>From which register did you get the yearly number?<br>❑ Delivery<br>❑ Referred Out<br>❑ Admission				Patient Yearly Number ______________<br><br>From which register did you get the yearly number?<br>❑ Delivery<br>❑ Referred Out<br>❑ Admission				Patient Yearly Number __________<br><br>From which register did you get the yearly number?<br>❑ Delivery<br>❑ Referred Out<br>❑ Admission				General Activity (cannot be assigned to specific patient)
	PP 1	PP 2	PP 3	PP 4	PP 1	PP 2	PP 3	PP 4	PP 1	PP 2	PP 3	PP 4	
**Maternal Temperature**													
**Blood Pressure**													
**Partograph interaction**													
**Paper checklist or poster interaction**													
**Administration of antibiotics, magnesium sulfate, oxytocin or antiretroviral**													
**Hand washing, clean gloves or alcohol rub**													
**Preparation of Essential Supplies at bedside table**													
**Use of neonatal bag and mask for baby**													
**Referring a patient**													

	Patient Yearly Number___________ From which register did you get the yearly number? ❑ Delivery ❑ Referred Out ❑ Admission				Patient Yearly Number _____________ From which register did you get the yearly number? ❑ Delivery ❑ Referred Out ❑ Admission				Patient Yearly Number __________ From which register did you get the yearly number? ❑ Delivery ❑ Referred Out ❑ Admission				General Activity (cannot be assigned to specific patient)
	PP 1	PP 2	PP 3	PP 4	PP 1	PP 2	PP 3	PP 4	PP 1	PP 2	PP 3	PP 4	
**Check mother for bleeding**													
**Examination of Newborn**													
Examine the Baby for Danger Signs													
Assess Baby's Breathing													
Take Baby's Temperature													
Take Baby's Weight													
Monitor the Baby in order to Take Appropriate Action for Special Care (Requires Resuscitation)													
**Initiation of skin-to-skin**													

	Patient Yearly Number___________ From which register did you get the yearly number? ❑ Delivery ❑ Referred Out ❑ Admission				Patient Yearly Number _____________ From which register did you get the yearly number? ❑ Delivery ❑ Referred Out ❑ Admission				Patient Yearly Number __________ From which register did you get the yearly number? ❑ Delivery ❑ Referred Out ❑ Admission				General Activity (cannot be assigned to specific patient)
	PP 1	PP 2	PP 3	PP 4	PP 1	PP 2	PP 3	PP 4	PP 1	PP 2	PP 3	PP 4	
**Discussing Family Planning**													
**Explaining Danger Signs for mother and child**													
Group Discussion (if family planning and danger signs could not be observed)													
**Initiation of breastfeeding**													
**Administration of Vaccination**													

Appendix Table A3: Work Sampling Census
(see next page for start of PDF)

For peer review only

For peer review only - http://bmjopen.bmj.com/site/about/guidelines.xhtml

BB Work Sampling Census Sheet

Cover Page

A	Facility Code	
B	Date of Observation (DD/MMM/YYYY)	______/______/______
C	Notes about Work Sampling Census Observation:	
D	FADA Employee ID	

Work Sampling Census Sheet 29April2016

BB Work Sampling Census Page

Directions: update these numbers when you begin work sampling and in the last 5 minutes of every 2 hours of observation. Record the time and update the numbers based on FADA TL observation only and not official record

Hour	Start	Start + 2	Start + 4	Start + 6	Start + 8	Start + 10				
Clock Time										
Number of Women Admitted During Previous 2 hours										
Number of Women CURRENTLY in Waiting Room										
Number of Women CURRENTLY in L&D										
Number of Women CURRENTLY in Recovery										
Number Women Discharged / Transferred / Died During Previous 2 hours										
Number of Birth Attendants CURRENTLY on Duty in L&D										
Number of Helpers CURRENTLY on Duty in L&D										

Work Sampling Census Sheet 29April2016

Appendix Table A4: Work Sampling Observation Tool
(see next page for start of PDF)

For peer review only

For peer review only - http://bmjopen.bmj.com/site/about/guidelines.xhtml

BB Work Sampling Observation Tool

A	**Facility Code**	
B	**Date of Observation (DD/MMM/YYYY)**	____/____/____
C	**Health Care Worker Unique ID**	
D	**Health Care Worker Cadre**	☐ Doctor ☐ L.H.V ☐ A.N.M ☐ Staff Nurse ☐ Other
E	**Years of Experience as a Health Worker**	________ Years ________ Months
F	**Years of Experience as a Health Worker at this health facility**	
G	**Did Health care Worker consent to Observation**	___Yes ___No
H	**Notes about Work Sampling Observation:**	
I	**Tool ID Code**	
J	**FADA Employee ID**	
K	**FADA Role**	☐ First Observer ☐ DQA Observer
L	**FADA Start Time**	______:______
M	**FADA End Time**	______:______

Patients Consented

**Yearly Number**	**From which register did you get the yearly number?**

BB Work Sampling Tool 10Jun2016

For peer review only

	WHO Safe Childbirth Checklist Activities																	Non WHO SCC Activities				
Observation Time	1. Mat Temp	2. Blood Pressure	3. Parto.	4. Checklist/ poster	5. Medication	6. Hand-wash gloves or alochol rub	7. Prep of EBS	8. Use neonatal bag mask	9. Referring a patient	10. Check Mother for bleeding	11. Examination of Newborn	12. Initiation of skin-to-skin	13. Discussing Family Planning	14. Danger signs	15. Group Discussion (if family planning and danger signs could not be observed)	16. Initiation of Breast-feeding	17. Confirmation of Vaccination	18. Non-CL Direct Patient Care	19. Admin. Duties	20. Break	21. Down-time	22. Unobserved
HOUR 1																						
:02																						
:04																						
:06																						
:08																						
:10																						
:12																						
:14																						
:16																						
:18																						
:20																						
:22																						
:24																						
:26																						
:28																						
:30																						
:32																						
:34																						
:36																						
:38																						
:40																						
:42																						
:44																						
:46																						
:48																						
:50																						
:52																						
:54																						
:56																						
:58																						

Notes:

For peer review only

Observation Time	1. Mat Temp	2. Blood Pressure	3. Parto.	4. Checklist/ poster	5. Medication	6. Hand-wash gloves or alochol rub	7. Prep of EBS	8. Use neonatal bag mask	9. Referring a patient	10. Check Mother for bleeding	11. Examination of Newborn	12. Initiation of skin-to-skin	13. Discussing Family Planning	14. Danger signs	15. Group Discussion (if family planning and danger signs could not be observed)	16. Initiation of Breast-feeding	17. Confirmation of Vaccination	18. Non-CL Direct Patient Care	19. Admin. Duties	20. Break	21. Down-time	22. Unobserved
HOUR 2																						
:00																						
:02																						
:04																						
:06																						
:08																						
:10																						
:12																						
:14																						
:16																						
:18																						
:20																						
:22																						
:24																						
:26																						
:28																						
:30																						
:32																						
:34																						
:36																						
:38																						
:40																						
:42																						
:44																						
:46																						
:48																						
:50																						
:52																						
:54																						
:56																						
:58																						

Notes:

For peer review only

Observation Time	1. Mat Temp	2. Blood Pressure	3. Parto.	4. Checklist/ poster	5. Medication	6. Handwash gloves or alochol rub	7. Prep of EBS	8. Use neonatal bag mask	9. Referring a patient	10. Check Mother for bleeding	11. Examination of Newborn	12. Initiation of skin-to-skin	13. Discussing Family Planning	14. Danger signs	15. Group Discussion (if family planning and danger signs could not be observed)	16. Initiation of Breast-feeding	17. Confirmation of Vaccination	18. Non-CL Direct Patient Care	19. Admin. Duties	20. Break	21. Down-time	22. Unobserved
HOUR 3																						
:00																						
:02																						
:04																						
:06																						
:08																						
:10																						
:12																						
:14																						
:16																						
:18																						
:20																						
:22																						
:24																						
:26																						
:28																						
:30																						
:32																						
:34																						
:36																						
:38																						
:40																						
:42																						
:44																						
:46																						
:48																						
:50																						
:52																						
:54																						
:56																						
:58																						

Notes:

For peer review only

Observation Time	1. Mat Temp	2. Blood Pressure	3. Parto.	4. Checklist/ poster	5. Medication	6. Hand-wash gloves or alochol rub	7. Prep of EBS	8. Use neonatal bag mask	9. Referring a patient	10. Check Mother for bleeding	11. Examination of Newborn	12. Initiation of skin-to-skin	13. Discussing Family Planning	14. Danger signs	15. Group Discussion (if family planning and danger signs could not be observed)	16. Initiation of Breast-feeding	17. Confirmation of Vaccination	18. Non-CL Direct Patient Care	19. Admin. Duties	20. Break	21. Down-time	22. Unobserved
HOUR 4																						
:00																						
:02																						
:04																						
:06																						
:08																						
:10																						
:12																						
:14																						
:16																						
:18																						
:20																						
:22																						
:24																						
:26																						
:28																						
:30																						
:32																						
:34																						
:36																						
:38																						
:40																						
:42																						
:44																						
:46																						
:48																						
:50																						
:52																						
:54																						
:56																						
:58																						

Notes:

For peer review only - http://bmjopen.bmj.com/site/about/guidelines.xhtml

Observation Time	1. Mat Temp	2. Blood Pressure	3. Parto.	4. Checklist/ poster	5. Medication	6. Hand-wash gloves or alochol rub	7. Prep of EBS	8. Use neonatal bag mask	9. Referring a patient	10. Check Mother for bleeding	11. Examination of Newborn	12. Initiation of skin-to-skin	13. Discussing Family Planning	14. Danger signs	15. Group Discussion (if family planning and danger signs could not be observed)	16. Initiation of Breast-feeding	17. Confirmation of Vaccination	18. Non-CL Direct Patient Care	19. Admin. Duties	20. Break	21. Down-time	22. Unobserved
HOUR 5																						
:00																						
:02																						
:04																						
:06																						
:08																						
:10																						
:12																						
:14																						
:16																						
:18																						
:20																						
:22																						
:24																						
:26																						
:28																						
:30																						
:32																						
:34																						
:36																						
:38																						
:40																						
:42																						
:44																						
:46																						
:48																						
:50																						
:52																						
:54																						
:56																						
:58																						

Notes:

For peer review only - http://bmjopen.bmj.com/site/about/guidelines.xhtml

Obser-vation Time	1. Mat Temp	2. Blood Pressure	3. Parto.	4. Checklist/ poster	5. Medication	6. Hand-wash gloves or alochol rub	7. Prep of EBS	8. Use neonatal bag mask	9. Referring a patient	10. Check Mother for bleeding	11. Exami-nation of Newborn	12. Initiation of skin-to-skin	13. Discuss-ing Family Planning	14. Danger signs	15. Group Discussion (if family planning and danger signs could not be observed)	16. Initiation of Breast-feeding	17. Confirm-ation of Vaccination	18. Non-CL Direct Patient Care	19. Admin. Duties	20. Break	21. Down-time	22. Unob-served
HOUR 6																						
:00																						
:02																						
:04																						
:06																						
:08																						
:10																						
:12																						
:14																						
:16																						
:18																						
:20																						
:22																						
:24																						
:26																						
:28																						
:30																						
:32																						
:34																						
:36																						
:38																						
:40																						
:42																						
:44																						
:46																						
:48																						
:50																						
:52																						
:54																						
:56																						
:58																						

Notes:

For peer review only

Obser-vation Time	1. Mat Temp	2. Blood Pressure	3. Parto.	4. Checklist/ poster	5. Medication	6. Hand-wash gloves or alochol rub	7. Prep of EBS	8. Use neonatal bag mask	9. Referring a patient	10. Check Mother for bleeding	11. Exami-nation of Newborn	12. Initiation of skin-to-skin	13. Discuss-ing Family Planning	14. Danger signs	15. Group Discussion (if family planning and danger signs could not be observed)	16. Initiation of Breast-feeding	17. Confirm-ation of Vaccination	18. Non-CL Direct Patient Care	19. Admin. Duties	20. Break	21. Down-time	22. Unob-served
HOUR 7																						
:00																						
:02																						
:04																						
:06																						
:08																						
:10																						
:12																						
:14																						
:16																						
:18																						
:20																						
:22																						
:24																						
:26																						
:28																						
:30																						
:32																						
:34																						
:36																						
:38																						
:40																						
:42																						
:44																						
:46																						
:48																						
:50																						
:52																						
:54																						
:56																						
:58																						

Notes:

For peer review only

Obser-vation Time	1. Mat Temp	2. Blood Pressure	3. Parto.	4. Checklist/ poster	5. Medication	6. Hand-wash gloves or alochol rub	7. Prep of EBS	8. Use neonatal bag mask	9. Referring a patient	10. Check Mother for bleeding	11. Exami-nation of Newborn	12. Initiation of skin-to-skin	13. Discuss-ing Family Planning	14. Danger signs	15. Group Discussion (if family planning and danger signs could not be observed)	16. Initiation of Breast-feeding	17. Confirm-ation of Vaccination	18. Non-CL Direct Patient Care	19. Admin. Duties	20. Break	21. Down-time	22. Unob served
HOUR 8																						
:00																						
:02																						
:04																						
:06																						
:08																						
:10																						
:12																						
:14																						
:16																						
:18																						
:20																						
:22																						
:24																						
:26																						
:28																						
:30																						
:32																						
:34																						
:36																						
:38																						
:40																						
:42																						
:44																						
:46																						
:48																						
:50																						
:52																						
:54																						
:56																						
:58																						

Notes:

For peer review only

Appendix Table A5: General task categories in work sampling

General Group	Specific Activity
Checklist (CL)	Temperature<br>Blood pressure<br>Partograph<br>Paper checklist interaction<br>Medication<br>Handwashing<br>Prep of essential supplies<br>Neonatal bag mask<br>Referral<br>Check mother for bleeding<br>Examine newborn<br>Skin-to-skin initiation<br>Discuss family planning<br>Explain danger signs<br>Breastfeeding initiation<br>Confirm vaccination
Non-Checklist Clinical	Non-CL Direct Patient Care
Administrative	Admin. Duties
Downtime	Break<br>Downtime

For peer review only

For peer review only - http://bmjopen.bmj.com/site/about/guidelines.xhtml

Appendix Table A6: Task-time estimates for Essential Birth Practices

Essential Birth Practices	Time Source	Mean	SE	Min (seconds)	Max	Sample Size
Referral	Direct measurement	127	16	16	330	21
	Self-report	256	34	90	720	22
Temperature	Direct measurement	94	4	3	275	92
	Self-report	60	0	60	60	2
Check mother for bleeding	Direct measurement	92	12	2	320	37
	Self-report	178	22	60	300	14
Neonatal bag mask use	Direct measurement	76	16	6	300	22
	Self-report	175	23	60	300	11
Blood pressure	Direct measurement	74	2	20	165	126
	Self-report			N/A		N/A
Preparation of essential supplies	Direct measurement	59	18	8	436	31
	Self-report	202	29	120	600	18
Paper checklist interaction	Direct measurement	54	3	4	180	175
	Self-report	210	46	150	300	3
Explain danger signs	Direct measurement	40	4	8	109	41
	Self-report	258	14	60	600	65
Partograph	Direct measurement	38	3	9	88	38
	Self-report	164	16	30	240	15
Discuss family planning	Direct measurement	36	5	4	94	30
	Self-report	316	16	60	600	60
Examine newborn	Direct measurement	34	3	4	177	143
	Self-report	184	30	60	600	17
Assess baby's breathing	Direct measurement	31	6	10	60	9
	Self-report			N/A		N/A
Medication	Direct measurement	29	1	1	150	419
	Self-report	225	75	150	300	2
Handwashing	Direct measurement	29	1	1	81	208
	Self-report	180	11	150	210	6
Breastfeeding initiation	Direct measurement	24	2	6	60	43
	Self-report	223	35	120	420	9
Skin-to-skin initiation	Direct measurement	20	2	3	93	89
	Self-report			N/A		N/A
Weight	Direct measurement	18	2	5	44	35
	Self-report			N/A		N/A

Appendix Table A7: Heat map of self-reported most time-consuming Checklist tasks*

Checklist Activity	Rank 1	Rank 2 (Number of Respondents)	Rank 3	Total
Discussing family planning	41	16	3	60
Explaining danger signs	22	31	12	65
Prep of EBS	5	3	10	18
Check mother for bleeding	3	4	7	14
Initiation of breastfeeding	3	2	4	9
Referring a patient	3	11	8	22
Partograph	2	3	10	15
Use neonatal bag mask	2	1	8	11
Examination of newborn	1	4	12	17
Handwash gloves or alcohol rub	1	3	2	6
Checklist/ poster	0	2	1	3
Confirmation of vaccination	0	2	3	5
Medication	0	1	1	2
Temperature	0	0	2	2

*Number of staff reporting activity in each rank position; total staff interviewed = 83

Appendix Figure A1: Median labor and delivery ward patient load by hour (time of day) (1319 facility-hour observations; one facility-hour dropped in hour 20)

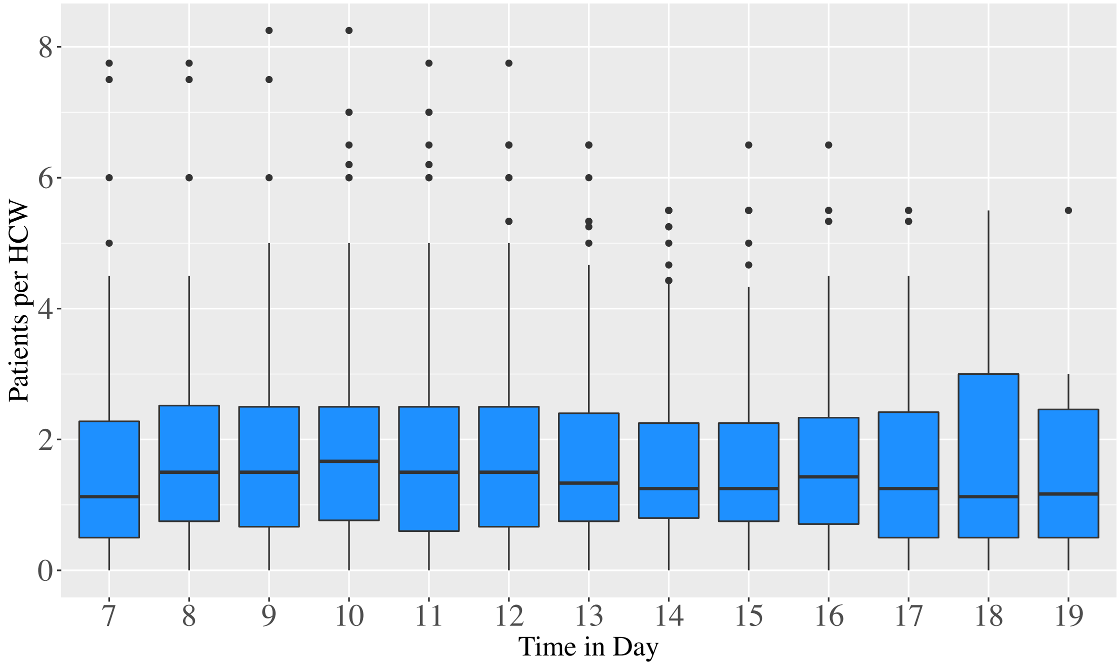

Appendix Figure A2: Median percent of hour on break by patient load per HCW (1320 facility-hour observations)

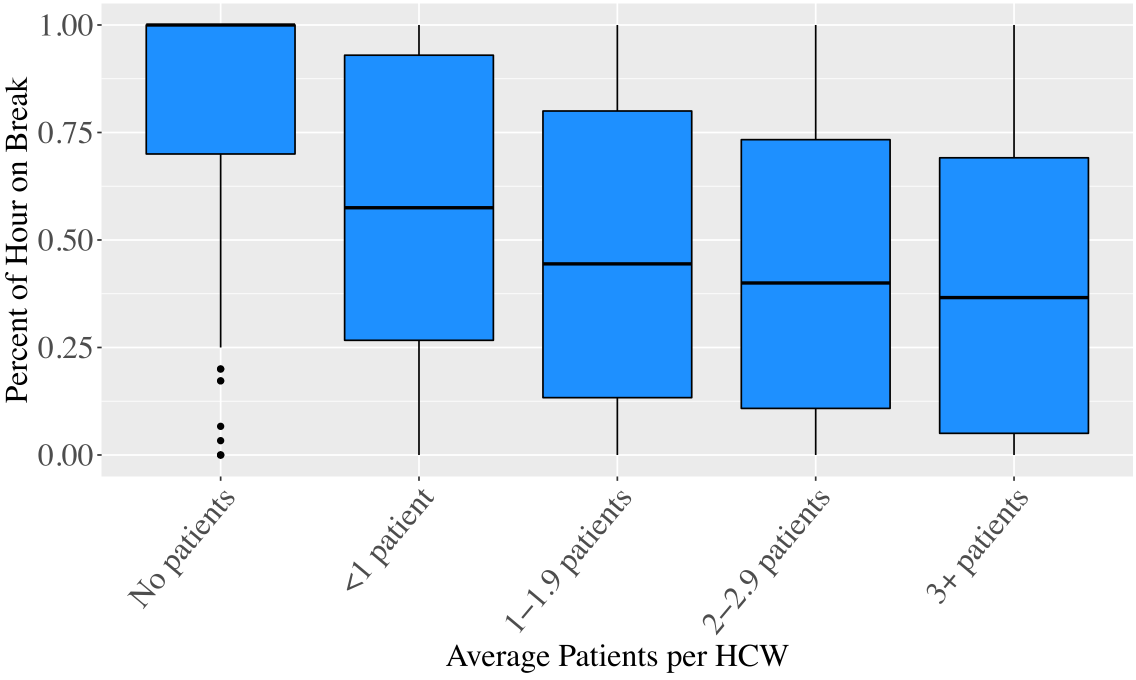

CONSORT 2010 checklist of information to include when reporting a randomised trial*

Section/Topic	Item No	Checklist item	Reported on page No
**Title and abstract**			
	1a	Identification as a randomised trial in the title	1
	1b	Structured summary of trial design, methods, results, and conclusions (for specific guidance see CONSORT for abstracts)	6
**Introduction**			
Background and objectives	2a	Scientific background and explanation of rationale	4-5
	2b	Specific objectives or hypotheses	6
**Methods**			
Trial design	3a	Description of trial design (such as parallel, factorial) including allocation ratio	6
	3b	Important changes to methods after trial commencement (such as eligibility criteria), with reasons	N/A
Participants	4a	Eligibility criteria for participants	6
	4b	Settings and locations where the data were collected	6
Interventions	5	The interventions for each group with sufficient details to allow replication, including how and when they were actually administered	6-8
Outcomes	6a	Completely defined pre-specified primary and secondary outcome measures, including how and when they were assessed	10-12
	6b	Any changes to trial outcomes after the trial commenced, with reasons	N/A
Sample size	7a	How sample size was determined	See protocol paper
	7b	When applicable, explanation of any interim analyses and stopping guidelines	N/A
Randomisation:			
Sequence generation	8a	Method used to generate the random allocation sequence	See protocol paper
	8b	Type of randomisation; details of any restriction (such as blocking and block size)	6 + abstract
Allocation concealment mechanism	9	Mechanism used to implement the random allocation sequence (such as sequentially numbered containers), describing any steps taken to conceal the sequence until interventions were assigned	See protocol paper
Implementation	10	Who generated the random allocation sequence, who enrolled participants, and who assigned participants to	See protocol

For peer review only - http://bmjopen.bmj.com/site/about/guidelines.xhtml
For peer review only

		interventions	paper
Blinding	11a	If done, who was blinded after assignment to interventions (for example, participants, care providers, those assessing outcomes) and how	N/A
	11b	If relevant, description of the similarity of interventions	N/A
Statistical methods	12a	Statistical methods used to compare groups for primary and secondary outcomes	Descriptive analysis in RCT
	12b	Methods for additional analyses, such as subgroup analyses and adjusted analyses	N/A
**Results**			
Participant flow (a diagram is strongly recommended)	13a	For each group, the numbers of participants who were randomly assigned, received intended treatment, and were analysed for the primary outcome	6 and 10-12
	13b	For each group, losses and exclusions after randomisation, together with reasons	N/A
Recruitment	14a	Dates defining the periods of recruitment and follow-up	7
	14b	Why the trial ended or was stopped	7
Baseline data	15	A table showing baseline demographic and clinical characteristics for each group	See main RCT results paper
Numbers analysed	16	For each group, number of participants (denominator) included in each analysis and whether the analysis was by original assigned groups	See main RCT results paper
Outcomes and estimation	17a	For each primary and secondary outcome, results for each group, and the estimated effect size and its precision (such as 95% confidence interval)	See main RCT results paper
	17b	For binary outcomes, presentation of both absolute and relative effect sizes is recommended	N/A
Ancillary analyses	18	Results of any other analyses performed, including subgroup analyses and adjusted analyses, distinguishing pre-specified from exploratory	All pre-specified measures
Harms	19	All important harms or unintended effects in each group (for specific guidance see CONSORT for harms)	N/A
**Discussion**			
Limitations	20	Trial limitations, addressing sources of potential bias, imprecision, and, if relevant, multiplicity of analyses	12-15
Generalisability	21	Generalisability (external validity, applicability) of the trial findings	12-15
Interpretation	22	Interpretation consistent with results, balancing benefits and harms, and considering other relevant evidence	12-15

For peer review only

**Other information**			
Registration	23	Registration number and name of trial registry	In submission fields
Protocol	24	Where the full trial protocol can be accessed, if available	Published manuscript included in submission
Funding	25	Sources of funding and other support (such as supply of drugs), role of funders	In submission fields

*We strongly recommend reading this statement in conjunction with the CONSORT 2010 Explanation and Elaboration for important clarifications on all the items. If relevant, we also recommend reading CONSORT extensions for cluster randomised trials, non-inferiority and equivalence trials, non-pharmacological treatments, herbal interventions, and pragmatic trials. Additional extensions are forthcoming: for those and for up to date references relevant to this checklist, see www.consort-statement.org.

For peer review only

For peer review only - http://bmjopen.bmj.com/site/about/guidelines.xhtml
